# Mitochondria-localized circRNA BRAKE restrains adipogenesis under nutrient overload by disrupting ATP synthase assembly in mice

**DOI:** 10.1038/s41467-026-78378-z

**Published:** 2026-10-13

**Authors:** Yanrui Huang, Tianxin Zhu, Xiaosi Hong, Yulin Yang, Jianyou Liao, Jiayu Liu, Chunni Li, Wei Wang, Sifan Chen, Phei Er Saw, Yanbing Li, Haipeng Xiao, Meng Ren, Shicheng Su, Yanrui Huang

**Affiliations:** 1 Department of Endocrinology, The First Affiliated Hospital, Sun Yat-sen University, Guangzhou, 510080 China; 2 Department of Endocrinology, Sun Yat-sen Memorial Hospital, Sun Yat-sen University, Guangzhou, 510120 China; 3 Medical Research Center, Sun Yat-sen Memorial Hospital, Sun Yat-sen University, Guangzhou, 510120 China; 4 Breast Tumor Center, Sun Yat-sen Memorial Hospital, Sun Yat-sen University, Guangzhou, 510120 China; 5 Guangdong-Hong Kong Joint Laboratory for RNA Medicine, Sun Yat-sen Memorial Hospital, Sun Yat-sen University, Guangzhou, 510120 China

**Keywords:** Energy metabolism, Non-coding RNAs, Obesity, Type 2 diabetes

## Abstract

Impaired adipogenesis contributes to obesity-associated metabolic disorders, in which mitochondrial dysfunction plays an important role. Emerging evidence suggests that mitochondria-localized circular RNAs (circRNAs) influence mitochondrial function; however, whether they regulate adipogenesis and systemic metabolism is unclear. By profiling circRNAs in mitochondria isolated from the white adipose tissue (WAT) of obese male mice, we observe an upregulation of *Birc6*-circRNA Repressing Adipogenesis and Krebs-cycle Energy (BRAKE) in mitochondria. In vivo, adipocyte-specific circRNA BRAKE knockdown in male mice reduces body weight, restricts fat expansion, and improves insulin sensitivity. In vitro studies show that circRNA BRAKE inhibits adipogenesis. Mechanistically, circRNA BRAKE binds ATP5A and disrupts ATP synthase assembly, thereby reducing ATP production and mTORC1 signaling and suppressing adipogenesis. In omental WAT (oWAT) of patients with obesity, mitochondrial circRNA BRAKE levels are elevated and inversely correlated with mitochondrial bioenergetic function. Collectively, our data identify a mitochondria-localized circRNA that regulates energy metabolism and adipogenesis, suggesting that circRNA BRAKE may represent a potential therapeutic target for obesity-related metabolic diseases.

## Abstract (web summary)

This study reports that the circular RNA BRAKE is a mitochondria-localized regulator of adipogenesis. By disrupting ATP synthase assembly, circRNA BRAKE restrains adipocyte differentiation and contributes to metabolic dysfunction during obesity.

## Introduction

Obesity poses a major public health concern and is independently associated with increased risks of metabolic disorders and malignancies^1,2^. However, effective pharmacological interventions remain limited, highlighting the need to elucidate its underlying molecular mechanisms for precise management. A defining feature of obesity is excessive white adipose tissue (WAT) expansion driven by nutrient overload. Due to depot-specific heterogeneity, visceral WAT is more closely linked to adverse metabolic outcomes than subcutaneous WAT. Epididymal WAT (eWAT), as a major visceral WAT depot, plays an important role in systemic glucose and lipid homeostasis^3–5^. Excessive eWAT expansion often occurs through adipocyte hypertrophy or hyperplasia. Hyperplasia, driven by the proliferation and differentiation of adipocyte progenitor cells (APCs), helps accommodate excess lipid storage and maintain metabolic homeostasis, whereas hypertrophy, the enlargement of existing adipocytes, is often associated with hypoxia, inflammation, and dysfunction^6^. Adipogenesis, the key process underlying adipocyte hyperplasia, is therefore considered metabolically protective under physiological conditions^7^. However, APCs from individuals with obesity exhibit reduced adipogenic capacity^8,9^, while weight loss can partially restore this function^10^, supporting the potential of enhancing adipogenic capacity as a therapeutic strategy for obesity-associated metabolic dysfunction.

Mitochondrial function plays an essential role in adipogenesis. In addition to supplying essential energy and substrates such as acetyl-CoA for adipogenesis, mitochondria also participate in its regulation through transcription factors, including PPARγ and C/EBPα^11^. In individuals with obesity, adipose tissue exhibits mitochondrial dysfunction^12^, and APCs display reduced mitochondrial content along with diminished adipogenic capacity^9^, highlighting the critical role of mitochondrial function in regulating adipogenesis. Yet the molecular mediators of this regulation are still largely unclear.

Circular RNAs (circRNAs), a group of noncoding RNAs with high stability and tissue specificity, display distinct expression patterns within adipose depots of mice and humans with obesity^13,14^. Moreover, circRNA expression changes dynamically during adipocyte differentiation, suggesting their involvement in adipogenesis^13,15^. Recent studies have revealed that mitochondria harbor diverse circRNAs, including both mitochondria-encoded circRNAs and nuclear genome-derived circRNAs that are subsequently transported into mitochondria^16,17^. Emerging evidence suggests that nuclear-encoded circRNAs participate in the regulation of mitochondrial homeostasis and energy metabolism. For instance, nuclear-encoded circSamd4 localizes to mitochondria and alleviates oxidative stress while maintaining mitochondrial homeostasis following myocardial infarction^18^, whereas circPUM1 stabilizes Complex III to enhance ATP production in esophageal cancer^19^. These findings suggest that nuclear-encoded circRNAs localized in mitochondria act as emerging modulators of mitochondrial activity and energy homeostasis; however, their functions in adipocytes and adipogenesis remain largely unknown.

Here, we investigated nuclear genome-derived circRNAs localized to adipocyte mitochondria and their roles in obesity. We identified *Birc6*-circRNA Repressing Adipogenesis and Krebs-cycle Energy (BRAKE) as markedly enriched in the mitochondria of both mice and humans with obesity. Loss of circRNA BRAKE in adipocytes led to l reduced body weight, adiposity, and circulating lipids, together with enhanced insulin sensitivity in mice. Mechanistically, circRNA BRAKE binds the mitochondrial protein ATP5A, thereby impairing ATP synthase assembly and ATP production. This reduction in ATP production attenuates mTORC1 signaling and ultimately suppresses adipogenesis. Analysis of clinical samples further revealed an inverse correlation between circRNA BRAKE levels and ATP5A protein levels in omental WAT from individuals with obesity. Together, these findings identify circRNA BRAKE as a mitochondria-localized regulator of energy metabolism and adipogenesis, uncover a circRNA-mediated mechanism contributing to obesity-associated metabolic dysfunction, and support further investigation of circRNA BRAKE as a potential therapeutic target.

## Results

### CircRNA BRAKE is specifically upregulated in mitochondria of eWAT from *ob/ob* mice

**Fig. 1:**
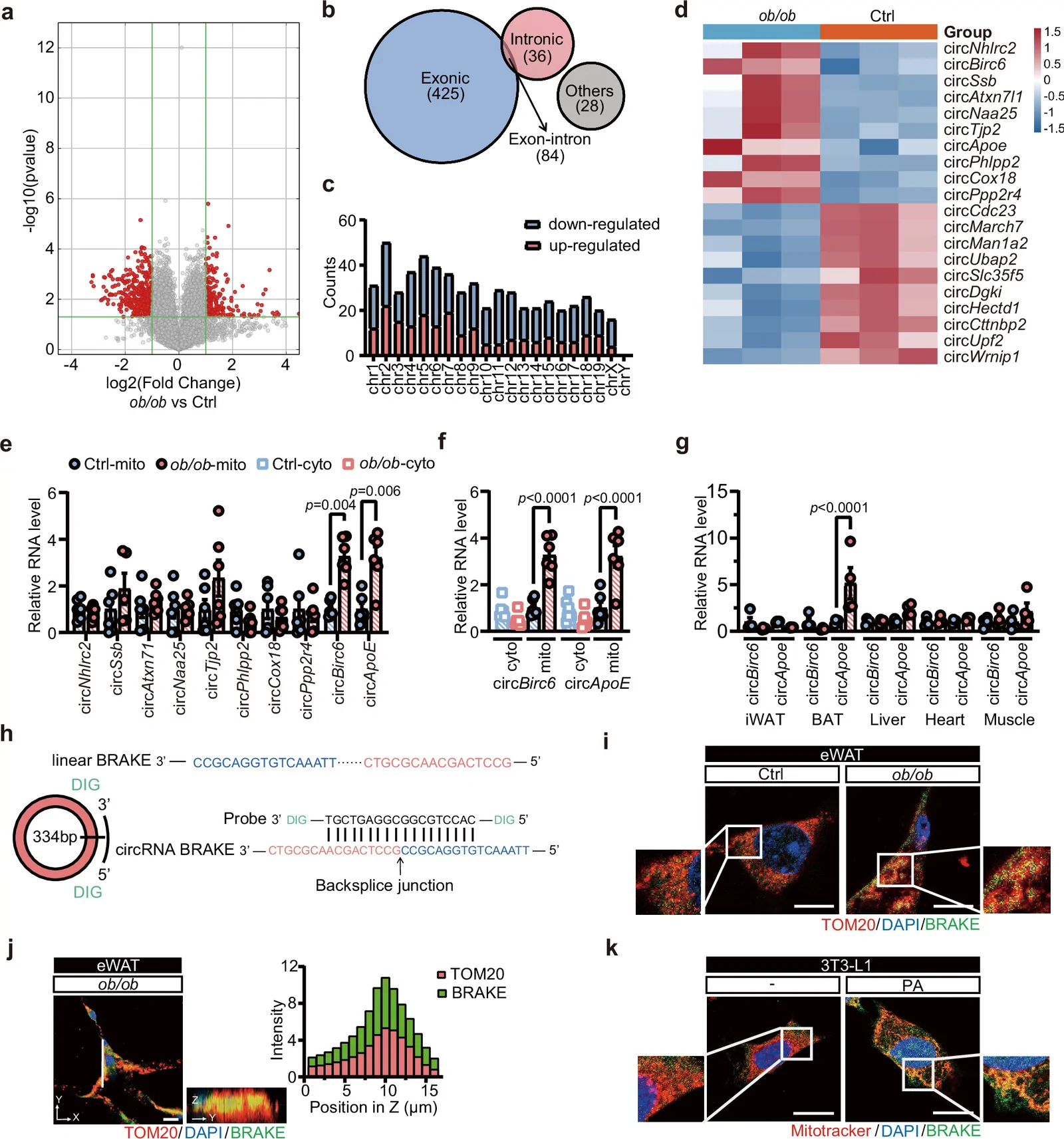
The expression of circRNA BRAKE is upregulated in the mitochondria of eWAT in *ob/ob* mice. **a–d** Mitochondria were isolated from eWAT of *ob/ob* and control (Ctrl) mice at 16 weeks (*n* = 3), followed by microarray-based profiling of circRNA expression. Fluorescence intensity values were averaged across three independent replicates. (**a**) Volcano plot presenting circRNAs displaying >2-fold differential expression with statistical significance (*P* < 0.05, two-tailed t-test). **b, c** Distribution of circRNAs according to formation type and chromosomal location. **d** Heatmap displaying the 10 most significantly upregulated and downregulated circRNAs. **e** Quantitative RT-PCR was used to verify mitochondrial levels of the selected circRNAs in eWAT from *ob/ob* and control mice (*n* = 6). **f** Quantitative RT-PCR validation of circRNA abundance in cytosolic and mitochondrial fractions from eWAT (*n* = 6), with *Actb* and *Mtco2* serving as reference markers for cytosolic and mitochondrial compartments, respectively. Mitochondrial circRNA values are the same data as those shown in E and are replotted here for comparison with the cytoplasmic fraction. **g** Quantitative RT-PCR validation of the indicated circRNA levels in mitochondria isolated from iWAT, BAT, liver, heart, and skeletal muscle of *ob/ob* and control mice (*n* = 4). **h** Schematic of the FISH probe designed to selectively detect circRNA BRAKE without hybridizing to its linear transcript. **i**–**k** FISH for circRNA BRAKE with mitochondrial membrane (TOM20) co-immunostaining in eWAT(**i**, **j**) (*n* = 3 biologically independent mice per group) and palmitate-treated 3T3-L1 cells (**k**) (*n* = 3 independent biological experiments). Panel (**j**) shows three-dimensional high-resolution confocal reconstructions with corresponding fluorescence intensity profiles plotted along the dashed Y-axis line. Scale bar, 20 μm. Each data point represents an independent biological replicate. All quantitative data are expressed as mean ± SEM. Statistical significance was analyzed by two-tailed Student’s *t*-test. Source data are provided as a Source Data file.

To identify nuclear genome-derived circRNAs that localize to adipocyte mitochondria and are associated with obesity, we analyzed epididymal white adipose tissue (eWAT) from *ob/ob* mice and C57BL/6 controls. Mitochondrial circRNA levels were analyzed using high-throughput microarrays, applying a cutoff of ≥2-fold change and P ≤ 0.05. In total, 205 circRNAs were upregulated, while 368 were downregulated in mitochondria from *ob/ob* mice (Fig. 1A). Of the differentially expressed circRNAs, 425 were exon-derived, while 36 and 84 originated from intronic regions and exon–intron junctions, respectively (Fig. 1B). These circRNAs were distributed across multiple chromosomes (Fig. 1C). From these datasets, we shortlisted 20 circRNAs with both pronounced differential abundance and high human–mouse sequence conservation (≥80%) (Fig. 1D).

qRT-PCR validation of the top 10 upregulated candidates confirmed that circ*Birc6* (mmu_circRNA_013298) and circ*ApoE* (mmu_circRNA_007066) were significantly enriched in mitochondria of *ob/ob* eWAT (Fig. 1E), while neither showed changes in the cytoplasmic fraction (Fig. 1F). Further profiling across metabolic tissues revealed that circ*ApoE* was also upregulated in brown adipose tissue (BAT) and liver, suggesting broader metabolic functions (Fig. 1G). In contrast, circ*Birc6*, hereafter referred to as circRNA BRAKE (*Birc6-*circRNA Repressing Adipogenesis and Krebs-cycle Energy), was specifically elevated in eWAT, indicating tissue-specific dysregulation and a potential role in regulating adipose function under obese conditions. To further quantify its mitochondrial abundance, we performed absolute qRT-PCR in eWAT-derived APCs. CircRNA BRAKE copy numbers in mitochondrial fractions and cells were significantly higher in APCs from *ob/ob* mice (Fig. S1A, B) and HFD-fed mice (Fig. S1C, D) than in their respective controls. The purity of the corresponding mitochondrial fractions was confirmed in Fig. S1E, F.

To confirm mitochondrial localization, we used a biotinylated probe specific for the back-splice junction of circRNA BRAKE, thereby minimizing potential interference from the linear host transcript (Fig. 1H). RNA fluorescence in situ hybridization (FISH) combined with mitochondrial immunofluorescence showed marked circRNA BRAKE accumulation in eWAT mitochondria of *ob/ob* mice (Fig. 1I). Quantification showed that 63.76% ± 6.45% of endogenous circRNA BRAKE signals colocalized with mitochondria (Fig. 1J). Consistently, immunofluorescence staining in 3T3-L1 preadipocytes confirmed mitochondrial localization of circRNA BRAKE (Fig. 1K).

Together, these findings identify circRNA BRAKE as a mitochondria-localized circRNA specifically upregulated in obese eWAT, prompting us to further investigate its function and underlying mechanism.

### Characterization of circRNA BRAKE in mouse adipocytes

CircRNA BRAKE arises from the circularization of *Birc6* exons 63 and 64, resulting in a 334-bp transcript that matches circBase records (http://www.circbase.org/). Sanger sequencing validated the precise back-splice junction (Fig. S2A). Divergent but not convergent PCR primers amplified circRNA BRAKE from reverse-transcribed RNA (cDNA) but not genomic DNA (gDNA), confirming its circular junction (Fig. S2B). CircRNA BRAKE resisted RNase R digestion, as shown by Northern blot and RT-PCR (Fig. S2C, D), and was poorly detected with oligo(dT) primers, consistent with the absence of a poly(A) tail (Fig. S2E). Finally, following actinomycin D treatment, circRNA BRAKE displayed greater stability than linear BRAKE mRNA (Fig. S2F).

Together, these findings support circRNA BRAKE as a bona fide and stable circular RNA transcript.

### Elevated circRNA BRAKE are associated with mitochondrial and adipogenic dysfunction in obese eWAT

Mitochondrial dysfunction represents an important feature of adipose tissue in obesity^12^. Consistently, eWAT from 16-week-old *ob/ob* mice exhibited markedly abnormal mitochondrial morphology, with cristae nearly absent (Fig. S3A). Furthermore, obese eWAT showed significantly lower ATP production compared with controls (Fig. S3B), along with impaired basal respiration, reduced ATP-linked oxygen consumption, diminished spare respiratory capacity (Fig. S3C), and lower OXPHOS protein expression (Fig. S3D). Correlation analysis indicated a negative association between mitochondrial circRNA BRAKE levels and ATP production (Fig. S3E), suggesting that elevated mitochondrial circRNA BRAKE is associated with mitochondrial dysfunction in obese eWAT.

Previous studies have indicated a deficiency of adipogenic capacity in APCs from individuals with obesity and diabetes^20,21^. Consistently, APCs isolated from obese eWAT displayed reduced oxygen consumption (Fig. S3F) and formed smaller and fewer lipid droplets upon adipogenic induction (Fig. S3G), indicating impaired adipogenesis. Hematoxylin and eosin staining further confirmed that eWAT expansion in *ob/ob* mice was predominantly due to adipocyte hypertrophy rather than hyperplasia (Fig. S3H). Notably, eWAT-derived APCs from *ob/ob* mice expressed lower levels of the key adipogenic transcription factor PPARγ (Fig. S3I). During adipogenic differentiation, mitochondrial circRNA BRAKE levels were significantly higher in APCs from *ob/ob* mice than in control APCs on days 3 and 6, indicating differentiation-associated accumulation of circRNA BRAKE in mitochondria (Fig. S3J). Consistently, mitochondrial circRNA BRAKE levels were inversely correlated with PPARγ expression (Fig. S3K), suggesting that elevated mitochondrial circRNA BRAKE may contribute to impaired adipogenesis in *ob/ob* mice.

Together, these results show that circRNA BRAKE upregulation is closely associated with mitochondrial dysfunction and impaired adipogenesis in obese eWAT, implying that circRNA BRAKE may contribute to obesity-related adipose tissue remodeling.

### Construction of an adipose tissue–specific circRNA BRAKE knockdown mouse model

**Fig. 2:**
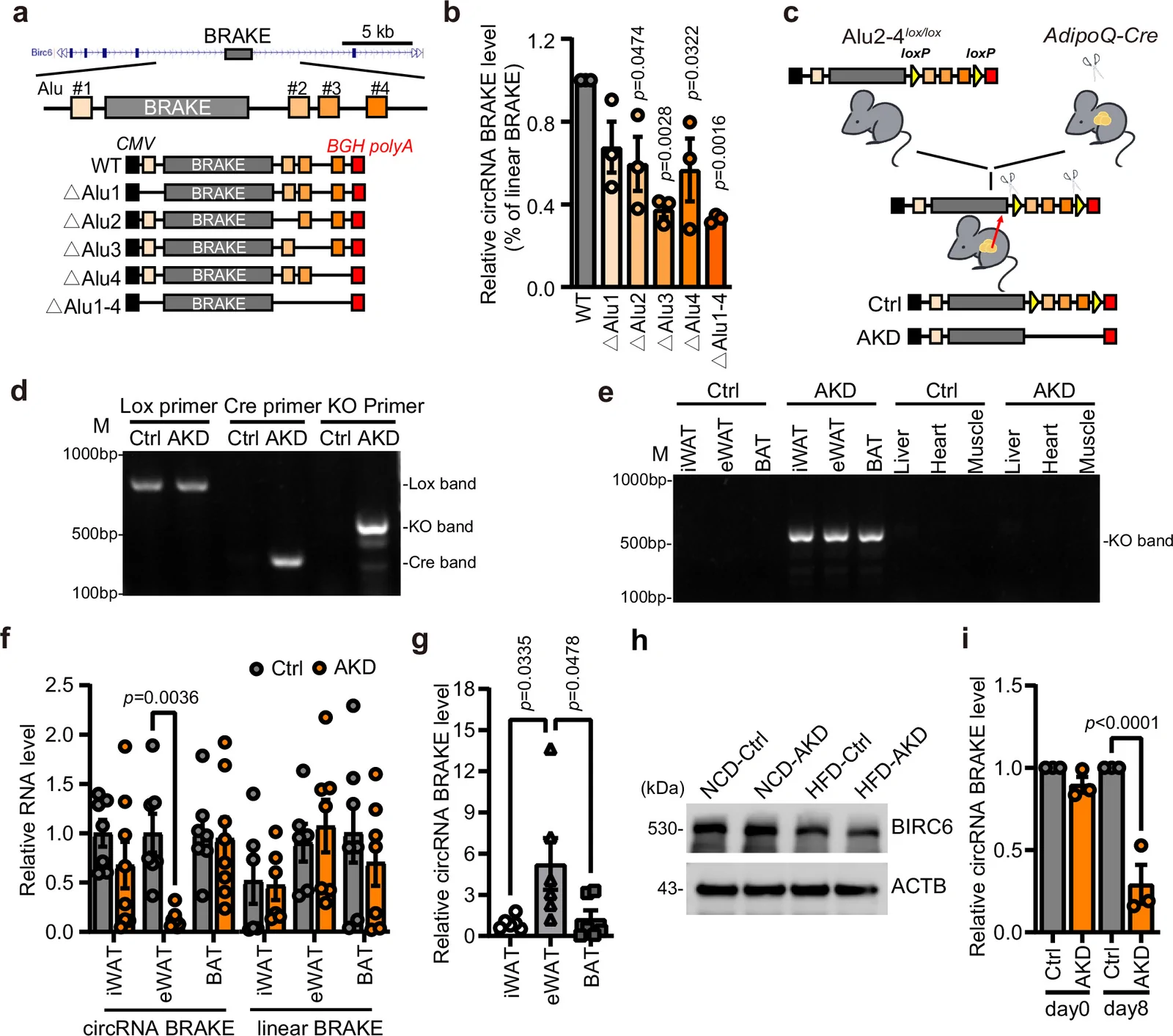
Construction of an adipose tissue-specific circRNA BRAKE knockdown mouse model. **a** Diagram illustrating that *Birc6* exons 63–64 harbor flanking Alu repeats, and the design of circRNA BRAKE expression constructs containing different genomic fragments (WT, △Alu1–△Alu4, and △Alu1–4) for circRNA recapitulation. The circRNA BRAKE genomic region, including upstream and downstream intronic sequences, was cloned into the pcDNA3.1 vector (WT), while specific Alu deletions were introduced into variant plasmids (△Alu1–△Alu4, △Alu1–4). **b** qRT-PCR measurement of circRNA BRAKE and linear BRAKE levels in HEK293 cells transfected with the above plasmids (*n* = 3 independent biological experiments), with linear BRAKE as the control. **c** Schematic of adipose-specific *AdipoQ-Cre*-mediated conditional knockdown mice of Alu2-4^lox/lox^. **d** Genotyping results of AKD and control mice (*n* = 3 independent biological mice per group). Alu2-4^lox/lox^ and *Adipo-Cre* genotypes were determined using tail DNA; Alu2-4 knockdown was confirmed using eWAT DNA. **e** Representative PCR analysis of Alu2-4 KO bands in iWAT, eWAT, BAT, liver, heart and muscle from AKD and control mice. **f** qRT-PCR quantification of circRNA BRAKE and linear BRAKE expression in mitochondrial fractions from iWAT, eWAT and BAT of AKD mice and control littermates. Samples with failed amplification or unreliable Ct values were excluded according to predefined quality-control criteria. The exact sample sizes are indicated in the graph(*n* = 6-8 per group). **g** Expression of mitochondrial circRNA BRAKE from different adipose depots was quantified by qRT-PCR (*n* = 6 independent biological mice). **h** Representative western blot for BIRC6 (*n* = 3 independent biological mice per group). **i** qRT-PCR detection of circRNA BRAKE in APCs (day 0) and differentiated adipocytes (day 8) (*n* = 3 independent biological experiments). Each data point represents an independent biological replicate. All quantitative data are expressed as mean ± SEM. Statistical significance was analyzed by two-way ANOVA with Šídák’s multiple comparisons test or two-tailed Student’s *t*-test. Source data are provided as a Source Data file.

To assess the role of circRNA BRAKE in WAT, an adipose-specific knockdown model was established. CircRNA BRAKE is formed by back-splicing of *Birc6* exons 63–64, so direct exon deletion would inevitably disrupt expression of the host transcript. As circRNA biogenesis often depends on flanking intronic inverted Alu elements^22^, we analyzed introns adjacent to exons 63–64 using the UCSC Genome Browser and identified four elements located closest to the circularizing exons, designated Alu1–Alu4, that were likely to mediate circularization (Fig. 2A). To assess their functional contribution, we constructed a wild-type plasmid containing the mouse BRAKE pre-mRNA sequence, as well as mutant plasmids with individual deletions of Alu1–4. Overexpression followed by qRT-PCR analysis revealed that deletion of Alu2, Alu3, or Alu4 markedly reduced circRNA BRAKE formation, indicating that these elements are important for efficient back-splicing (Fig. 2B).

To target circRNA BRAKE in adipose tissue, loxP sites were inserted around the closely spaced Alu2–4 elements, producing Alu2–4^lox/lox^ mice that were crossed with Adipo-Cre mice to obtain circRNA BRAKE adipose-specific knockdown (circRNA BRAKE^AKD^, hereafter AKD; Alu2–4^lox/lox^; Adipo-Cre). Littermate Alu2–4^lox/lox^ mice served as controls (Ctrl) (Fig. 2C). PCR analysis of eWAT from AKD mice detected specific Lox, KO, and Cre bands, confirming successful deletion of the Alu2–4 locus (Fig. 2D). No KO bands were found in other major metabolic tissues, demonstrating adipose-specific deletion (Fig. 2E).

The corresponding linear *Birc6* transcript (hereafter referred to as linear BRAKE) were unchanged across adipose depots, whereas circRNA BRAKE was markedly reduced only in eWAT (Fig. 2F), probably due to its low basal expression in iWAT and BAT (Fig. 2G). Consistently, BIRC6 protein levels were not altered in eWAT from AKD mice under either chow diet or high-fat diet conditions (Fig. 2H). These results indicate that the deletion of Alu2–4 elements selectively reduces circRNA BRAKE without suppressing host *Birc6* expression. Within eWAT, circRNA BRAKE downregulation occurred specifically in mature adipocytes but not in APCs (Fig. 2I), consistent with the predominant activity of Adipoq-Cre in mature adipocytes.

Together, these findings support AKD mice as a valid adipose-specific circRNA BRAKE knockdown model.

### Adipocyte-specific circRNA BRAKE knockdown improves metabolic homeostasis and reduces adiposity

**Fig. 3:**
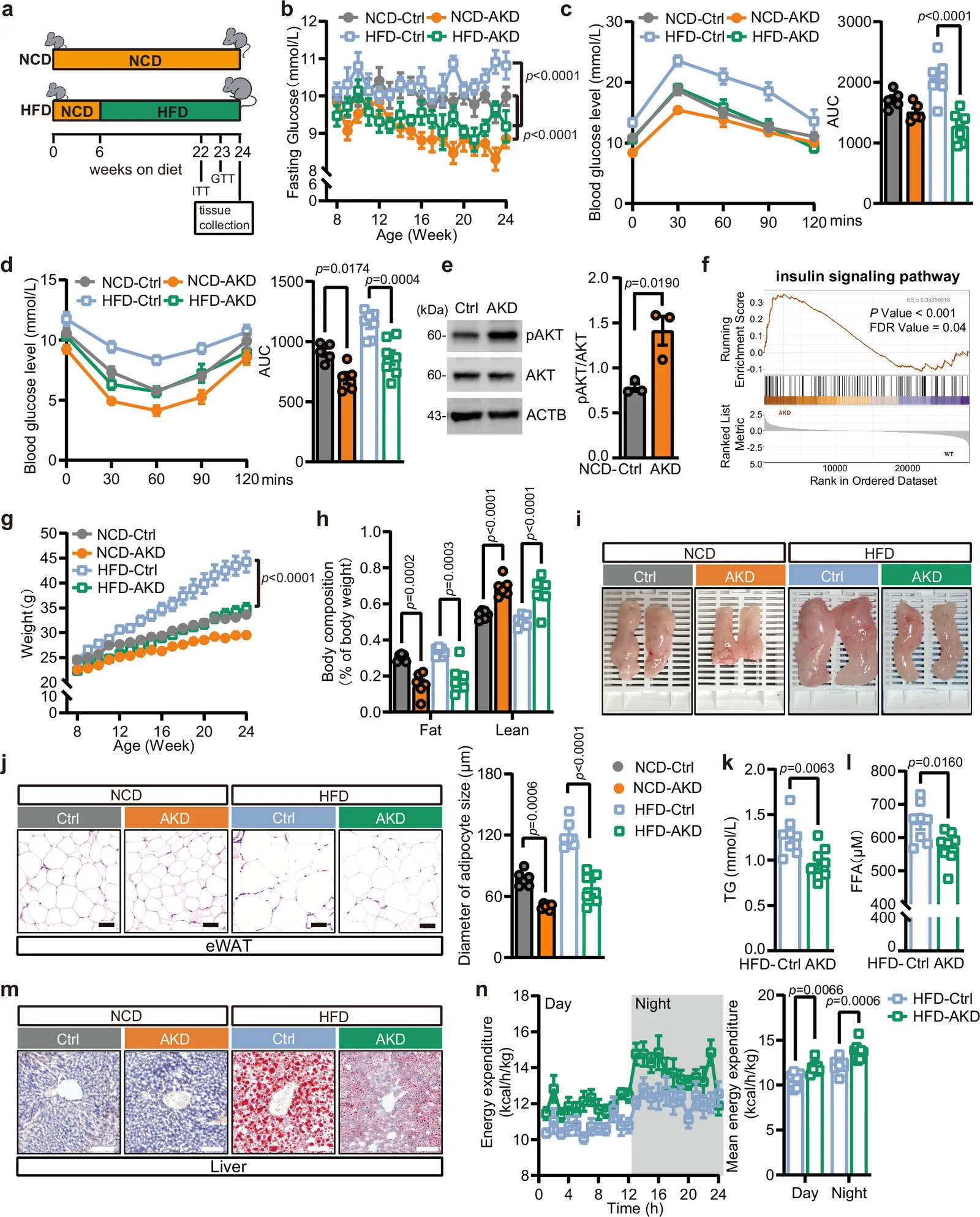
Adipocyte-specific circRNA BRAKE knockdown improves metabolic homeostasis and reduces adiposity. 24-week-old AKD male and control mice were maintained on normal chow diet (NCD) or high-fat diet (HFD). **a** Schematic diagram of the feeding. **b** Fasting glucose in NCD (*n* = 11) and HFD (*n* = 7) groups. **c** GTT glucose curves and incremental AUC in NCD (*n* = 5) and HFD (*n* = 7) groups. **d** ITT glucose curves and incremental AUC in NCD (*n* = 5) and HFD (*n* = 7) groups. **e** Representative western blots for total AKT and phospho-AKT (pAKT) in eWAT 15 min after insulin injection (2 U/kg), with quantification of pAKT-to-AKT ratios (*n* = 3 independent biological mice per group). **f** GSEA of insulin signaling pathway in differentiated adipocytes from eWAT of mice fed with NCD (*n* = 3). **g** Body weight gain in NCD (*n* = 11) and HFD (*n* = 7) groups. **h** Body composition of mice under NCD (*n* = 6) or HFD (*n* = 6). **i** eWAT morphology in NCD (*n* = 5) and HFD (*n* = 6) groups. **j** Representative H&E-stained eWAT images and average adipocyte diameter analysis. Each dot represents one mouse, calculated from multiple adipocytes measured across at least three random, non-overlapping fields (*n* = 6 mice per group). Scale bar: 50 μm. **k** Plasma TG levels under overnight fasting of mice under HFD (*n* = 8). **l**Plasma FFA levels under overnight fasting of mice under HFD (*n* = 8). **m** Oil Red O–stained liver sections (*n* = 3). Scale bar, 20 μm. **n** Energy expenditure of HFD-fed mice (*n* = 8). Each data point represents an independent biological replicate. All quantitative data are as mean ± SEM. Statistical significance was analyzed by two-way ANOVA followed by Šídák’s multiple comparisons test or two-tailed Student’s *t*-test. Source data are provided as a Source Data file.

To evaluate the metabolic impact of adipocyte-specific circRNA BRAKE knockdown, AKD and control mice received either NCD or HFD (Fig. 3A), and body weight along with fasting blood glucose were monitored regularly. AKD mice exhibited significantly lower fasting glucose than controls under both dietary conditions (Fig. 3B). Under NCD, AKD mice showed a modest improvement in glucose tolerance during the GTT, although the difference did not reach statistical significance. Insulin sensitivity during the ITT was significantly improved. Under HFD, both GTT and ITT were markedly improved in AKD mice (Fig. 3C, D). Notably, serum insulin levels were comparable between groups (Fig. S4A). We therefore examined insulin signaling in eWAT and found it was enhanced, as evidenced by increased insulin-stimulated AKT phosphorylation (Fig. 3E) and transcriptional upregulation of insulin signaling genes in differentiated APCs (Fig. 3F). Importantly, insulin-stimulated AKT phosphorylation were also activated in liver and skeletal muscle (Fig. S4B), suggesting that AKD improves systemic insulin sensitivity across multiple metabolic tissues, potentially downstream of improved adipose tissue function and lipid homeostasis.

In parallel, AKD mice displayed markedly lower body weight compared with controls under both NCD and HFD (Fig. 3G, S4C). Body composition analysis revealed a significant decreased fat mass proportion (Fig. 3H), indicating that reduced adiposity contributed substantially to the lower body weight. Gross examination showed smaller WAT depots(Fig. 3I, S4D), whereas histological analysis revealed smaller adipocytes in eWAT (Fig. 3J). HFD-fed AKD mice also showed lower serum triglycerides (TG) and free fatty acids (FFA) (Fig. 3K-L, S4E), and reduced hepatic lipid accumulation (Fig. 3M), suggesting that circRNA BRAKE knockdown improves systemic lipid metabolism under nutrient overload.

Metabolic cage analysis revealed increased energy expenditure in AKD mice, particularly during the dark phase under chow-fed conditions and during both the light and dark phases under HFD-fed conditions (Fig. 3N, S4F). Food intake was not reduced in AKD mice and was even increased during the dark phase under chow diet, whereas HFD-fed AKD mice showed comparable food intake to controls (Fig. S4G-H). Fuel utilization, as assessed by RER, remained largely unchanged (Fig. S4I). AKD mice also displayed higher spontaneous activity (Fig. S4J), suggesting that elevated activity, together with improved systemic metabolism, contributes to the increased energy expenditure.

Collectively, adipocyte-specific circRNA BRAKE knockdown enhances systemic insulin sensitivity, improves metabolic homeostasis, and elevates energy expenditure.

### Adipocyte-specific circRNA BRAKE knockdown enhances adipogenic capacity and mitochondrial function

**Fig. 4:**
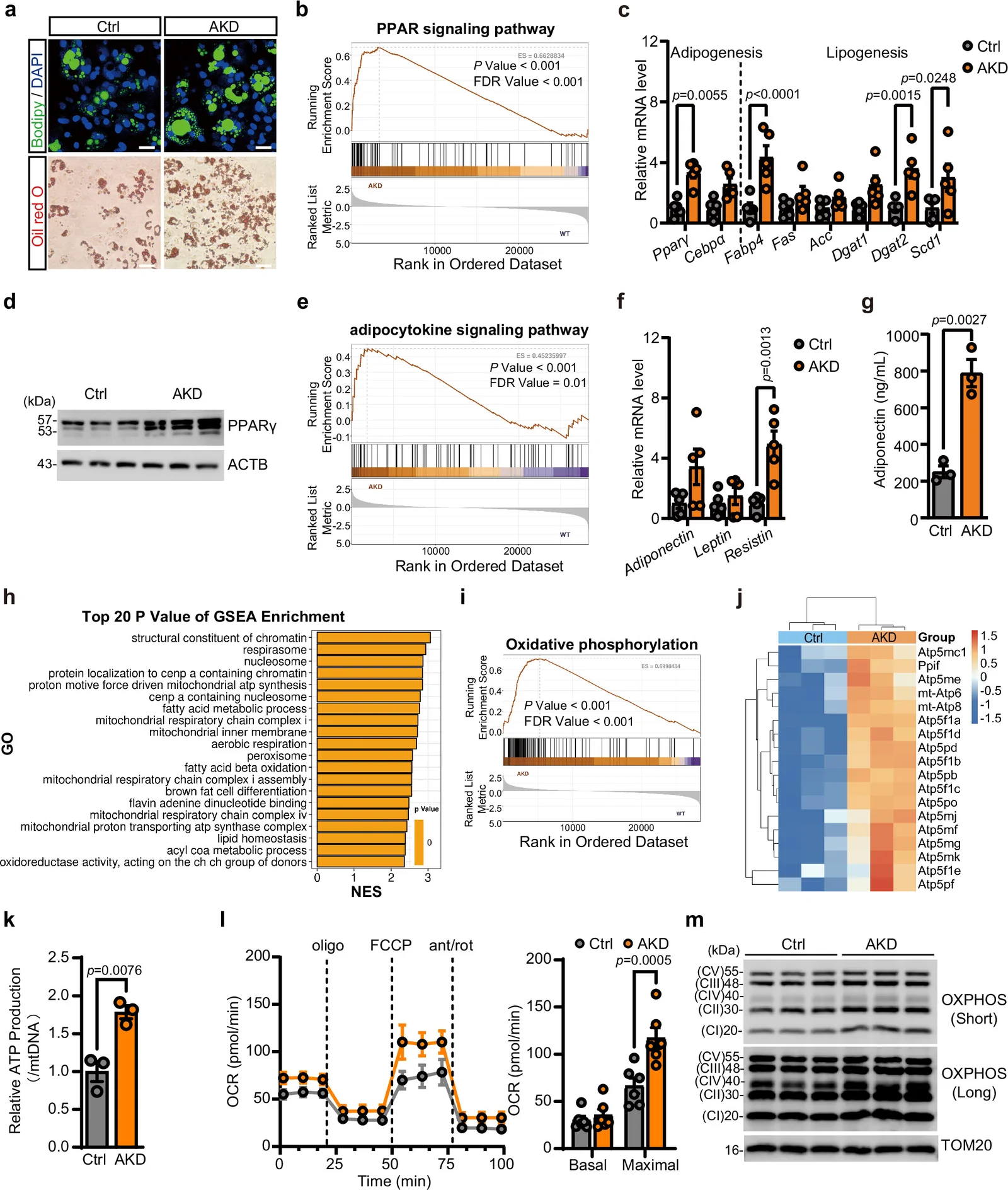
Adipocyte-specific circRNA BRAKE knockdown enhances adipogenic capacity and mitochondrial function. Differentiated adipocytes derived from eWAT of 8-week-old NCD-fed AKD and control mice were analyzed. Each dot represents adipocytes differentiated from one individual mouse. **a** Representative BODIPY and Oil Red O staining images (*n* = 3 independent biological mice per group). Scale bar, 20 μm. **b** GSEA of PPAR signaling pathway. **c** qRT-PCR of adipogenic and lipogenic genes (*n* = 4 independent biological mice per group). **d** Western blot of PPARγ protein (*n* = 3 independent biological mice per group). **e** GSEA of adipocytokines signaling pathway (*n* = 3 independent biological mice per group). **f** qRT-PCR of adipocytokines (*n* = 5 independent biological mice per group). **g** Adiponectin levels in culture supernatant (*n* = 3 independent biological experiments). **h** Top 20 of GSEA enrichment. **i** GSEA of oxidative phosphorylation. **j** Heatmap of ATP synthase subunit genes. **k** ATP production in differentiated adipocytes (*n* = 3 independent biological experiments). **l** Oxygen consumption rate (OCR) with basal and maximal respiration quantification (*n* = 6 independent biological mice per group). **m** Western blot analysis of mitochondrial OXPHOS complexes I–V (*n* = 3 independent biological mice per group). Each data point represents an independent biological replicate. All quantitative data are expressed as mean ± SEM. Statistical significance was analyzed by two-tailed Student’s *t*-test. Source data are provided as a Source Data file.

Next, we investigated whether the reduced adipose tissue mass in AKD mice was linked to changes in the adipogenic capacity. Because Adiponectin-Cre–mediated circRNA BRAKE knockdown occurs predominantly in mature adipocytes rather than undifferentiated APCs (Fig. 2I), this in vivo model does not directly assess the early stages of adipogenic differentiation in vivo. We therefore interpreted the ex vivo APC differentiation assays as the adipogenic potential of APCs isolated from AKD adipose tissue and further used in vitro knockdown and overexpression models to test the role of circRNA BRAKE during adipogenesis. Following adipogenic induction, AKD-derived APCs formed larger and more numerous lipid droplets (Fig. 4A).

Efficient adipogenesis is typically accompanied by increased lipid synthesis and adipokine secretion. Transcriptomic analysis of differentiated APCs showed that circRNA BRAKE knockdown significantly activated key lipid synthesis pathways, notably the PPAR signaling pathway (Fig. 4B). qPCR confirmed upregulation of lipid synthesis- and adipogenesis-related genes in AKD APCs (Fig. 4C), and PPARγ protein levels were also significantly increased (Fig. 4D). Additionally, transcriptomic analysis also revealed enrichment of adipokine-related pathways in differentiated AKD APCs (Fig. 4E), with qPCR validating significant upregulation of *resistin* mRNA(Fig. 4F) and ELISA showing increased adiponectin levels in the culture medium (Fig. 4G). Together, these results suggest that adipose tissue from AKD mice exhibits enhanced adipogenic capacity, accompanied by increased lipid accumulation, lipogenic gene expression, and adipokine secretion.

Transcriptomic analysis also revealed significant upregulation of pathways associated with mitochondrial respiration (Fig. 4H), including increased expression of oxidative phosphorylation (OXPHOS) genes (Fig. 4I) and ATP synthase subunits (Fig. 4J) in AKD APCs. Furthermore, ATP production was significantly increased in differentiated AKD APCs (Fig. 4K). Seahorse XF24 analysis showed an increased maximal oxygen consumption rate (OCR) in AKD APCs, while extracellular acidification rate (ECAR) and lactate levels remained unchanged (Fig. 4L, S4K, L). Consistently, OXPHOS protein levels were increased in mature adipocytes from AKD mice (Fig. 4M), suggesting enhanced mitochondrial oxidative capacity during adipocyte differentiation.

We next asked whether this mitochondrial bioenergetic phenotype was recapitulated in intact adipose tissue in vivo. The eWAT from AKD mice exhibited increased ATP production (Fig. S5A) and enhanced mitochondrial oxidative capacity (Fig. S5B), along with elevated circulating adiponectin levels (Fig. S5C). Despite the predominantly post-differentiation onset of Adiponectin-Cre-mediated circRNA BRAKE knockdown, the mitochondrial phenotype observed in differentiated AKD APCs was mirrored in intact eWAT, supporting the physiological relevance of the ex vivo findings.

Collectively, these findings suggest that adipose tissue from AKD mice exhibits enhanced mitochondrial oxidative phosphorylation and bioenergetic capacity, accompanied by increased adipogenic differentiation and lipid metabolic activity, while glycolysis remains largely unchanged. These results support a role for circRNA BRAKE in restraining adipogenic differentiation by limiting mitochondrial ATP production and oxidative capacity.

### CircRNA BRAKE links mitochondrial dysfunction to impaired adipogenesis in APCs

Given the limitation of the Adiponectin-Cre system, circRNA BRAKE knockdown occurs predominantly in mature adipocytes, precluding direct assessment of its role during the early stages of APC differentiation in vivo. To address this, we established circRNA BRAKE knockdown and overexpression models in APCs.

For knockdown, we designed siRNAs targeting the back-splice junction of exons 63–64 (Fig. S6A), which efficiently reduced circRNA BRAKE without affecting linear BRAKE levels (Fig. S6B). Given that mitochondrial circRNA BRAKE levels were elevated in obese adipose tissue, we further treated cells with palmitate (PA), a saturated fatty acid elevated in obesity and HFD^23,24^, to mimic pathological lipid overload. Under these conditions, circRNA BRAKE knockdown increased proliferation ability (Fig. S6C), ATP production (Fig. S6D), maximal OCR (Fig. S6E), and ECAR (Fig. S6F), without altering lactate levels (Fig. S6G). Upon adipogenic induction, circRNA BRAKE knockdown enhanced differentiation, with increased lipid droplet accumulation and elevated PPARγ protein expression (Fig. S6H–I).

For gain-of-function, we cloned exons 63–64 into a circular RNA expression vector (Fig. S6J), which increased circRNA BRAKE levels without affecting linear BRAKE (Fig. S6K). Lentivirus-mediated circRNA BRAKE overexpression suppressed cell proliferation (Fig. S6L) and ATP production (Fig. S6M), reduced OCR and ECAR (Fig. S6N–P), diminished lipid droplet accumulation (Fig. S6Q), and decreased PPARγ protein abundance (Fig. S6R).

Together, these findings suggest that circRNA BRAKE directly inhibits mitochondrial bioenergetics in preadipocytes, thereby limiting adipogenic differentiation.

### CircRNA BRAKE directly interacts with ATP5A

CircRNAs formed by exon circularization commonly function as microRNA (miRNA) sponges, serve as templates for protein translation, or bind RNA-binding proteins (RBPs)^25^. Using TargetScan and miRANDA to predict miRNA interactions, the top five candidates were found to have only limited binding sites, indicating that circRNA BRAKE is unlikely to serve as a miRNA sponge (Fig. S7A). Furthermore, Coding Potential Calculator (CPC) analysis indicated that circRNA BRAKE has no protein-coding open reading frames, supporting the low protein-coding potential of circRNA BRAKE (Fig. S7B).

**Fig. 5:**
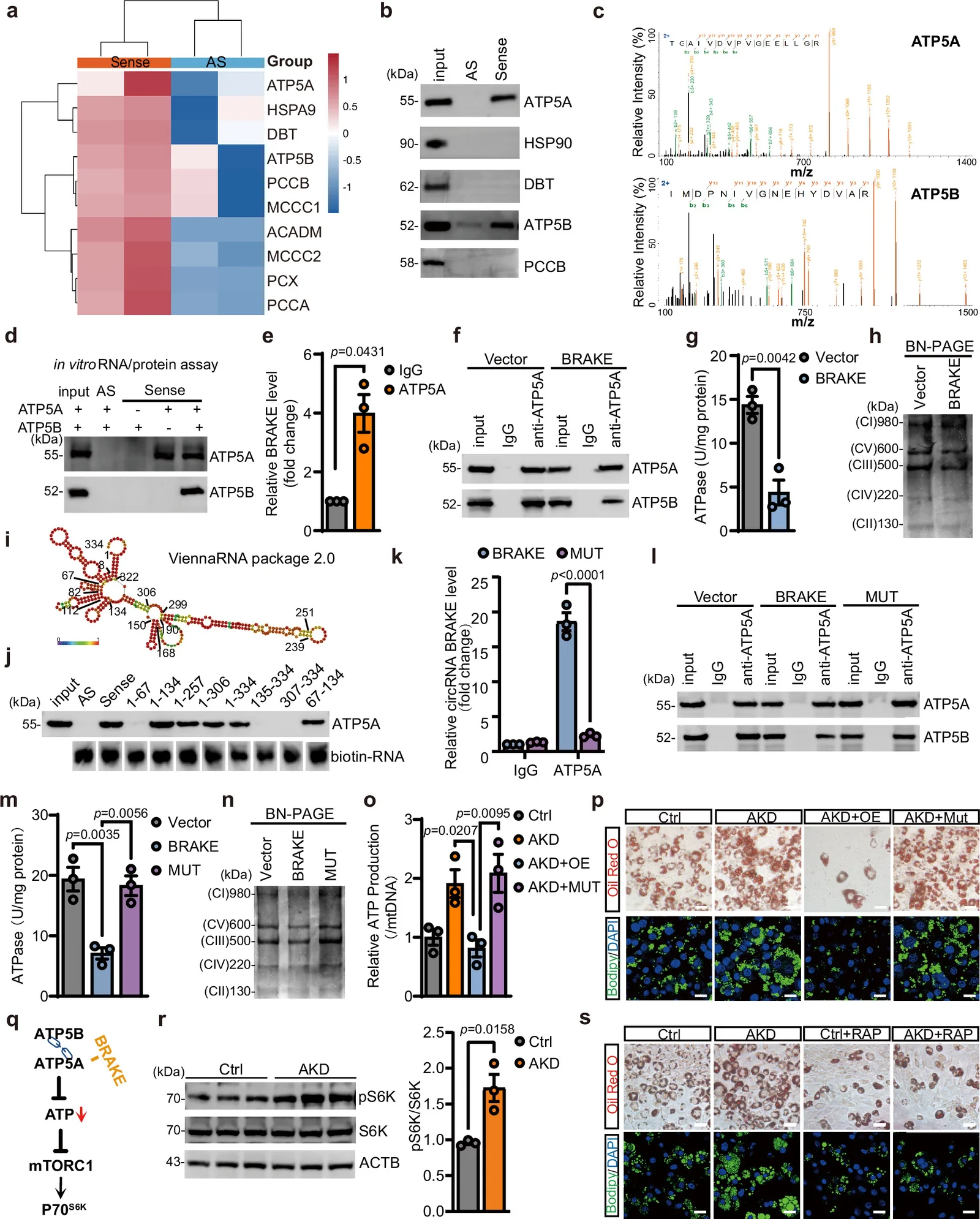
CircRNA BRAKE suppresses mitochondrial bioenergetics and adipogenesis via the ATP5A-mTORC1 pathway. **a** Heatmap of the top 10 mitochondrial proteins enriched in mitochondria from eWAT of *ob/ob* mice and control mice, captured with biotin-labeled circRNA BRAKE probes (*n* = 2). **b** Immunoblot showing ATP5A enrichment following circRNA BRAKE pull-down in mitochondria from eWAT of *ob/ob* mice (*n* = 3). **c** Mass spectrometry identification of ATP5A and ATP5B retrieved using biotin-labeled circRNA BRAKE probes in mitochondria from eWAT of *ob/ob* mice (*n* = 2). **d** Pull-down assay demonstrating direct interaction between circRNA BRAKE and recombinant ATP5A or ATP5B (*n* = 3). **e** RNA immunoprecipitation and qRT-PCR analysis quantifying circRNA BRAKE association with ATP5A in APCs (*n* = 3 independent biological experiments). **f** Immunoprecipitation analysis of the association between ATP5A and ATP5B in APCs transfected with empty vector (Vector) or circRNA BRAKE-overexpression plasmid (BRAKE) (*n* = 3). **g** ATP synthase activity in lysates of APCs transfected with Vector or BRAKE (*n* = 3 independent biological experiments). **h** BN-PAGE was performed in the isolated mitochondrial preparation in APCs transfected with Vector or BRAKE (*n* = 3). **i** Secondary structure of circRNA BRAKE predicted with ViennaRNA 2.0 under minimum free energy (MFE) criteria; base-pairing confidence is indicated by a color gradient, with warmer tones reflecting higher reliability. **j** Interaction of recombinant ATP5A with biotinylated circRNA BRAKE fragments of varying lengths assessed by in vitro RNA–protein binding assay (*n* = 3). **k** RNA immunoprecipitation and qRT-PCR evaluating binding of wild-type or mutant (MUT) circRNA BRAKE to ATP5A in APCs (*n* = 3 independent biological experiments). **l** Immunoprecipitation analysis of the association between ATP5A and ATP5B in APCs transfected with Vector, BRAKE or MUT (*n* = 3). **m** ATP synthase activity in lysates of APCs transfected with Vector, BRAKE or MUT (*n* = 3 independent biological experiments). **n** BN-PAGE was performed in the isolated mitochondrial preparation in APCs transfected transfected with Vector, BRAKE or MUT (*n* = 3). **o** ATP production in differentiated adipocytes of AKD mice transfected with wild-type or mutant circRNA BRAKE (*n* = 3 independent biological experiments). **p** BODIPY and Oil Red O staining images in differentiated adipocytes of AKD mice transfected with wild-type or mutated circRNA BRAKE (*n* = 3). Scale bar, 20 μm. **q** Proposed model summarizing ATP5A-mediated regulation of mTORC1 signaling. **r** Immunoblot of pS6K and total S6K in differentiated adipocytes of AKD mice and control mice, with quantification of pS6K/S6K ratio (*n* = 3 independent biological experiments). **s** BODIPY and Oil Red O staining in differentiated adipocytes of AKD mice and control mice after rapamycin (RAP) treatment (*n* = 3). Scale bar, 20 μm. Each data point represents an independent biological replicate. All quantitative data are expressed as mean ± SEM. Statistical significance was analyzed by two-way ANOVA with Šídák’s multiple comparisons test or two-tailed Student’s *t*-test. Source data are provided as a Source Data file.

Emerging evidence suggests that specific circRNAs can act as protein sponges through RBP binding, thereby modulating their target protein functions^26^. To investigate whether circRNA BRAKE binds RBPs in adipose tissue, we performed RNA–protein pulldown using a biotin-labeled circRNA BRAKE probe and mitochondrial protein extracts from eWAT of *ob/ob* mice, followed by mass spectrometry. We identified mitochondrial proteins significantly enriched in the circRNA BRAKE group (*P* < 0.05) and plotted a heatmap of the top ten differential proteins (Fig. 5A). Immunoblot validation of the top five candidates showed the enrichment of ATP5A and ATP5B in the circRNA BRAKE pulldown (Fig. 5B, C). RNA pull-down assays using purified recombinant proteins demonstrated direct binding of circRNA BRAKE to ATP5A but not ATP5B (Fig. 5D), consistent with previous studies suggesting ATP5A as a potential RBP in interactome capture assays^27,28^. ATP5A RNA immunoprecipitation further supported the association between endogenous circRNA BRAKE and ATP5A in primary APCs (Fig. 5E). Moreover, co-IP analysis showed that circRNA BRAKE overexpression reduced the ATP5A–ATP5B association in these APCs (Fig. 5F).

In mitochondria, ATP5A and ATP5B form the F1 sector of ATP synthase and cooperatively catalyze ATP synthesis^29^. Consistent with the reduced ATP5A–ATP5B association, circRNA BRAKE overexpression decreased ATP synthase activity in primary APCs (Fig. 5G). BN-PAGE analysis further showed a reduction in assembled ATP synthase (Fig. 5H), supporting the conclusion that circRNA BRAKE impairs ATP synthase assembly through its interaction with ATP5A.

These results indicate that circRNA BRAKE directly binds ATP5A, weakens ATP5A–ATP5B association, and thereby impairs ATP synthase assembly and activity, limiting mitochondrial bioenergetic capacity.

### CircRNA BRAKE suppresses mitochondrial bioenergetics and adipogenesis via the ATP5A–mTORC1 pathway

To identify the region of circRNA BRAKE that binds ATP5A, we first predicted its secondary structure using the ViennaRNA Package 2.0 (Fig. 5I). Given that circRNAs often interact with proteins via stem-loop structures, in vitro incubation of recombinant ATP5A with synthesized circRNA BRAKE stem-loop fragments identified nt67–134 as the direct binding region (Fig. 5J). We then deleted this fragment from circRNA BRAKE, cloned the resulting mutant into a pcDNA3.1 vector (MUT), and transfected it into primary eWAT SVF-derived APCs from AKD mice. qPCR confirmed that deletion did not affect the expression of circRNA BRAKE or its host gene (Fig. S7C), while ATP5A-RIP in differentiated AKD APCs further showed markedly reduced association of MUT with ATP5A (Fig. 5K), indicating that nt 67–134 is critical for the circRNA BRAKE–ATP5A interaction.

Functional analyses in the same primary AKD APC system indicated that wild-type circRNA BRAKE reduced the ATP5A–ATP5B association, whereas MUT largely abolished this effect (Fig. 5L). Consistently, MUT restored ATP synthase activity compared with wild-type circRNA BRAKE (Fig. 5M) and rescued the circRNA BRAKE-mediated impairment of ATP synthase assembly (Fig. 5N). These effects were accompanied by restoration of ATP production (Fig. 5O) and lipid droplet formation (Fig. 5P). These results suggest that circRNA BRAKE directly binds ATP5A via nt67–134, which is essential for its regulation of ATP synthase assembly, mitochondrial function, and adipogenic differentiation.

To further establish the functional relationship between circRNA BRAKE and ATP5A in a primary loss-of-function system, we performed siRNA-mediated knockdown experiments in primary eWAT SVF-derived APCs from control mice. Rescue experiments showed that ATP5A knockdown decreased ATP production and adipogenic capacity, whereas simultaneous circRNA BRAKE knockdown restored ATP levels (Fig. S7D), adipogenic differentiation (Fig. S7E), PPARγ expression (Fig. S7F), and adipokine-related gene expression (Fig. S7G, H).

ATP synthase generates ATP through oxidative phosphorylation, a central process in cellular energy metabolism. Increased mitochondrial ATP production may enhance cellular energy availability and thereby favor mTORC1 activation and downstream S6K phosphorylation during adipogenic differentiation^30^ (Fig. 5Q). Thus, the ATP5A–mTORC1–pS6K axis is critical for the regulation of energy homeostasis and adipogenesis. Consistently, circRNA BRAKE knockdown increased S6K phosphorylation in differentiated APCs, which was reversed by ATP5A knockdown (Fig. 5R and S7I). Moreover, oligomycin-mediated inhibition of ATP synthase attenuated both ATP production and S6K phosphorylation induced by circRNA BRAKE knockdown, supporting a functional link between ATP generation and mTORC1 activation (Fig. S7J).

To further determine whether mTORC1 activation mediates the adipogenic and lipid synthetic gene programs induced by circRNA BRAKE knockdown, we treated differentiated APCs with mTORC1 inhibitor rapamycin. Rapamycin attenuated the circRNA BRAKE knockdown-induced increases in free fatty acid (FFA) release, adipogenic and lipogenic gene expression, and PPARγ protein abundance (Fig. S8A–C). Consistently, rapamycin also attenuated the enhancement of adipogenic differentiation induced by circRNA BRAKE knockdown (Fig. 5S). Furthermore, rapamycin attenuated the AKD-induced increase in multiple OXPHOS proteins, suggesting that the enhanced OXPHOS protein abundance is at least partly associated with mTORC1-dependent mitochondrial functional maturation during adipocyte differentiation (Fig. S8D).

Importantly, these key mechanistic changes were also observed in vivo, as AKD eWAT exhibited increased ATP synthase activity and enhanced mTORC1 signaling, as indicated by elevated pS6K/S6K levels (Fig. S5D, E). Together, these findings support a functional circRNA BRAKE–ATP5A–ATP synthase–mTORC1 axis that links mitochondrial bioenergetics to adipogenic differentiation.

### CircRNA BRAKE negatively correlates with mitochondrial function in patients with obesity

**Fig. 6:**
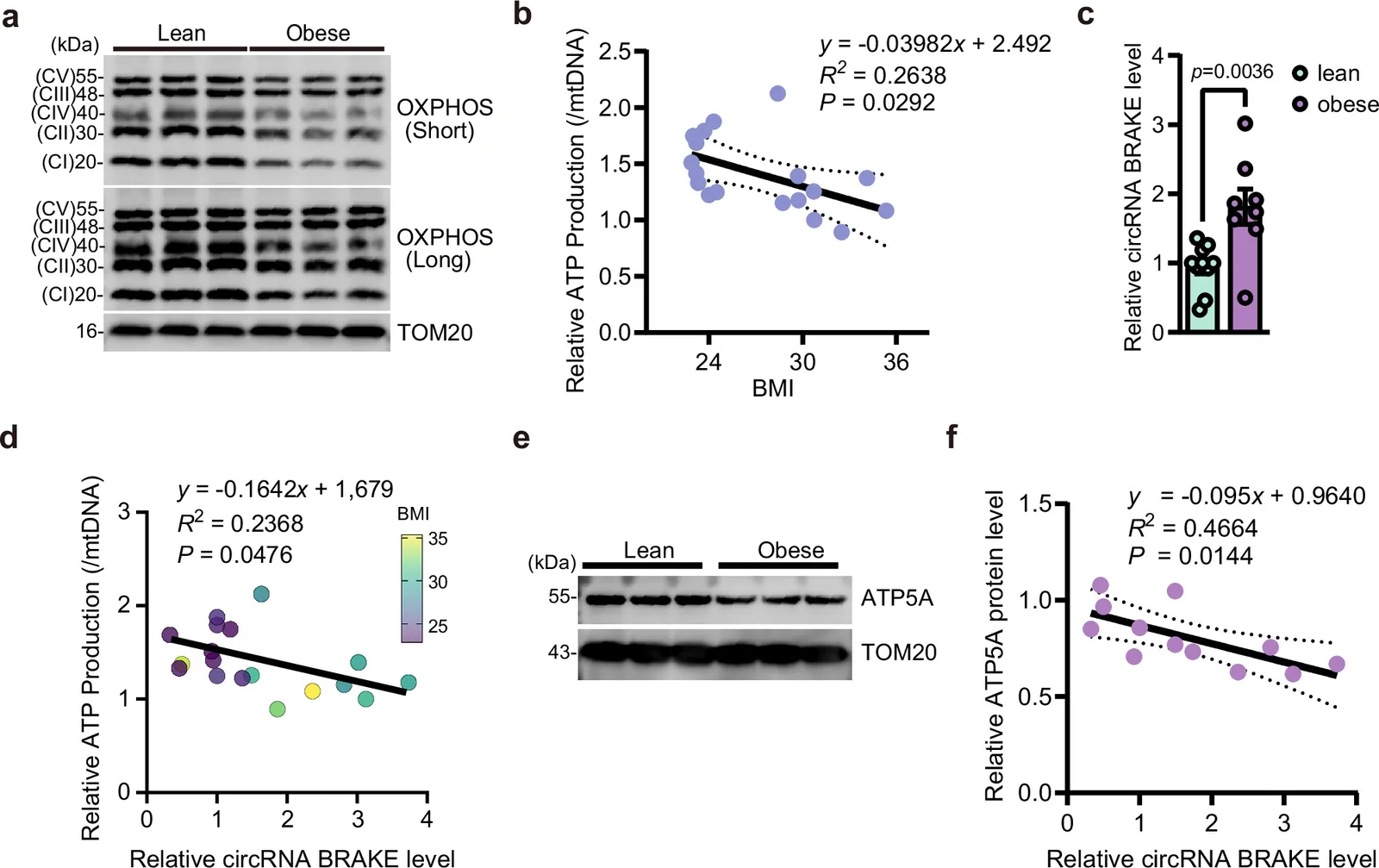
CircRNA BRAKE negatively correlates with mitochondrial function in patients with obesity. **a** Immunoblot analysis of OXPHOS complexes I–V in omental WAT (oWAT) from subjects with normal weight and obesity (*n* = 3). **b** Correlation between BMI and ATP production (*n* = 18). **c** qRT-PCR measurement of mitochondrial expression of circRNA BRAKE in oWAT from lean controls and individuals with obesity (lean: *n* = 10; obesity: *n* = 8). **d** Correlation between mitochondrial expression of circRNA BRAKE and ATP production. Dots of different colors represent different BMI categories (*n* = 18). **e** Immunoblot analysis of ATP5A protein in oWAT from subjects with normal weight and obesity (*n* = 3). **f** Correlation between mitochondrial expression of circRNA BRAKE and protein levels of ATP5A (*n* = 12). Each data point represents an independent biological replicate. All quantitative data are expressed as mean ± SEM. Two-sided Pearson correlation analysis was used to evaluate associations between variables. Statistical significance was analyzed by two-tailed Student’s *t*-test. Source data are provided as a Source Data file.

Previous studies have shown mitochondrial dysfunction, impaired adipogenesis, and elevated circRNA BRAKE in adipose tissue from obese mice. To assess the relevance of these findings to humans, omental WAT (oWAT) from individuals classified as obese (BMI ≥ 28 kg/m²) or lean (BMI ≤ 24 kg/m²) was analyzed. Obese oWAT exhibited reduced OXPHOS complex expression (Fig. 6A). ATP production was also reduced and was inversely correlated with BMI (*R²* = 0.707, *P* < 0.0001) (Fig. 6B).

Using UCSC and circBase databases, we identified the human homolog of circRNA BRAKE (hsa_circ_0005544, 853 bp), which is highly conserved with mouse circRNA BRAKE (Query Cover = 92%; Per. Ident = 83.12%) (Fig. S9). qRT-PCR analysis revealed significantly increased mitochondrial levels of circRNA BRAKE in obese oWAT (Fig. 6C), which negatively correlated with ATP production (Fig. 6D). Furthermore, ATP5A protein levels were markedly decreased in obese oWAT and exhibited an inverse correlation with mitochondrial levels of circRNA BRAKE (Fig. 6E, F).

These results suggest that elevated circRNA BRAKE levels in obesity correlate with reduced ATP5A and impaired mitochondrial bioenergetics, potentially contributing to adipose tissue dysfunction.

## Discussion

**Fig. 7:**
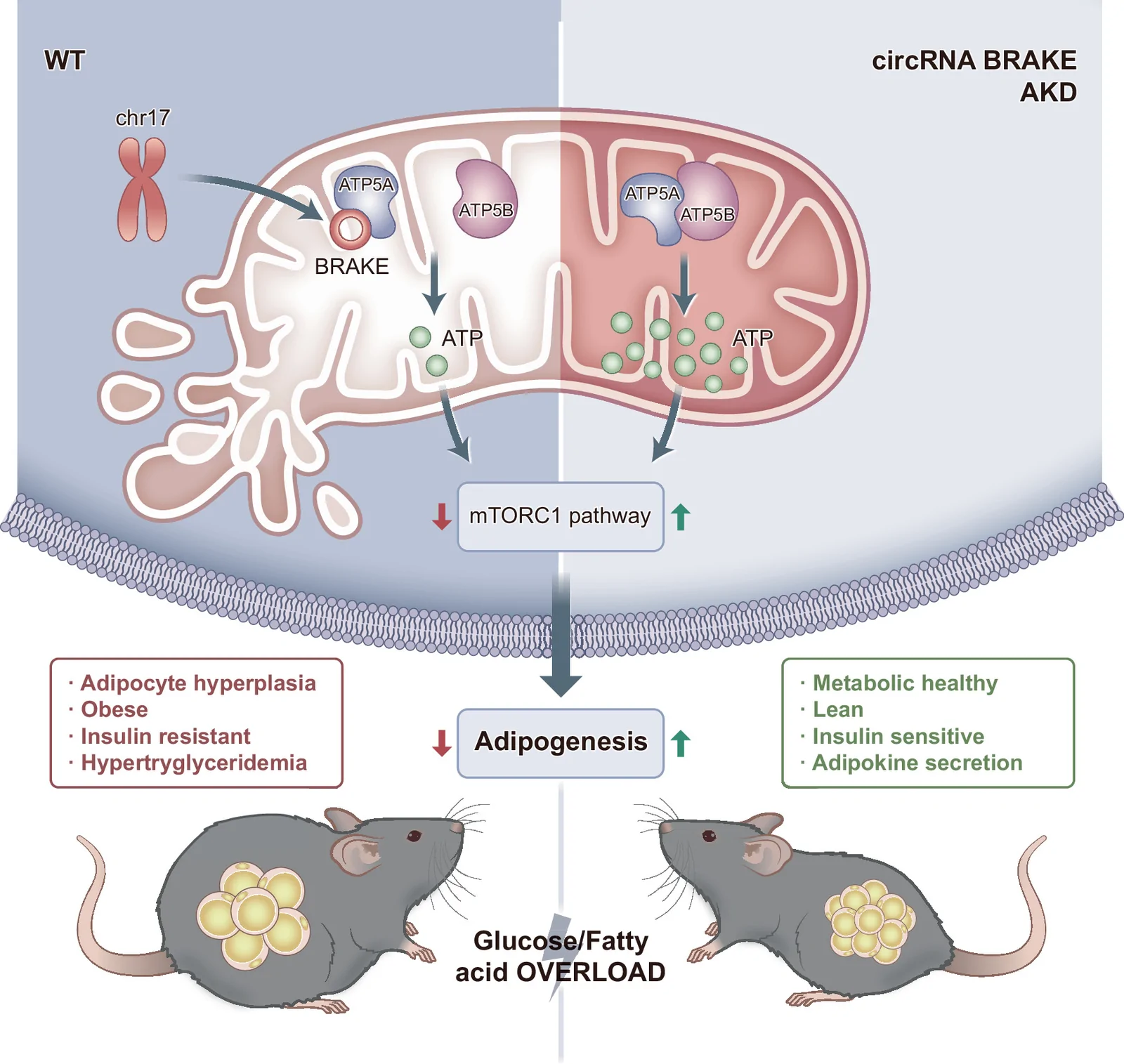
Working model of circRNA BRAKE regulates adipogenesis and energy metabolism in adipocytes. In obesity, nuclear-encoded circRNA BRAKE is enriched in WAT mitochondria. Elevated mitochondrial circRNA BRAKE binds ATP5A and disrupts ATP synthase assembly, thereby reducing ATP production and suppressing mTORC1 signaling. This mitochondrial dysfunction inhibits adipogenesis, contributing to adipose expansion and insulin resistance. Adipocyte-specific knockdown of circRNA BRAKE restores ATP synthase integrity, rescues ATP production and mTORC1 activity, and ameliorates obesity-associated metabolic disorders.

The global prevalence of obesity continues to increase, yet effective therapeutic strategies remain limited. Under nutrient excess, adipogenesis, the formation of newly formed adipocytes, acts as a protective mechanism that enables white adipose tissue to store energy and limit lipotoxicity, thereby preserving systemic metabolic health^31–33^. This process requires precise energy regulation, with mitochondria serving as central mediators^34^. Emerging evidence indicates that mitochondrial noncoding RNAs, particularly circular RNAs, function as stable transcripts in the regulation of energy homeostasis^18,19,35^. Here, we identify circRNA BRAKE as a key regulator of adipogenesis and mitochondrial bioenergetics. CircRNA BRAKE is markedly enriched in mitochondria of humans and mice with obesity, where it disrupts ATP synthase assembly, impairs energy metabolism, and consequently limits adipogenesis. By establishing an in vivo circRNA knockdown strategy, we generated an adipocyte-specific circRNA BRAKE knockdown model and provided evidence that circRNA BRAKE influences systemic metabolic homeostasis (Fig. 7). These findings highlight circRNA BRAKE as a potential contributor to obesity-associated metabolic dysfunction and a candidate for further therapeutic investigation.

Mitochondria harbor abundant circular RNAs that regulate mitochondrial function and homeostasis by facilitating nuclear-encoded protein import and modulating ROS output^16,17^. However, the involvement of mitochondria-localized circRNAs in obesity development remains unexplored. In this study, high-throughput profiling revealed numerous differentially expressed circRNAs in the mitochondria of obese mice. Subsequent screening and qPCR validation identified several nuclear genome-derived circRNAs with altered abundance in mitochondrial fractions, consistent with prior reports demonstrating nuclear-derived circRNA import. How nuclear genome-derived circRNA BRAKE is imported into mitochondria remains an important unresolved question. Previous studies have implicated PNPASE/PNPT1 in the mitochondrial import of selected nuclear-encoded RNAs, partly through the recognition of specific RNA stem-loop structures^36^. Moreover, mecciRNAs have been shown to interact with nuclear-encoded proteins, including RPA32, RPA70, and members of the hnRNPA family, and to facilitate their mitochondrial import^16^. Given that circRNA BRAKE directly binds ATP5A, one possibility is that circRNA BRAKE may associate with newly synthesized ATP5A before its mitochondrial translocation and subsequently be co-imported with ATP5A or retained within mitochondria through its association with ATP5A-containing complexes. This possibility warrants further investigation. Notably, circRNA BRAKE is highly conserved across human and mouse and is significantly elevated in the mitochondria of individuals with obesity. Its abundance was inversely correlated with ATP production, supporting a conserved association between circRNA BRAKE and mitochondrial bioenergetic dysfunction. These observations warrant further investigation of the therapeutic relevance of circRNA BRAKE in obesity.

Knockdown of circRNAs poses distinct challenges, as strategies must achieve specific inhibition without perturbing linear transcripts^37^. Current in vivo approaches for circRNA depletion mainly include RNA interference^38–40^, antisense circRNA overexpression^41^, CRISPR/Cas9-mediated deletion^42^, and Cre-lox–based conditional knockdown^43^. However, stable in vivo models that enable tissue-specific depletion of exon-derived circRNAs while preserving linear host-transcript expression remain limited. Since circRNA biogenesis often depends on flanking Alu repeats^22^, CRISPR/Cas9-mediated deletion of such elements, as shown for circHIPK3, can achieve stable and precise knockdown in vitro^44^. Building on these insights, we identified the critical Alu elements required for circRNA BRAKE circularization in vitro and subsequently used a Cre-lox system to delete them specifically in adipocytes in vivo. This strategy generated a stable adipose-specific circRNA BRAKE knockdown mouse model, while leaving linear BRAKE mRNA and BIRC6 protein unaffected. Because circRNA BRAKE and linear BRAKE are derived from the same genomic locus, they may share upstream transcriptional regulation; however, their final abundance is likely shaped by distinct post-transcriptional processes, including canonical splicing for linear BRAKE and back-splicing-dependent circularization for circRNA BRAKE. Thus, the preserved host-gene expression in AKD mice supports the notion that deletion of the Alu2–4 elements primarily disrupts circRNA BRAKE biogenesis rather than *Birc6* gene expression. Collectively, this work provides a circRNA knockdown strategy that integrates tissue specificity with structural precision, offering a feasible platform for metabolic research of circRNAs in vivo.

CircRNA–protein interactions are recognized as one of the major functions of circRNAs^45^. While many circRNAs interact with classic RBPs through defined binding motifs, their circular topology also confers distinct tertiary structures that enable binding to noncanonical proteins^46–49^. In this study, we revealed a direct interaction between circRNA BRAKE and the mitochondrial protein ATP5A. ATP5A is a core subunit of ATP synthase, and previous interactome capture experiments have implicated ATP5A as a potential RBP^27,28^. Our results further support ATP5A as an RBP that directly binds circRNA BRAKE. ATP synthesis by ATP synthase requires alternated assembly of α (ATP5A) and β (ATP5B) subunits into an α₃β₃ hexamer to drive the catalytic cycle^50^. Previous studies have shown that circRNAs can modulate protein–protein interactions by binding to specific partners^18,19,51,52^. Extending these observations, our data support a model in which circRNA BRAKE binds ATP5A and weakens ATP5A–ATP5B association and thereby impairs the assembly of the α₃β₃ hexamer essential for ATP synthase activity^50^. This interaction attenuates ATP synthase activity and cellular ATP production, highlighting a role for circRNAs in regulating mitochondrial energy metabolism and providing evidence for their roles within mitochondria. Our findings are also consistent with emerging evidence that mitochondria-associated circRNAs can interact with ATP synthase subunits. For instance, the mitochondrial genome-encoded circRNA SCAR was reported to bind ATP5B and regulate mPTP-associated mitochondrial ROS production. Recent work further demonstrated that multiple mecciRNAs interact with mPTP-related proteins, including ATP5B^17,53^. Compared with these mitochondria-encoded circRNAs, circRNA BRAKE represents a nuclear genome-derived mitochondria-localized circRNA that directly targets ATP5A, disrupts the ATP5A–ATP5B interaction, and regulates ATP synthase assembly in adipocytes. Thus, our study extends the functional spectrum of nuclear genome-derived mitochondria-localized circRNAs to include regulation of mitochondrial protein interactions, bioenergetic function, and systemic metabolism.

ATP production plays a critical role in adipocyte differentiation. During adipogenesis, mitochondrial protein expression and oxygen consumption increase markedly^54^. Exogenous ATP promotes adipogenesis^55^, whereas pharmacological inhibition of ATP synthase significantly reduces preadipocyte proliferation^56^. mTORC1, a central regulator of adipogenesis, senses nutrient and insulin signals and promotes adipogenesis via upregulation of PPARγ^57,58^. ATP directly binds mTORC1 and enhances its kinase activity, with the kinase domain forming a catalytic pocket that stabilizes ATP and enables phosphate transfer to substrates for downstream signaling^59,60^. In this study, circRNA BRAKE knockdown increased ATP production, activated mTORC1 signaling, and enhanced adipogenesis, whereas ATP synthase inhibition with oligomycin or mTORC1 inhibition with rapamycin attenuated these effects, supporting the notion that circRNA BRAKE negatively regulates adipogenesis by suppressing ATP–mTORC1 signaling.

Although rapamycin attenuated the increase in OXPHOS protein abundance induced by circRNA BRAKE knockdown, this finding does not fully explain how mitochondria-encoded subunits such as MTCO1 are coordinately increased. mTORC1 has been reported to promote mitochondrial activity and biogenesis partly through 4E-BP-dependent translation of nuclear-encoded mitochondrial proteins, which may account for the changes in nuclear-encoded OXPHOS subunits detected in our study^61^. However, MTCO1 is encoded by the mitochondrial genome and therefore cannot be explained by direct mTORC1-mediated cytosolic translation. Its coordinated increase may instead reflect broader mitochondrial functional maturation, nuclear–mitochondrial coordination, or assembly-dependent stabilization of OXPHOS complexes^62^. The precise mechanism linking circRNA BRAKE–ATP5A signaling to mitochondria-encoded OXPHOS protein abundance remains to be further investigated.

One limitation of our in vivo model is that Adiponectin-Cre–mediated circRNA BRAKE knockdown occurs predominantly in mature adipocytes, rather than in early adipocyte progenitors. Although our primary APC experiments support the role of circRNA BRAKE in regulating adipogenic differentiation, this model has limited ability to directly define the role of circRNA BRAKE during early adipogenesis in vivo. Nevertheless, the enhanced ATP synthase activity, increased mTORC1 signaling, and adipogenesis-related molecular changes observed in AKD eWAT indicate that key downstream components of this pathway are engaged in adipose tissue in vivo. Future studies using progenitor-targeting or inducible Cre models will be needed to determine the stage-specific function of circRNA BRAKE during adipose tissue development and remodeling.

In summary, mitochondria-localized circRNA BRAKE regulates adipocyte differentiation and systemic metabolic homeostasis by interacting with ATP5A to inhibit ATP synthase assembly and modulate mTORC1 activity. These observations offer an understanding of the molecular mechanisms contributing to obesity and related metabolic disorders, and point to circRNA BRAKE as a promising candidate for therapeutic intervention.

## Methods

### Statement of ethical regulations

The protocols of human studies obtained ethical clearance from the Ethics Committee at Sun Yat-sen University’s Memorial Hospital (Approval Number: SYSKY-2025-748-01). All animal experimental protocols were carried out in accordance with the institutional animal-welfare guidelines and received ethical approval from the Animal Ethics Committee of Sun Yat-sen University (Approval Number: SYSU-IACUC-2023-000315).

### Adipose samples and patients with obesity

Human omental adipose tissue (oWAT) samples were collected from lean subjects (BMI < 24) with HbA1c < 5.6%, and patients with obesity (BMI ≥ 28) with HbA1c < 6.5%. To minimize confounding by sex and age, subjects were enrolled at an approximately 1:1 male-to-female ratio, with comparable age distributions between sexes within each group. Sex was recorded from participants’ medical records. Sex-specific analyses were not performed because the study was not designed or powered to assess sex-dependent differences. All subjects were recruited in the Department of Hepatobiliary Surgery, Sun Yat-sen Memorial Hospital, Sun Yat-sen University. Those with active malignancies or inflammatory disorders were excluded from the study. Samples of oWAT were collected from patients undergoing laparoscopic cholecystectomy. Prior to inclusion, every participant provided written informed consent. The key characteristics of the human adipose tissue donors were provided in Supplementary Table S4 within the Supplementary Information.

### Animal studies

week-old male *ob/ob* (B6/JGpt-*Lep*^em1Cd25^/Gpt; strain T001461A) and C57BL/6 control mice (strain N000013) were obtained from GemPharmatech (Nanjing, China). To achieve selective suppression of circRNA BRAKE in adipose tissue while maintaining host gene integrity, C57BL/6J-background Alu2-4^lox/lox^ mice were generated by GemPharmatech (Nanjing, China) via homologous recombination, incorporating loxP sites into intron 64 of the *Birc6* locus. Alu2-4^*lox/lox*^ mice were generated by intercrossing heterozygous Alu2-4^+/lox^ mice. Their homozygous Alu2-4^lox/lox^ progeny were then bred to adiponectin-promoter–driven Cre transgenic (The Jackson Laboratory, Bar Harbor, ME, USA; strain 028020), yielding circRNA BRAKE^*AKD*^ (Alu2-4^lox/lox; *Adipo*-Cre^, abbreviated as AKD). The colony was backcrossed to C57BL/6 for at least 10 generations before experimentation. Adipocyte-specific circRNA BRAKE knockdown was confirmed using quantitative PCR with primers targeting *Birc6* intron 64 and Cre recombinase. The knockout (KO) primer set was used to detect fusion junctions resulting from targeted deletion in iWAT, eWAT, BAT, liver, heart, and skeletal muscle. Alu2-4^lox/lox^ served as controls (abbreviated as Ctrl). Both sexes were included in the initial studies and showed comparable metabolic and adipose tissue phenotypes; subsequent experiments were therefore conducted using male mice only to minimize potential variability associated with sex-dependent differences in adipose tissue distribution and metabolic phenotypes. Mice were housed under a 12-hour light/12-hour dark cycle at 25 °C with 60% relative humidity, with ad libitum access to standard chow and water.

Experimental mice were fed either a normal chow diet (NCD) (Guangdong Medical Laboratory Animal Center) containing 12% fat, 21% protein, and no cholesterol. For the high-fat diet (HFD) intervention, mice aged 6 weeks were maintained on HFD (Cat#12492, Research Diet) containing 60% fat, 20% protein, and 20% carbohydrates. Weekly measurements included body weight and fasting glucose levels, while body composition (lean/fat mass) was assessed via EchoMRI (LF90II, Bruker). After an overnight fast, tissues (eWAT, iWAT, BAT, liver, muscle, heart) and serum were collected for analysis. Serum lipid profiles (TC, TG, LDL-C, HDL-C) were quantified using a Synchron LX 20 system (Beckman Coulter).

### Preadipocyte isolation and differentiation

Epididymal white adipose tissue (eWAT) was harvested from 8-week-old male AKD and control animals. Specimens were first minced with scissors and then incubated under gentle shaking at 37 °C for 40 min in HBSS containing 4% fatty-acid-free BSA and 1 mg/ml collagenase II. After digestion, the cell suspension was passed through a 100 µm strainer and centrifuged (300 × *g*, 10 min) to isolate the adipocyte progenitor cells (APCs). Before plating, the pelleted APCs were resuspended in complete medium composed of high-glucose DMEM, 20% fetal bovine serum (FBS), and 1% penicillin–streptomycin. The culture medium was replaced at 72-hour intervals. Upon reaching confluence, APCs were detached using trypsin/EDTA and transferred to 6-well culture plates. Adipogenic differentiation was initiated 48 hours post-confluence using an induction cocktail (complete medium containing 0.25 µM dexamethasone, 0.17 µM insulin, and 135 µM IBMX) for 72 hours. Subsequently, cultures were maintained in differentiation medium (complete medium with 0.17 µM insulin). Adipocytes on day 8 of differentiation were collected for molecular analyses (RNA/protein isolation), cellular imaging, or metabolic function assessments. For rapamycin or oligomycin treatment, APCs were treated with either 10 nM rapamycin or 50 nM oligomycin, with vehicle-treated cells receiving an equal volume of DMSO starting on day 0 of differentiation, and the compounds were continuously present throughout the entire differentiation period.

### Glucose tolerance tests (GTTs) and insulin tolerance tests (ITTs)

GTTs and ITTs were performed on 24-week-old AKD and control male mice. GTTs were performed after measuring baseline fasting blood glucose, followed by an intraperitoneal bolus of 1 g/kg dextrose. For ITTs, 0.5 U/kg (NCD) or 1.0 U/kg (HFD) insulin was delivered via *i.p*.; tail-vein blood was then sampled at 30, 60 and 120 min and analyzed with a portable glucometer. (Roche).

### Indirect calorimetry

Metabolic cage analysis was performed on 24-week-old male AKD and control mice. Following a 24 h acclimation period, we measured food intake, carbon dioxide production (*V*CO_2_), oxygen consumption (VO_2_), pedestrian locomotion and energy expenditure in 24-wk-old male AKD and control mice (*n* = 5 per group for NCD-fed mice, *n* = 7 per group for HFD-fed mice) via a high-resolution metabolic phenotyping platform configured for small rodents (Promethion, Sable) at the Metabolic Innovation Center of Sun Yat-sen University. The RER was calculated as *V*CO_2_/*V*O_2_.

### Isolation of adipose tissue depots

Adipose depots were classified as white or brown adipose tissue based on morphological characteristics. White adipose tissue (WAT) was further categorized into subcutaneous and visceral compartments. The inguinal WAT (ingWAT), located cranial to the proximal hindlimb region, constitutes the predominant subcutaneous fat storage area in murine models. Epididymal WAT (eWAT) constitutes the most substantial accumulation among visceral depots. To prevent interference from adjacent reproductive structures, including the vas deferens, testicular vasculature, and epididymis, only the superior section of eWAT was harvested for analysis. Interscapular BAT was selected for examination due to its prominent size and anatomical position dorsal to the scapulae. During the dissection of interscapular BAT, meticulous care was taken to separate and discard any overlying subcutaneous adipose tissue.

### Isolation of mitochondria

Mitochondria were isolated from both adherent cultures and fresh tissue with the Mitochondria Isolation Kit (Cat#89874, ThermoFisher). Harvested material was resuspended in ice-cold Reagent A (800 µL) containing EDTA-free protease inhibitors, permeabilized by the addition of Reagent B (10 µL) and subjected to 5 min of intermittent vortexing (5 s every 60 s). After diluting with an equal volume of Reagent C (800 µL), lysates were cleared at 700 × *g* for 10 min at 4 °C; the resulting supernatant was transferred and centrifuged again at 12,000 × *g* for 15 min to sediment the organellar fraction. The final pellet was gently rinsed with Reagent C, kept on ice, and used immediately for subsequent analyses.

### TEM

Transmission electron microscopy (TEM) analysis was conducted at the Life Sciences Core Facility of Sun Yat-sen University. For sample preparation, eWAT was freshly dissected from mice and rapidly sectioned into small cubes (1 mm×1 mm×1 mm) in chilled PBS. Specimens were first immersed in a primary fixative (0.1 M sodium cacodylate containing 2% paraformaldehyde and 2.5% glutaraldehyde), and gently agitated for 30 min at room temperature, then transferred to 4 °C for an additional 90 min of fixation. Following three rinses in 0.1  M Na cacodylate buffer (pH 7.4), all downstream procedures were performed by the core facility personnel. Imaging was performed using an FEI Tecnai BioTWIN microscope operated at 80 kV, with digital acquisition via an SIS Morada 11-megapixel CCD camera and iTEM software (JEM-1400Flash, Rigaku).

### Histology and LD staining

Paraffin-embedded, formalin-fixed specimens were cut into 5 µm sections and subjected to hematoxylin–eosin staining. For hepatic lipid quantification, cryosections were immersed in Oil Red O working solution for 40 min. For adipogenic differentiation assessment, differentiated adherent cells were stained with either BODIPY 493/503 (1  µg/ml, Cat#D3922, Invitrogen) for 15min at room temperature, rinsed, and coverslipped with DAPI-containing medium, or with Oil Red O working solution for 40 min. At least three random, non-overlapping fields were analyzed from each mouse, and multiple adipocytes were measured in each field. The average adipocyte diameter was calculated for each mouse, and each data point represents the mean value from one individual mouse. Micrographs were captured on a Nikon Ni-U bright-field system or a Leica confocal scanner (TCS SP8 STED 3X).

### Fluorescence in situ hybridization (FISH) and mitochondrial immunofluorescence co-staining

Fresh eWAT sections and 3T3-L1 cells (CSTR: 16607.09.1101MOU-PUMC000155, National Biomedical Cell-Line Resource) were processed for circRNA BRAKE fluorescence in situ hybridization (FISH) combined with mitochondrial immunofluorescence staining. Briefly, samples were fixed in 4% paraformaldehyde, permeabilized with 0.05% Triton X-100, and subjected to an ascending ethanol series. After pre-hybridization in 2×SSC at 55 °C, samples were hybridized overnight with digoxigenin-labeled oligonucleotide probes (Synbio Technologies) targeting the back-splice junction of circRNA BRAKE. After stringent washing, FISH signals were detected using anti-digoxigenin-FITC (1:200, Cat#11207741910, Roche) and DAPI (Cat#0100, Southern Biotech). For mitochondrial co-immunostaining, sections were blocked with 0.5% BSA in PBST and incubated with anti-TOM20 antibody (1:500, Cat#11802-1-AP, Proteintech) and Cy3 goat anti-rabbit IgG (1:200, Cat#GB21303, Servicebio). Micrographs were captured on a Leica confocal scanner (TCS SP8 STED 3X), and three-dimensional reconstruction was performed using Leica software. The sequences of FISH probes were provided in Supplementary Table S2 within the Supplementary Information.

### Expression profile analysis of circRNAs

To enrich circular transcripts, total RNA was first digested with RNase R to remove linear species. The remaining circular RNAs were amplified and converted into fluorescent cRNA with the Arraystar Super RNA Labeling Kit (Cat#074301, Arraystar) according to the manufacturer’s instructions. The labeled probes were hybridized to Arraystar Mouse circRNA Array V2 slides for 17 h at 65 °C in a rotating chamber. After hybridization, the slides underwent stringent washes and were scanned on an Agilent G2505C microarray scanner.

Raw fluorescence data were processed with Agilent Feature Extraction software (version 11.0.1.1). Subsequent bioinformatics analysis included: Data normalization using the quantile method through the R software’s limma package; identification of statistically significant differentially expressed circRNAs via Volcano Plot analysis; detection of expression variations between sample groups through fold-change evaluation; and generation of hierarchical clustering heatmaps to visualize distinct circRNA expression profiles across experimental conditions. Mouse circRNA sequences were extracted from RNA-seq data. Sequences were aligned to the human genome (hg19) via the UCSC Genome Browser to retrieve syntenic coordinates. Human circRNAs associated with the same parent gene were subsequently retrieved from circBase using the corresponding gene symbol. Based on the UCSC alignment results, human circRNAs located in close proximity were identified, and their corresponding sequences were retrieved. Pairwise conservation between mouse and human circRNAs was then assessed by NCBI-BLASTn.

### Semiquantitative and absolute RNA quantification by real-time PCR (RT-PCR)

To extract RNA from mitochondria and cytoplasm, we first purified organelles using the Mitochondrial Isolation Kit (Cat#MP-007, Invitrogen) and then added Trizol reagent (Invitrogen). If it is necessary to extract total RNA from tissues, we will add Trizol reagent and use a cryogenic grinder (JXFSTPRP, Jing Xin) to crush the samples under liquid-nitrogen cooling; total RNA from cell samples can be directly recovered using Trizol reagent. The extracted RNA was quantified on the Nanodrop ND-1000 spectrophotometer (Thermo Fisher Scientific); 1 µg of each sample was reverse-transcribed with Evo M-MLV RT Reaction Mix (Cat#AG11728, Accurate). Quantitative PCR was run on an iCycler Real-Time Detection System (Bio-Rad) using SYBR Green Supermix (Cat#AG11701, Accurate) and gene-specific primer pairs. For semiquantitative RNA quantification, transcript levels were normalised to *Actb* or *Mtco2* as indicated. Circular versus linear RNA abundance was discriminated with divergent and convergent primers, respectively; *Actb* mRNA served as the endogenous reference. For absolute RNA quantification, a standard curve was generated using serial dilutions of in vitro transcribed back-splice junction of circular BRAKE RNA. Equivalent molecules per cell were then extrapolated from this standard curve and the corresponding threshold cycle (Ct) values, under the assumption that each cell contains 20 pg of total RNA. All qPCR primers, designed and synthesized by Synbio Technologies (Suzhou, China), were provided in Supplementary Table S1 within the Supplementary Information.

### Actinomycin D treatment

To evaluate the stability of circRNA BRAKE, cells were treated with the transcriptional inhibitor actinomycin D (Cat#HY-17559, MCE) to block de novo RNA synthesis, allowing measurement of circRNA BRAKE decay over time. Briefly, cells were plated in 6-well plates and grown to 70–80% confluence. Complete medium was then replaced with fresh medium containing 5 µg/mL actinomycin D; parallel wells received vehicle (DMSO ≤ 0.1%). At 0, 6, 12, 18, and 24 h after drug addition, cultures were lysed, and total RNA was isolated with TRIzol reagent. Convergent primers were used to detect the linear BRAKE mRNA as a control to compare degradation kinetics between circRNA BRAKE and its linear counterpart.

### RNase R treatment

To assess circRNA BRAKE stability, 2 μg of cellular RNA was combined with 1 μl 10×Reaction Buffer and 0.4 μl RNase R (Cat#RNR07250, Epicentre) or an equal volume of RNase-free water. After 15 min at 37 °C, reactions were terminated by heating to 70 °C for 10 min. Residual levels of circular and linear BRAKE transcripts were subsequently quantified by qPCR.

### Oligo (dT) primers

To generate cDNA, total RNA underwent reverse transcription primed with either random hexamers or oligo(dT) primers (Cat#11612, Accurate), the choice being dictated by the nature of the RNA template. Oligo(dT) primers were specifically employed for RNA templates with poly(A) tails. The reverse transcription reaction was initiated by gently vortexing the PCR tubes to ensure thorough mixing, followed by brief centrifugation. The reaction was then performed in a standard thermal cycler under the following conditions: 37 °C for 15 min to facilitate reverse transcription of poly(A)-tailed RNA, followed by enzyme inactivation at 85 °C for 5 sec. The synthesized cDNA products were immediately stored at −20 °C for subsequent analyses. qPCR was conducted using the synthesized cDNA as a template to amplify and quantify target genes.

### RNA interference

siRNA delivery: 1 × 10⁵ APCs cells were seeded in 6-well plates and transfected with gene-specific siRNA duplexes (Ribobio) using Lipofectamine RNAiMAX (Cat#13778150, Invitrogen) following the supplier’s protocol. Oligonucleotide sequences, designed and synthesized by Synbio Technologies (Suzhou, China), are given in Supplementary Table S2 within the Supplementary Information. Cells were collected for downstream assays (i) 48–72 h after knockdown, (ii) after a further 24 h challenge with 100 µM palmitate (Cat#P9767, Sigma-Aldrich), or (iii) at the end of adipogenic differentiation. Silencing efficiency was verified by qRT-PCR for RNA and western blot for protein.

Overexpression constructs: DNA fragments encoding full-length circRNA BRAKE (mmu_circRNA_013298) and a 67–134 nt deletion mutant (circRNA BRAKE-mut) were chemically synthesized by Synbio Technologies (Suzhou, China), PCR-amplified, and inserted into the pcD-ciR vector (Geneseed Biotech, China). The recombinant pcD-ciR plasmids were co-transfected with lentiviral packaging plasmids into HEK293 cells (CSTR: 1101HUM-PUMC000010, National Biomedical Cell-Line Resource) to produce lentiviral particles. The viral supernatant was harvested, filtered through a 0.45 μm membrane, and used to infect APCs in the presence of 5 μg/mL polybrene. Stable cell lines were established by puromycin selection (2 μg/mL) at 48–72 h post-infection. In parallel, UCSC-based prediction identified four Alu elements most likely to drive linear BRAKE circularization; wild-type or Alu-deletion variants (single-Alu or all-four-Alu deletions) were synthesized by Synbio Technologies (Suzhou, China) and subcloned into the pcDNA3.1 vector. Each construct was introduced into HEK293 cells with Lipofectamine 2000 (Cat#11668019, Invitrogen) according to the manufacturer’s instructions. RNA species generated from the vectors were validated by in vitro transcription followed by qRT-PCR.

### Western blot analysis

Tissue and cultured adipocytes were lysed with the Total Protein Extraction Kit (Cat#KTP3007, Abbkine). Homogenates were heated at 100 °C for 5 min and centrifuged (14,000 × *g*, 20 min, 4 °C); the lipid-free upper phase was carefully aspirated for downstream assays. Standard western blotting was performed on these extracts. For OXPHOS analysis, samples were incubated at 37 °C instead of boiling to preserve heat-labile respiratory complexes. Proteins were separated on 10% SDS-PAGE, electro-transferred to PVDF membranes, blocked with 5% skim milk for 1 h, and probed overnight at 4 °C with primary antibodies. After washing, HRP-conjugated secondary antibodies were applied for 1 h and signals were detected by ECL. Antibodies used are listed below: ACTB (Cat#3700, CST), OXPHOS antibody cocktail (Cat#110413, Abcam), BIRC6 (Cat#8756 T, CST), AKT (Cat#4691, CST), pAKT (Cat#4060, CST), PPARγ (Cat#2435, CST), ATP5A (Cat#14676, Proteintech), ATP5B (Cat#ab14730, Abcam), HSP90 (Cat#sc-13119, Santa Cruz), DBT (Cat#12451, Proteintech), PCCB (Cat#11139, Proteintech), pS6K (Cat#28735, Proteintech), S6K (Cat#14485, Proteintech), Goat Anti-Rabbit IgG-HRP (Cat#CW0103, Cowin Biotech), Goat Anti-Mouse IgG-HRP (Cat#CW0102, Cowin Biotech). The antibodies and reagents used in this study are detailed in Supplementary Table S3 within the Supplementary Information.

### Northern blotting

Northern analysis was carried out with the Biotin Northern Blot Kit (Cat#R0219, Beyotime). RNA samples and an RNA Ladder (Cat#G3372, Servicebio) were resolved on 15% urea-PAGE for 30 min at 100 V, then electro-transferred for 2 h onto a positively charged nylon membrane (Cat#INYC00010, Merck Millipore). RNA was cross-linked to the membrane by UV (254 nm, 120 mJ /cm^2^, 2 min). After a 30-min pre-hybridization in Hyb buffer (Roche) at 55 °C, digoxigenin-labelled oligonucleotide probes (10 pM) were hybridised overnight at the same temperature. Following a 15-min blocking step, the blot was incubated with streptavidin–HRP for 15 min at room temperature. Signals were developed with BeyoECL Moon working solution and SuperSignal™ West Femto (Thermo Fisher Scientific). Probe sequences, designed and synthesized by Synbio Technologies (Suzhou, China), are listed in Supplementary Table S2 within the Supplementary Information.

### Blue Native PAGE (BN-PAGE)

Protein complexes were extracted using 1% digitonin in 20 mM Tris-HCl (pH 7.4), 0.1 M NaCl, 10% glycerol, and protease inhibitors. Samples were mixed with 5% Coomassie G-250 and separated on 4–16% gradient gels at 4 °C. Cathode buffer contained 0.02% Coomassie G-250. Electrophoresis was performed with a cathode buffer at 100 V until the dye front exited the stacking gel, then 250 V until completion. Complexes were visualized by Coomassie staining.

### Immunoprecipitation

Immunoprecipitation (IP) was performed using treated APCs. Cells were lysed in RIPA buffer (30 min, ice), centrifuged (12,000 × *g*, 4 °C, 20 min), and supernatants collected. For RNA pull-down, lysates were incubated with biotinylated DNA probes (200 nM, Synbio Technologies) targeting circRNA BRAKE (37 °C, overnight), followed by streptavidin magnetic beads (Cat#HY-K0208, MCE). Beads were washed (IP buffer), and bound proteins were analyzed by WB/MS. For in vitro assays, truncated circRNA BRAKE RNAs were transcribed using the TranscriptAid T7 Kit (Cat#K0441, Thermo Fisher) and biotinylated using the RNA 3’ End Biotinylation Kit (Cat#20160, Thermo Fisher). For RIP/CoIP, lysates were precleared with protein A/G beads (Cat#HY-K0202, MCE) and incubated with anti-ATP5A (Cat#14676, Proteintech) or Rabbit IgG (Cat#30000, Proteintech) at 4 °C, overnight. CoIP samples were resolved by SDS-PAGE and probed with anti-ATP5A (Cat#A11217, ABclonal) and anti-ATP5B (Cat#ab14730, Abcam). RNA secondary structures were predicted using the ViennaRNA Package 2.0^63^. RIP samples underwent RNA extraction for circRNA BRAKE qRT-PCR. The sequences of pull-down probes were provided in Supplementary Table S2 within the Supplementary Information.

### Mass spectrometry analysis

MS/MS spectra were searched using MaxQuant v2.0.1.0 with the Andromeda search engine against the UniProt Mus musculus reference proteome (downloaded March 21, 2023; 55,260 entries). Trypsin was specified as the protease, allowing up to two missed cleavages. Carbamidomethylation of cysteine was set as a fixed modification, whereas methionine oxidation and protein N-terminal acetylation were set as variable modifications. The minimum peptide length was 6 amino acids. The first-search precursor mass tolerance was 20 ppm, the main-search precursor tolerance was 4.5 ppm, and the fragment-ion tolerance was 20 ppm. Peptide-spectrum match, site, and protein false discovery rates were controlled at 1%. For protein quantification, MaxQuant calculated both Intensity and iBAQ values, and missing values were not processed. Proteins were considered identified when at least 1 unique peptide was detected.

### CCK8

Cells were plated at 3 × 10³ per well in 96-well plates (triplicate wells per group). After overnight cell culture, 10 μL CCK-8 reagent (Cat#CK04, DOJINDO) was added to each well and incubated in the dark for 1 h inside the culture incubator. This procedure was repeated every 24 h at the same clock time for 72 h, and absorbance at 450 nm was read immediately on a Tecan Spark10M microplate reader.

### ATP production

ATP levels were determined with the Enhanced ATP Assay Kit (Cat#S0027, Beyotime). Adipose tissue (50 mg) or cell monolayers (6-well format) were disrupted in 200 µL ATP lysis buffer; tissue was processed with a cryo-grinder, and cells were lysed on the plate. Lysates were cleared by centrifugation (300 × *g*, 5 min, 4 °C). 100 µL of ATP reaction reagent was dispensed into each well, incubated 5 min at room temperature, and 20 µL of supernatant or standard was added. Luminescence (RLU) was read on a Tecan Spark10M luminometer and normalised to mtDNA/nDNA measured with qPCR.

### Extracellular acidification and oxygen consumption rate assays

Concurrent assessment of glycolytic flux and mitochondrial respiration was achieved by real-time recording of oxygen consumption (OCR) and extracellular acidification (ECAR) with the Seahorse XF Mito Stress Test Kit (Cat#103015-100, Seahorse Bioscience) and the Seahorse XF Glycolysis Stress Test Kit (Cat#103020-100, Seahorse Bioscience), respectively, following the manufacturer’s validated workflows. For the experimental setup, cell suspensions were plated in specialized 24-well or 96-well XF assay plates (Seahorse Bioscience) and maintained in complete medium (DMEM + 10% FBS) for 12-16 hours, or induced to differentiate after reaching contact inhibition. Before starting the metabolic run, cultures were washed twice and pre-incubated for 1 h at 37 °C in ambient air in assay medium composed of Seahorse XF Base Medium (Cat#103193-100, Seahorse Bioscience) plus 2 mM L-glutamine. Following baseline recording, metabolic perturbations were induced through timed injections: for glycolytic assessment, cells received 2.5 mM glucose followed by 1 μM oligomycin and 10 mM 2-DG; mitochondrial profiling was achieved through sequential administration of 1 μM oligomycin, 0.5 μM FCCP, and 1 μM rotenone/antimycin A mixture. All compounds were provided with the commercial kit unless otherwise specified. Data acquisition and analysis were conducted using the proprietary Seahorse XF Wave software, with results normalized to pmol O₂/min (OCR) and mpH/min (ECAR).

### L-lactate content measurement (L-LA)

L-lactate content was measured with the L-lactic Acid Assay Kit (Cat#BC2235, Solarbio) following the manufacturer’s protocol. Briefly, cell lysis was performed by 300 W ice-bath sonication (3 s on / 7 s off, total 3 min). After centrifugation at 4 °C, 12,000 × *g* for 10 min, 0.8 mL of supernatant was mixed with 0.15 mL of extractant, re-centrifuged under the same conditions, and the final supernatant was used for L-lactate determination. Absorbance at 570 nm was read on a microplate reader (Tecan Spark10M). Lactate values were expressed per microgram of protein determined by the BCA Protein Assay Kit (Cat#CW0014, Cowin Biotech).

### ELISA

Insulin and adiponectin were quantified with Abclonal Mouse Insulin (Cat#RK09268) and Adiponectin (Cat#RK02574) ELISA kits, respectively. Serum or culture supernatant (100 µL) and serial standards were loaded into microplate wells and incubated 2 h at 37 °C. Biotinylated detector antibody (100 µL) was then applied for 1 h at 37 °C, followed by 30 min with streptavidin–HRP (100 µL). Chromogenic development was initiated with 100 µL TMB substrate, shielded from light for 15–20 min at 37 °C, halted with 50 µL stop solution. Serum FFA levels were quantified according to the instructions of the FFA ELISA kit (Cat#MM-0326, MEIMIAN). Briefly, standards were serially diluted, and samples were diluted 1:5. After incubation at 37 °C for 30 min, plates were washed five times. Horseradish peroxidase-conjugated antibody was added, followed by another incubation and wash cycle. After 10 min of dark incubation at 37 °C with mixed TMB reagents A and B, the chromogenic reaction was terminated by adding stop solution. The absorbance at 450 nm was measured immediately using a microplate reader (Tecan Spark10M).

### Open reading frame (ORF) prediction and verification

Coding capacity of circRNA BRAKE was evaluated with the online Coding Potential Calculator (CPC, https://cpc.gao-lab.org/). After uploading the complete sequence, the platform’s default settings were retained, allowing the support vector machine to integrate six ORF-centric features together with BLASTX homology scores and return a probability estimate of protein-coding potential.

### ATP synthase activity assay

The activity of mitochondrial respiratory chain ATP synthase was determined using the ATP synthase Activity Assay Kit (Cat#BC1445, Solarbio). Briefly, cells (5×10^6) were homogenized in extraction buffer, followed by centrifugation. The pellet was ultrasonically lysed in Reagent 1. The enzymatic reaction was initiated by adding Reagents 2 and 3, incubated at 37 °C for 30 min, and then terminated with Reagent 4. The optical density at 660 nm was measured immediately using a microplate reader (Tecan Spark10M).

### Statistical analysis

Data evaluation was carried out with GraphPad Prism 8 (GraphPad Software). Values are presented as mean ± SEM. Pairwise contrasts were assessed by unpaired, two-tailed Student’s *t*-test; three or more means were compared through one-way ANOVA coupled with Tukey’s multiple comparisons test. For experiments involving two independent factors, such as genotype and treatment or genotype and subcellular fraction, data were analyzed using two-way ANOVA followed by Šídák’s multiple comparisons test. For time-course experiments, including metabolic cage analyses and tolerance tests, repeated-measures two-way ANOVA or mixed-effects analysis was used as appropriate, followed by Šídák’s multiple comparisons test. Phase-specific metabolic cage parameters during light and dark phases were analyzed using two-way ANOVA followed by Šídák’s multiple comparisons test. Associations among continuous variables were first inspected by Pearson correlation after Kolmogorov–Smirnov confirmation of normality; otherwise, Spearman's rank correlation procedures were substituted. Sample sizes were chosen without prior power analysis. A threshold of *P* < 0.05 was used to define statistical significance.

### Figure preparation

Schematic diagrams in Figs. 2C, 3A, and 5Q were assembled using Microsoft PowerPoint. The mouse illustrations were hand-drawn by the authors using the digital painting software Huashijie. Selected graphical elements used in the schematics, such as adipose tissue illustrations, were sourced from the FigDraw platform under license.

## Supplementary information

Supplementary Information

Transparent Peer Review file

## Source data

Source data

## Data Availability

The circRNA microarray and RNA-seq data generated in this study have been deposited in the Gene Expression Omnibus (GEO) database under accession code GSE338250 and GSE338175. The mass spectrometry proteomics data have been deposited to the ProteomeXchange under accession code PXD080904. All other data supporting the findings of this study are available within the paper and its Supplementary Information. Source data are provided with this paper.
