# Supplementary material for: Mitochondria-localized circRNA BRAKE restrains adipogenesis under nutrient overload by disrupting ATP synthase assembly in mice
Source: Nat Commun. 2026 Oct 13;17:11577. doi: 10.1038/s41467-026-78378-z (PMC13752303; doi:10.1038/s41467-026-78378-z)
Supplement: Supplementary file 1 [file 41467_2026_78378_MOESM1_ESM.pdf]

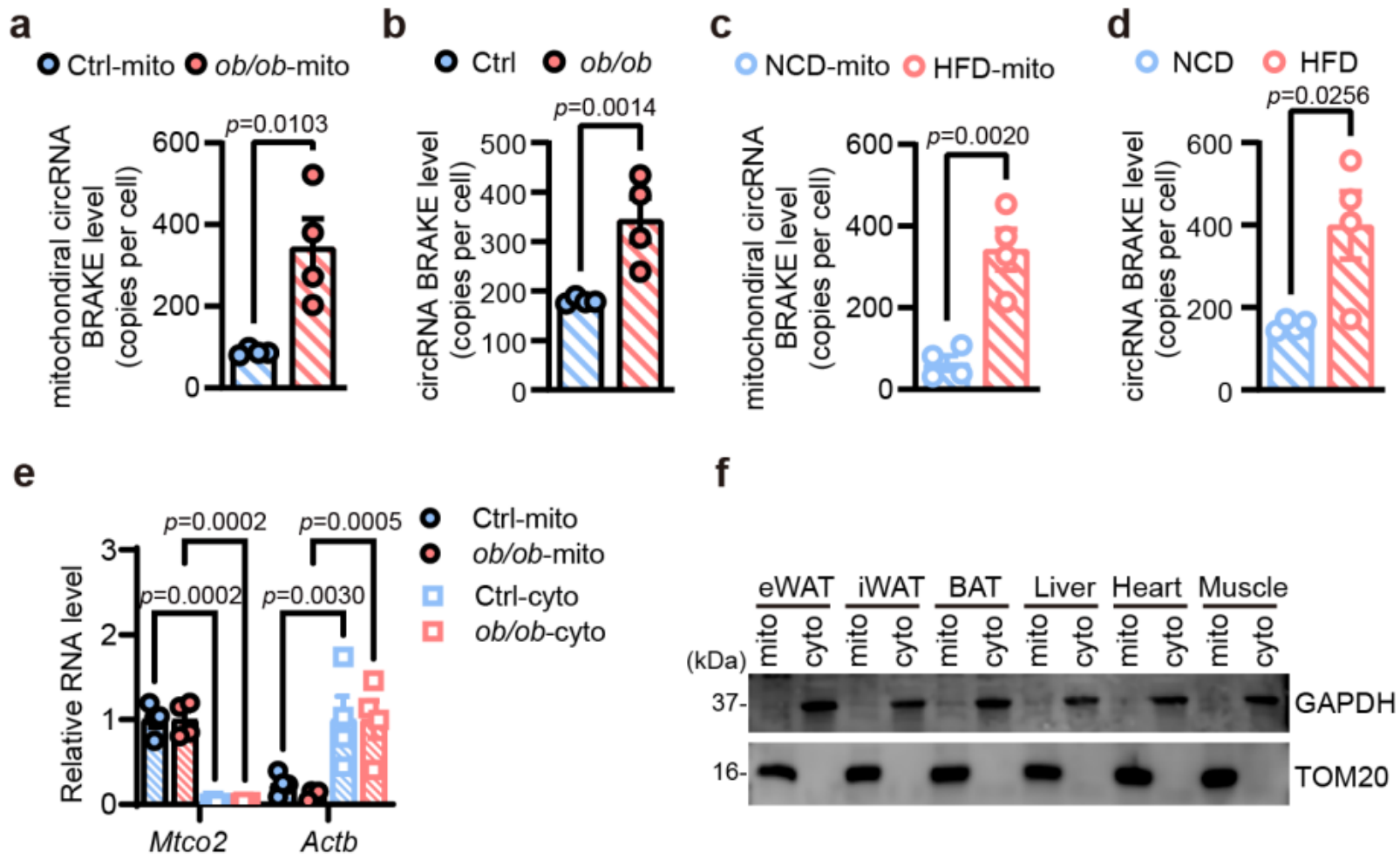

Figure S1. Mitochondrial isolation efficiency of adipose samples from ob/ob mice and control mice.

A Absolute qRT-PCR validation of circRNA BRAKE abundance in mitochondrial fractions of APCs in eWAT from *ob/ob* and control mice (*n* = 4). **B** Absolute qRT-PCR validation of circRNA abundance in APCs from *ob/ob* and control mice (*n* = 4). **C** Absolute qRT-PCR validation of circRNA BRAKE abundance in mitochondrial fractions of APCs in eWAT from HFD and NCD mice (*n* = 4). **D** Absolute qRT-PCR validation of circRNA abundance in APCs from NCD and HFD mice (*n* = 4). **E** qRT-PCR was used to measure *Actb* and *Mtco2* mRNA levels in cytosolic and mitochondrial fractions of eWAT (*n* = 4), serving as markers for the cytosolic and mitochondrial compartments, respectively. **F I**mmunoblotting analysis of TOM20 and GAPDH in mitochondrial and cytosolic fractions isolated from the indicated metabolic tissues. TOM20 was used as a mitochondrial marker and GAPDH as a cytosolic marker. Each data point represents an independent biological replicate. All quantitative data are expressed as mean ± SEM. Statistical significance was analyzed by two-way repeated-measures ANOVA followed by Šídák's multiple comparisons test or two-tailed Student's *t*-test. compared with indicated groups. ns, not significant. Source data are provided as a Source Data file.

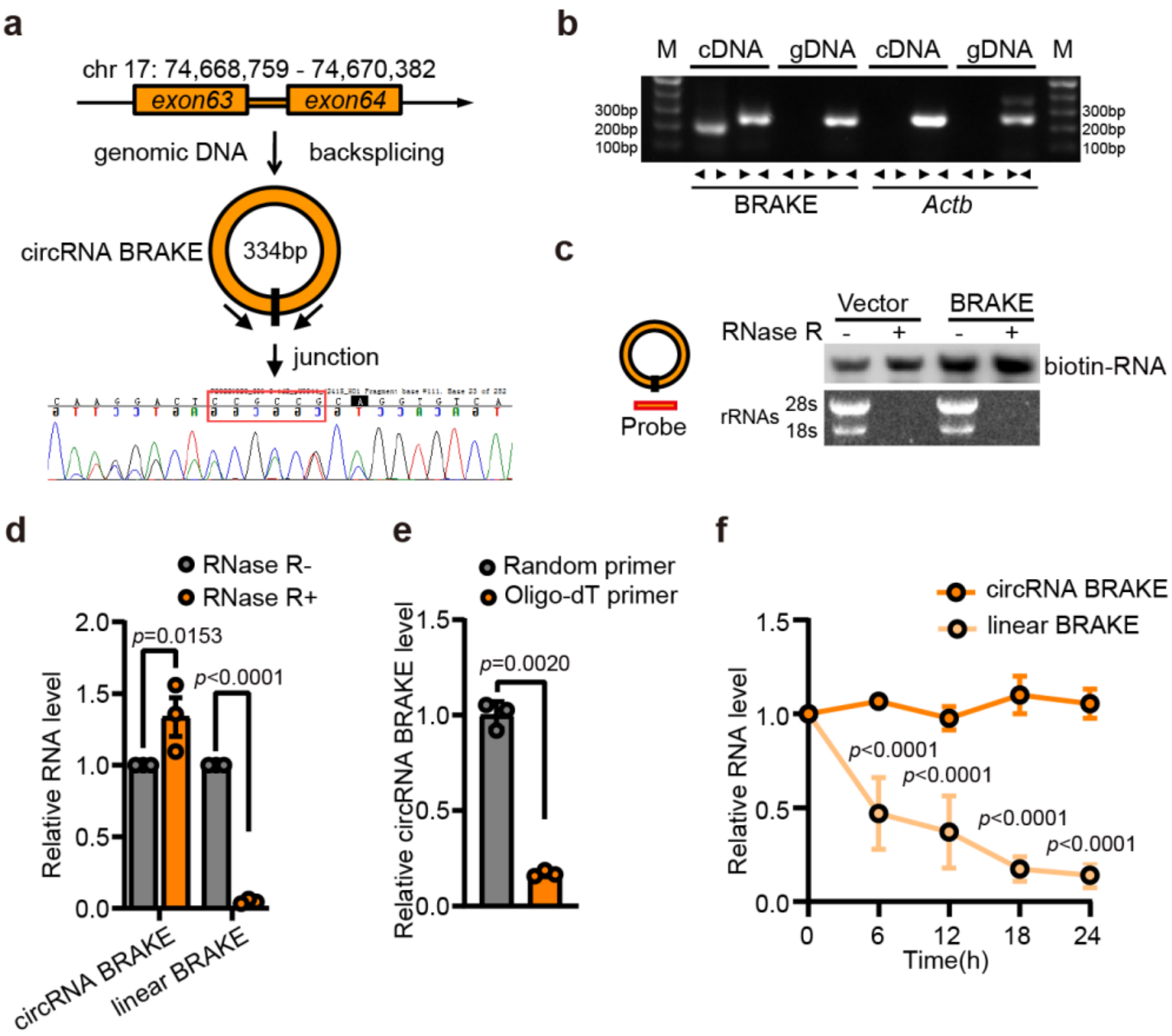

Figure S2. Characterization of circRNA BRAKE in mouse adipocytes. A Schematic illustration of circRNA BRAKE biogenesis. Sanger sequencing confirmed the circular RNA junction. **B** PCR detection of circRNA BRAKE using divergent and convergent primers on DNA templates. Convergent and divergent primers are indicated by inward- and outward-facing arrows, respectively. *Actb* served as a control for linear RNA transcripts. **C** Design of circRNA BRAKE-specific probes (left) and northern blot results (right, *n* = 3). **D** qRT-PCR analysis of circRNA BRAKE and linear BRAKE expression ± RNase R (*n* = 3). **E** Detection of circRNA BRAKE following reverse transcription with random or oligo-dT primers in total RNA from 3T3-L1 cells(*n* = 3). **F** Time-course analysis of circRNA BRAKE and linear BRAKE transcript stability measured by qRT-PCR post-actinomycin D(*n* = 3). Each data point represents an independent biological replicate. All quantitative data are as mean ± SEM. Statistical significance was analyzed by two-way repeated-measures ANOVA followed by Šídák's multiple comparisons test or two-tailed Student's *t*-test. Source data are provided as a Source Data file.

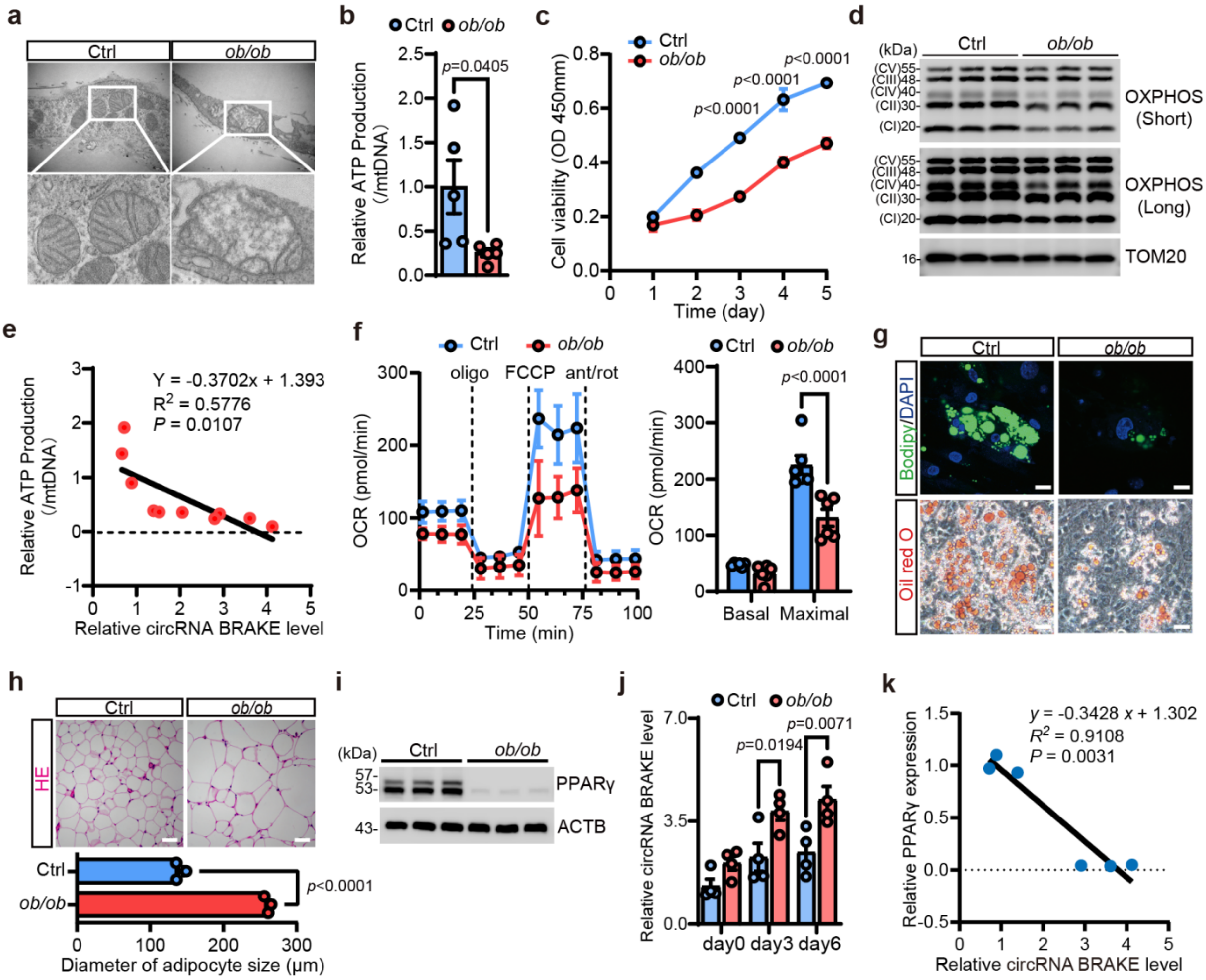

Figure S3. Elevated circRNA BRAKE correlates with mitochondrial and adipogenic dysfunction in obese eWAT. Control and *ob/ob* male mice (sixteen weeks old) were maintained on NCD. **A** Mitochondrial structures in eWAT by EM. White boxes denote magnified areas (n = 3). **B** ATP production in eWAT (n = 5). **C** Cell viability of APCs in eWAT was measured by CCK-8 assay (n = 4). **D** Representative immunoblots of OXPHOS complexes I–V in eWAT (n = 3). **E** Correlation between mitochondrial expression of circRNA BRAKE and ATP production in eWAT (n = 10). Mitochondrial circRNA BRAKE values are the same data as those shown in Fig.1G and are replotted here for association with ATP production. **F** The oxygen consumption rate (OCR) in differentiated adipocytes in eWAT (n = 6). **G** Representative BODIPY and Oil Red O staining images in differentiated adipocytes of eWAT (n = 3). Scale bar: BODIPY, 20 μm; Oil Red O, 40 μm. **H** Representative hematoxylin and eosin images in eWAT, with quantification of adipocyte size (n = 3). Scale bar, 50 μm. **I** Immunoblots of PPARγ protein in differentiated adipocytes of eWAT (n = 3). **J** Quantitative RT-PCR validation of circRNA abundance in mitochondrial fractions from APCs in eWAT from *ob/ob* and control mice (n = 4). **K** Correlation between mitochondrial expression of circRNA BRAKE and protein levels of PPARγ in eWAT (n = 6). Mitochondrial circRNA BRAKE values are the same data as those shown in Fig.1G and are replotted here for association with PPARγ levels. Each data point represents an independent biological replicate. All quantitative data are as mean ± SEM. Pearson correlation analysis was used to evaluate associations between variables. Statistical significance was analyzed by two-way ANOVA Šídák' s multiple comparisons test or two-tailed Student's *t*-test. Source data are provided as a Source Data file.

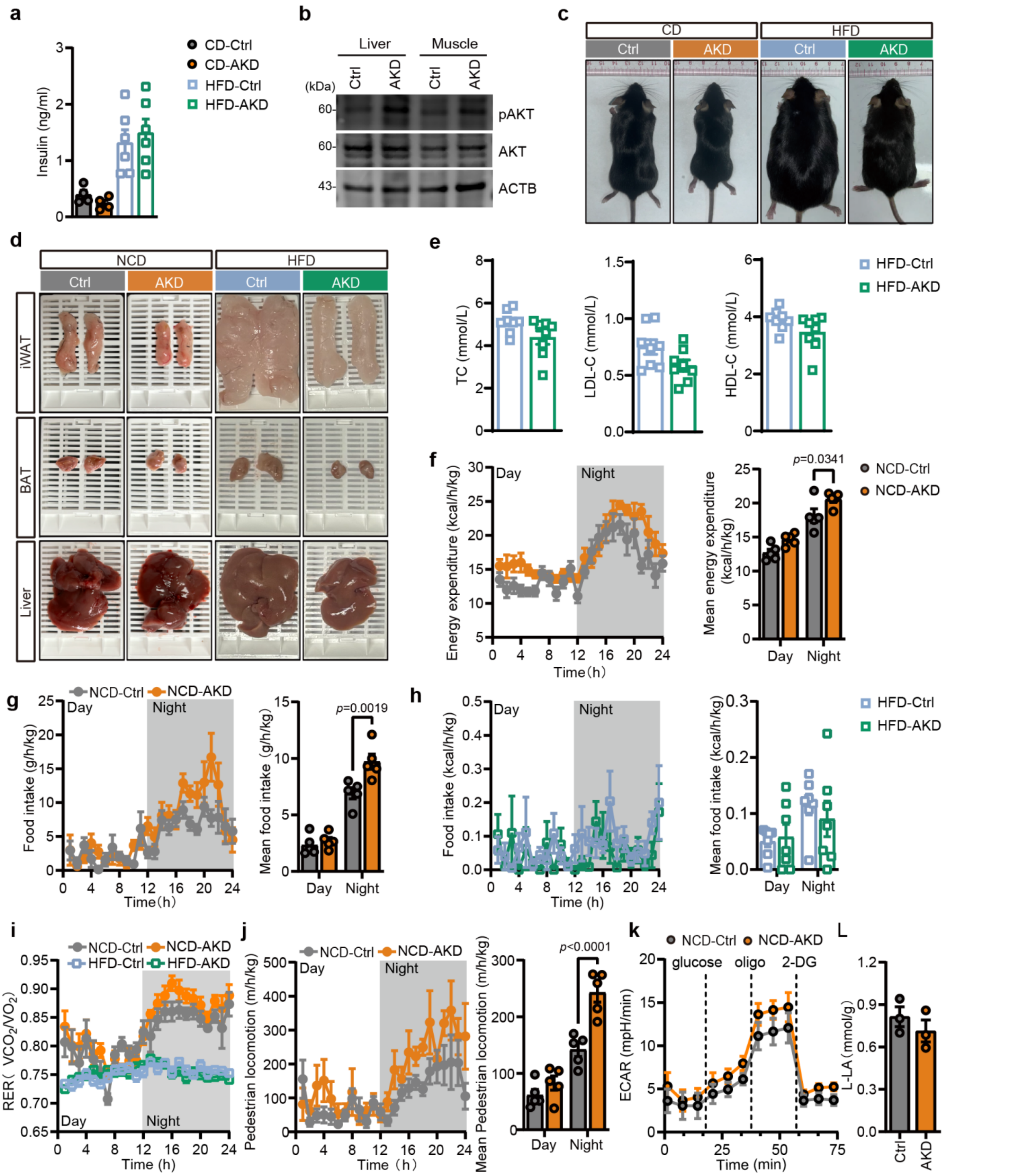

Figure S4. Adipocyte-specific circRNA BRAKE knockdown improves metabolic homeostasis and reduces adiposity. 24-week-old AKD male and control mice were maintained on NCD or HFD. **A** Fasting plasma insulin concentrations in NCD- (*n* = 4) and HFD- (*n* = 6) fed mice. **B** Representative western blots for total AKT and pAKT in liver and muscle 15 min after insulin injection (2 U/kg) (*n* = 3). **C** Gross images of in NCD- (*n* = 5) and HFD- (*n* = 6) fed mice. **D** iWAT, BAT and Liver morphology in NCD (*n* = 5) and HFD (*n* = 6) groups. **E** Plasma TC, LDL-C, and HDL-C measurements after overnight fasting in mice under HFD (*n* = 8). **F** Energy expenditure in mice under NCD (*n* = 5). **G** Food intake of mice under NCD (*n* = 5). **H** Food intake of mice under HFD (*n* = 7). **I** Respiratory exchange rate (RER, VCO_2/VO_2) in NCD- (*n* = 5) and HFD- (*n* = 7) fed mice. **J** Pedestrian locomotion of mice under NCD (*n* = 5). **K** The extracellular acidification rate (ECAR) in APCs from mice after differentiation (*n* = 6). **L** Levels of L-lactate (L-LA) in differentiated adipocytes (*n* = 3). Each data point represents an independent biological replicate. All quantitative data

are as mean ± SEM. Statistical significance was analyzed by two-tailed Student's *t*-test. Source data are provided as a Source Data file.

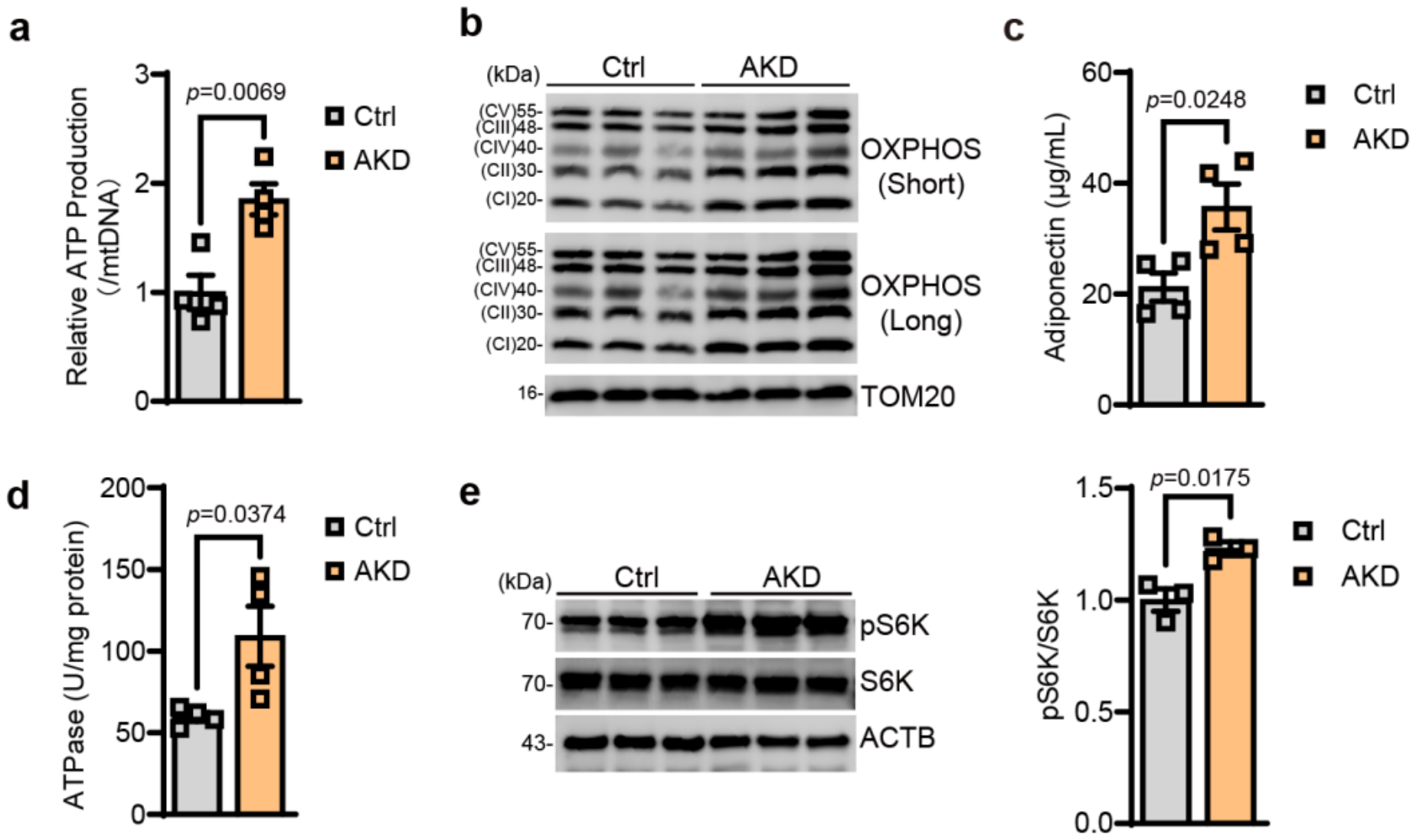

Figure S5. Adipocyte-specific circRNA BRAKE knockdown improves mitochondrial energy metabolism and adiponectin secretion in eWAT. 24-week-old AKD male and control mice were maintained on NCD. **A** ATP production in eWAT (n = 4). **B** ATP synthase activity in eWAT (n = 4). **C** Representative western blots for OXPHOS complexes I–V in eWAT (n = 3). **D** Representative western blots for total S6K and pS6K in eWAT, with quantification of pS6K/S6K ratio (n = 3). **E** Adiponectin levels in serum (n = 4). Each data point represents an independent biological replicate. All quantitative data are expressed as mean ± SEM. Statistical significance was analyzed by two-tailed Student's t-test. Source data are provided as a Source Data file.

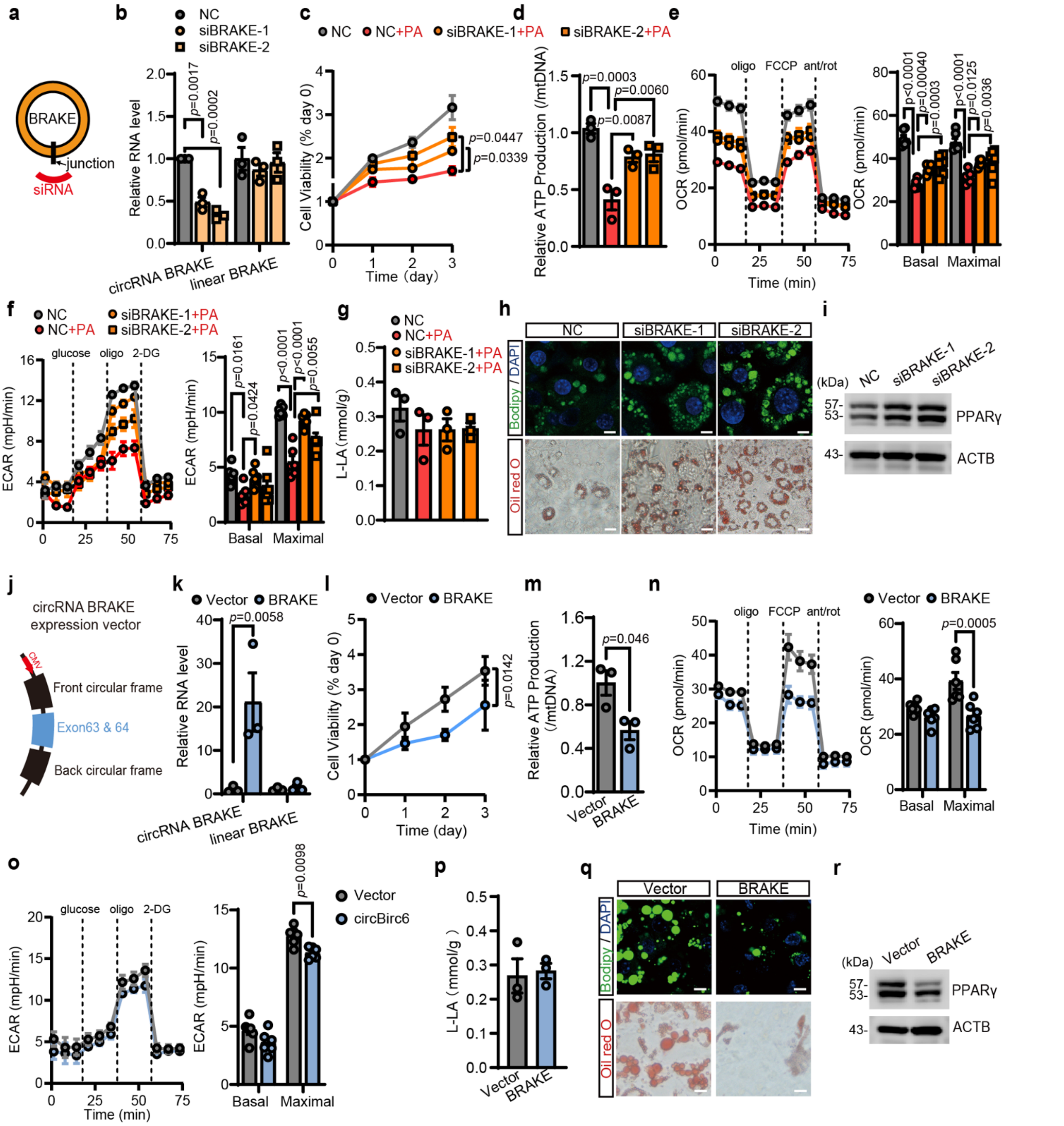

Figure S6. CircRNA BRAKE links mitochondrial dysfunction to impaired adipogenesis in pre-adipocytes. Experiments were performed on APCs. **A** Design of siRNAs specifically targeting the junction of circRNA BRAKE. **B** qRT-PCR measurement of circRNA BRAKE and linear BRAKE in transfected cells transfected with con-siRNA (NC) or circRNA BRAKE-siRNA (BRAKE) (n = 3). **C** Cell viability after transfection, with or without PA treatment, determined by CCK-8 assay (n = 3). **D** Measurement of ATP production in cells following siRNA transfection and PA treatment (n = 3). **E–F** Oxygen consumption rate (OCR) along with extracellular acidification rate (ECAR) with basal and maximal respiration quantification after siRNA transfection and PA treatment (n = 6). **G** Intracellular L-lactate (L-LA) levels following siRNA transfection and PA treatment (n = 3). **H** Representative BODIPY and Oil Red O images of differentiated cells after siRNA transfection (n = 3). Scale bar: BODIPY, 10 μm; Oil Red O, 20 μm. **I** Representative BODIPY and Oil Red O images of differentiated cells after siRNA transfection (n = 3). **J** Schematic of circRNA BRAKE overexpression vectors. **K** qRT-PCR measurement of circRNA BRAKE and linear BRAKE expression in transfected cells (Vector or circRNA BRAKE) (n = 3). **L** Cell viability after vector or circRNA BRAKE overexpression, assessed by

CCK-8 assay (*n* = 3). **M** Quantification of ATP production following circRNA BRAKE overexpression (*n* = 3). **N–O** OCR and ECAR with basal and maximal respiration quantification after vector or circRNA BRAKE overexpression (*n* = 6). **P** L-LA levels following overexpression of circRNA BRAKE (*n* = 3). **Q** Representative BODIPY and Oil Red O images following overexpression of circRNA BRAKE (*n* = 3). Scale bar: BODIPY, 10 μm; Oil Red O, 20 μm. **R** Immunoblot assessment of PPARγ after circRNA BRAKE overexpression (*n* = 3). Each data point represents an independent biological replicate. All quantitative data are as mean ± SEM. Statistical significance was analyzed by two-way ANOVA Šídák' s multiple comparisons test or two-tailed Student's *t*-test. Source data are provided as a Source Data file.

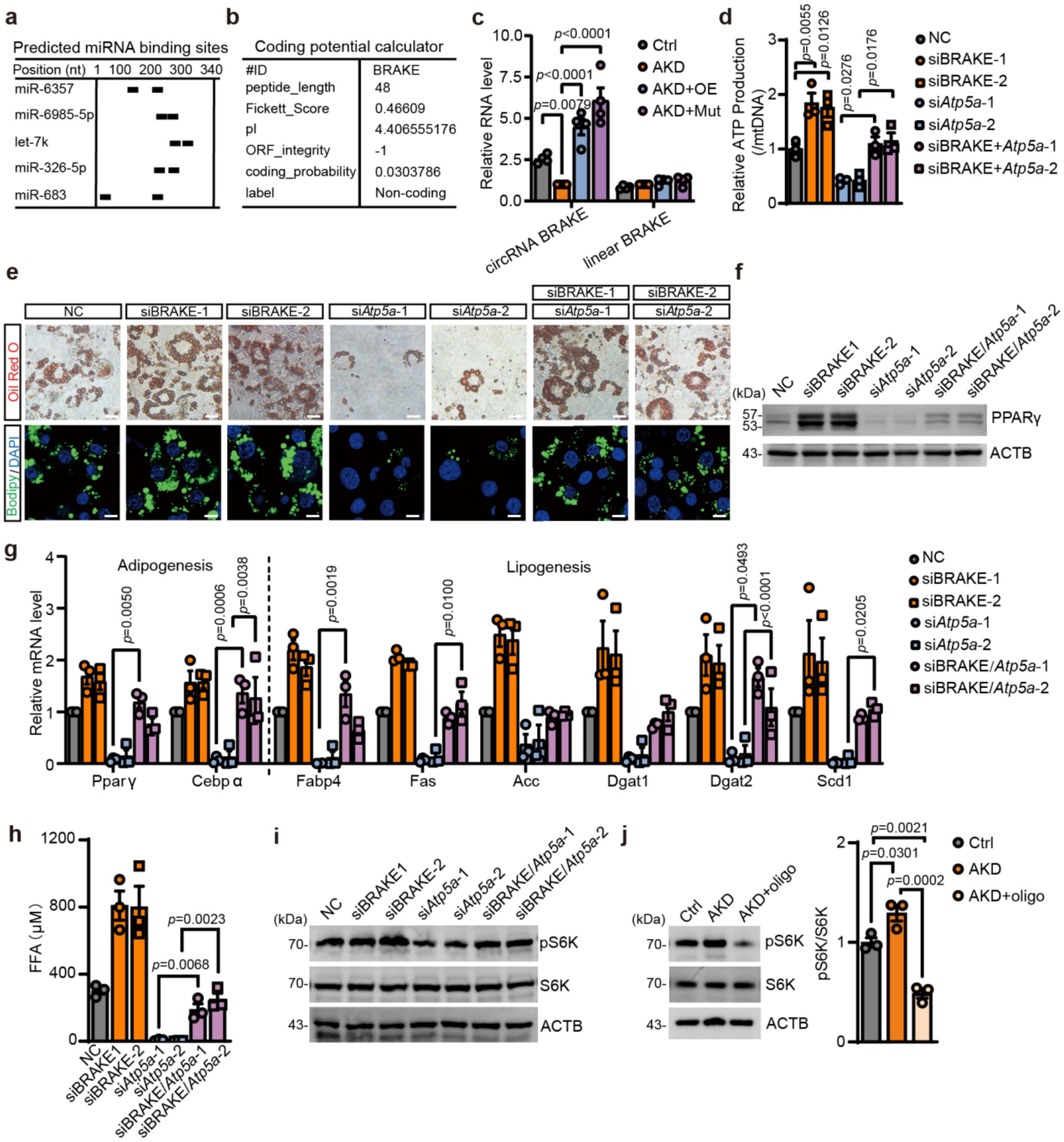

Figure S7. CircRNA BRAKE impairs ATP production and adipogenesis in pre-adipocytes via ATP5A. Experiments were performed on APCs. **A** In silico identification of potential miRNA target sites within circRNA BRAKE through TargetScan and miRANDA. **B** Open reading frame (ORF) prediction of circRNA BRAKE by Coding Potential Calculator (CPC). **C** Quantification of circRNA BRAKE and linear BRAKE by qRT-PCR in cells following transfection with con-siRNA, circRNA BRAKE-siRNA and *Atp5a*-siRNA (*n* = 3). **D** ATP production was assessed in transfected differentiated cells (control, circRNA BRAKE, or *Atp5a* siRNAs; *n* = 3). **E** Representative BODIPY and Oil Red O images of differentiated cells after transfection (*n* = 3). Scale bar: 10 μm. **F** Immunoblot of PPARγ protein in differentiated cells after transfection (*n* = 3). **G** qRT-PCR of adipogenic and lipogenic gene in differentiated cells after transfection (*n* = 3). **H** FFA levels in culture medium of differentiated cells after transfection (*n* = 3). **I** Immunoblot assessment of pS6K and total S6K protein in differentiated cells after transfection (*n* = 3). **J** Immunoblot of pS6K and total S6K in differentiated adipocytes of AKD mice and control mice after oligomycin (oligo) treatment, with quantification of pS6K/S6K ratio (*n* = 3). Each data point represents an independent biological replicate. All quantitative data are as mean ± SEM. Statistical significance was analyzed by two-way ANOVA Šídák' s multiple comparisons test. Source data are provided as a Source Data file.

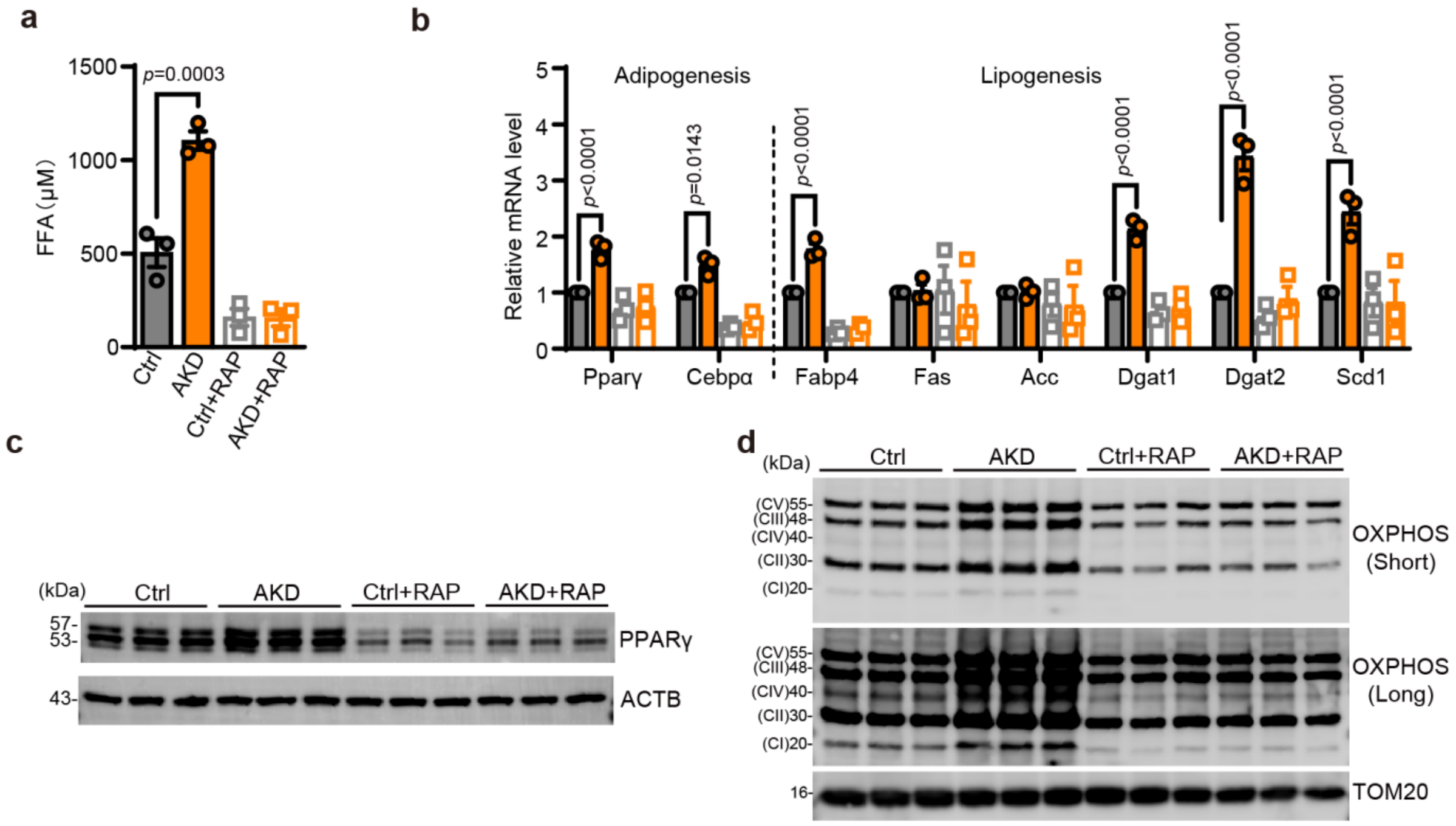

Figure S8. CircRNA BRAKE impairs adipogenesis in pre-adipocytes via mTORC1 pathway.

A FFA levels in culture medium of differentiated cells after rapamycin (RAP) treatment (*n* = 3). **B** qRT-PCR of adipogenic and lipogenic gene in differentiated adipocytes after rapamycin treatment (*n* = 3). **C** Immunoblot of PPARγ protein in differentiated adipocytes after rapamycin treatment (*n* = 3). **D** Representative western blots for OXPHOS complexes I–V in differentiated adipocytes after rapamycin treatment (*n* = 3). Each data point represents an independent biological replicate. All quantitative data are as mean ± SEM. Statistical significance was analyzed by two-way ANOVA Šídák’ s multiple comparisons test. Source data are provided as a Source Data file.

Score	Expect	Identities	Gaps	Strand	Frame
329 bits(364)	2e-94()	272/332(82%)	0/332(0%)	Plus/Plus	

```
Query  1    CCGCAGGTGTCAAATTCCCATAACCCCACATCAGCAGAAGAACAGCAGCTGTACTGGGCC  60
            || ||||||||||  || |||||||| |||||| |||||||||| ||| | || ||||||
Sbjct  520  CCACAGGTGTCAAGCTCTCATAACCCTACATCAACAGAAGAACAACAGTTATATTGGGCC  579

Query  61   AAAGGGACTGGCTTTGGCACAGGTTCCACGGCATCAGGATGGGACGTGGAACAAGCCTTA  120
            ||||||||||||||||| ||||| || || || || || ||||| |||||||||||||||
Sbjct  580  AAAGGGACTGGCTTTGGAACAGGCTCTACAGCTTCTGGGTGGGATGTGGAACAAGCCTTA  639

Query  121  ACTAAACAGAGGCTAGAAGAGGAGCATGTGACCTGCCTTTTGCAGGTCCTTGCGAGTTAC  180
            ||||| || ||||| |||||||| ||||| ||||||||| ||||||| ||||| ||||||
Sbjct  640  ACTAAGCAAAGGCTGGAAGAGGAACATGTTACCTGCCTTCTGCAGGTTCTTGCCAGTTAC  699

Query  181  ATAAACCCCATGAGTGGTGCTGTCAATGGGGAAGCCCAGGCGTCTCCCGAGAGCCGCGCC  240
            ||||| ||| | ||| |||| || ||||| ||||| ||| | ||||  ||||   | |  
Sbjct  700  ATAAATCCCGTCAGTAGTGCGGTAAATGGAGAAGCTCAGTCATCTCATGAGACTAGAGGG  759

Query  241  CAGAACAGCAGTGCTCTGCCCTCGGTGCTTCTGGAGCTCCTCAGTCAGTCCTGCCTCATC  300
            |||||||| | ||| || || || || ||||| ||||| |||||||||||||||||||||
Sbjct  760  CAGAACAGTAATGCCCTTCCTTCTGTACTTCTCGAGCTTCTCAGTCAGTCCTGCCTCATC  819

Query  301  CCAGCCATGTCCTCCTACCTGCGCAACGACTC  332
            ||||||||||| || || || || || || ||
Sbjct  820  CCAGCCATGTCATCTTATCTACGAAATGATTC  851
```

Figure S9. High conservation of circRNA BRAKE between humans and mice.

Sequence homology between human circRNA BRAKE (hsa_circ_0005544) and mouse circRNA BRAKE (mmu_circRNA_013298) by pairwise alignment (NCBI Blast, blastn suite-2sequences).

Supplementary Table S1. Primer sequences used for real-time PCR analysis

Gene	Sequence (5'-3')	
	Forward primer	Reverse primer
mmu_circRNA_016016	GGACAGATACAGAAGGGGGC	ACGACTCTGAAAACATCCCATCT
mmu_circRNA_013298	CTCCGCCGCAGGTGTCAAA	TCCGCCGCAGGTGTCAAAT
mmu_circRNA_33769	AAGTTTGTGGAGATCCCTGGC	AAAGTCTGTTGTCAGCCGGT
mmu_circRNA_24900	CCTCTTGCGCGTTCCAGTTC	TCTCCGGTAAGGCTTGTTGC
mmu_circRNA_39273	GAGCGGATGCTGTTAGACCT	CCATGCCAGCCTGTCTTTTG
mmu_circRNA_32330	GTGGCAGGGGAGATTCGTTT	TGGGTTGAGGAACTGCTTTTG
mmu_circRNA_007066	CCAACCGGAGGTGACAGAT	CTCCCAACCGGAGGTGACA
mmu_circRNA_43451	AGGCTACGAACCCTGACCT	TCTCGGGAACCAAACCTACT
mmu_circRNA_38943	TTGTCCGAGCTGTATCCGTG	TCCTGAACAGAAATGCCGTCT
mmu_circRNA_33256	CCGGTTTGGGAACAAAGCCT	CTGAGAGCGCTTCCATTTGC
hsa_circ_0005544	TGCCCTTCCTTCTGTACTTC	TTACAACTGGAGCACTCACC
linear BRAKE	CACAGGTTCCACGGCATCAG	CACAGGTTCCACGGCATCAG
Adipoq-cre	ACGGACAGAAGCATTTTCCA	GGATGTGCCATGTGAGTCTG
Adipoq-Ref	CTAGGCCACAGAATTGAAAGATCT	GTAGGTGGAAATTCTAGCATCATCC
Flox-5'arm	CAGGGTAGTTTGGGTTTGTACTTCC	CACGCATGCACACACACACAC
Flox-3'arm	GGAATGATAGAAATTCCACTGGAGAG	TCCTTTGCAGTTTCCTGAGCAC
Alu2-4-KO	ACTACAGCTCAGGTCTTCAGTGCTGT	TCCTTTGCAGTTTCCTGAGCAC
Pparγ	CGGAAGCCCTTTGGTGACTT	CCTCGATGGGCTTCACGTTC
Cebpα	CCCTTGCTTTTTGCACCTCC	GCTTTCTGGTCTGACTGGGG
Adiponectin	CTCCTGCTTTGGTCCCTC	GCCAGTGCTGCCGTCATA
Leptin	CACAGTCTGGAGCGAAGG	CACAATCTGGGAACAAGC
Resistin	TCCTTGTCCCTGAACTGC	ACGAATGTCCCACGAGCC
Fabp4	TCACCTGGAAGACAGCTCCT	AATCCCCATTTACGCTGATG
Fas	GGGTCTATGCCACGATTC	GTGTCCCATGTTGGATTTG
Acc	GAGAGGGGTCAAGTCCTTCC	ACATCCACTTCCACACACGA
Dgat1	ATCTGAGGTGCCATCGTC	ATGCCATACTTGATAAGGTTCT
Dgat2	TCAACCGAGACACCATAGAC	CCTCAAAGATCACCTGCTT
Scd1	CTGACCTGAAAGCCGAGAAG	GCGTTGAGCACCAGAGTGTA

Supplementary Table S2. The sequences of oligonucleotide probes

Biotin-labeled oligo probes for Pull-down	
Name	Sequence (5'-3')
Sense	Biotin-AATTTGACACCTGCGGCGGAGTCGTTGCGCAG
Antisense	Biotin-CTGCGCAACGACTCCGCCGCAGGTGTCAAATT
1-67	Biotin-CCGCAGGTGTCAAATTCCCATAACCCCACATCAGCAGAAGAACAGCAGCTGTACTGGGCCAAAGGGA
1-134	Biotin-CCGCAGGTGTCAAATTCCCATAACCCCACATCAGCAGAAGAACAGCAGCTGTACTGGGCCAAAGGGACTGGCTTTGGCACAGGTTCCACGGCATCAGGATGGGACGTGGAACAAGCCTTAACTAAACAGAGGCTA
1-257	Biotin-CCGCAGGTGTCAAATTCCCATAACCCCACATCAGCAGAAGAACAGCAGCTGTACTGGGCCAAAGGGACTGGCTTTGGCACAGGTTCCACGGCATCAGGATGGGACGTGGAACAAGCCTTAACTAAACAGAGGCTAGAAGAGGAGCATGTGACCTGCCTTTTGCAGGTCCTTGCGAGTTACATAAACCCCATGAGTGGTGCTGTCAATGGGGAAGCCCAGGCGTCTCCCGAGAGCCGCGCCCAGAACAGCAG
1-306	Biotin-CCGCAGGTGTCAAATTCCCATAACCCCACATCAGCAGAAGAACAGCAGCTGTACTGGGCCAAAGGGACTGGCTTTGGCACAGGTTCCACGGCATCAGGATGGGACGTGGAACAAGCCTTAACTAAACAGAGGCTAGAAGAGGAGCATGTGACCTGCCTTTTGCAGGTCCTTGCGAGTTACATAAACCCCATGAGTGGTGCTGTCAATGGGGAAGCCCAGGCGTCTCCCGAGAGCCGCGCCCAGAACAGCAGTGCTCTGCCCTCGGTGCTTCTGGAGCTCCTCAGTCAGTCCTGCCTCATCCCAGCC
135-334	Biotin-GAAGAGGAGCATGTGACCTGCCTTTTGCAGGTCCTTGCGAGTTACATAAACCCCATGAGTGGTGCTGTCAATGGGGAAGCCCAGGCGTCTCCCGAGAGCCGCGCCCAGAACAGCAGTGCTCTGCCCTCGGTGCTTCTGGAGCTCCTCAGTCAGTCCTGCCTCATCCCAGCCATGTCCTCCTACCTGCGCAACGACTCCG
307-334	Biotin-ATGTCCTCCTACCTGCGCAACGACTCCG
67-334	Biotin-ACTGGCTTTGGCACAGGTTCCACGGCATCAGGATGGGACGTGGAACAAGCCTTAACTAAACAGAGGCTAGAAGAGGAGCATGTGACCTGCCTTTTGCAGGTCCTTGCGAGTTACATAAACCCCATGAGTGGTGCTGTCAATGGGGAAGCCCAGGCGTCTCCCGAGAGCCGCGCCCAGAACAGCAGTGCTCTGCCCTCGGTGCTTCTGGAGCTCCTCAGTCAGTCCTGCCTCATCCCAGCC
DIG-labeled oligo probe for FISH	
Name	Sequence (5'-3')
FISH probe	DIG-TGCTGAGGCGGCGTCCAC-DIG
Biotin-labeled oligo probes for Northern blot	
Name	Sequence (5'-3')
Probe	Biotin-TGCTGAGGCGGCGTCCAC
siRNA for RNA interference	
Name	Sequence (5'-3')
sicircRNA BRAKE-1	CTCCGCCGCAGGTGTCAAA
sicircRNA BRAKE-2	TCCGCCGCAGGTGTCAAAT
si*Atp5a*-1	Sense GCCCUCGGUAAUGCUAUUGAUTT<br>Antisense AUCAAUAGCAUUACCGAGGGCTT
si*Atp5a*-2	Sense GCCAAGAUGAACGAUUCCUUUTT<br>Antisense AAAGGAAUCGUUCAUCUUGGCTT

Supplementary Table S3. Materials and experimental systems

Reagent or Resource	Source	Identifier
Experimental Models		
B6/JGpt-*Lep*^{em1Cd25}/Gpt	GemPharmatech	Strain T001461A
C57BL/6J	GemPharmatech	Strain N000013
B6;FVB-Tg(Adipoq-cre)/Nju	The Jackson Laboratory	Strain 028020
B6/JGpt- Alu2-4 em1Cflox/Gpt	GemPharmatech	N/A
3T3-L1	National Biomedical Cell-Line Resource (NSTI-BMCR)	CSTR 16607.09.1101MOU-PUMC000155
HEK293	National Biomedical Cell-Line Resource (NSTI-BMCR)	CSTR 1101HUM-PUMC000010
Biological Samples		
Omental adipose tissue	Sun Yat-sen Memorial Hospital, Sun Yat-sen University	N/A
Antibodises		
Mouse-anti-β-Actin (1:10,000)	Cell Signaling Technology	Cat#3700
Mouse-anti-OXPHOS (1:250)	Abcam	Cat#ab110413
Rabbit-anti-AKT (1:1000)	Cell Signaling Technology	Cat#4691
Rabbit-anti-pAKT (1:1000)	Cell Signaling Technology	Cat#4060
Rabbit-anti-PPARγ (1:1000)	Cell Signaling Technology	Cat#2435
Rabbit-anti-ATP5A (1:1000)	Proteintech	Cat#14676-1-AP
Rabbit-anti-ATP5A (1:1000)	ABclonal	Cat#A11217
Mouse-anti-ATP5B (1:1000)	Abcam	Cat#ab14730
Mouse-anti-HSP90 (1:1000)	Santa Cruz	Cat#sc-13119
Rabbit-anti-DBT (1:5000)	Proteintech	Cat#12451-1-AP
Rabbit-anti-PCCB (1:1000)	Proteintech	Cat#11139-1-AP
Rabbit-anti-pS6K (1:1000)	Proteintech	Cat#28735-1-AP
Rabbit-anti-S6K (1:1000)	Proteintech	Cat#14485-1-AP
Rabbit-anti-BIRC6 (1:1000)	Cell Signaling Technology	Cat#8756T
Rabbit-anti-Tom20 (1:5000)	Proteintech	Cat#11802-1-AP
Rabbit IgG	Proteintech	Cat#30000-0-AP
Digoxigenin-FITC (1:100)	Roche	Cat#11207741910
Cy3 goat anti-rabbit IgG (1:200)	Servicebio	Cat#GB21303
Goat Anti-Rabbit IgG-HRP (1:5000)	Cowin Biotech	Cat#CW0103
Goat Anti-Mouse IgG-HRP (1:5000)	Cowin Biotech	Cat#CW0102
Critical commercial assays		
Modified Oil Red O Staining Kit	Beyotime	Cat#C0158
Mitochondrial Isolation Kit	Thermo Fisher	Cat#89874
RNA Labeling Kit	Arraystar	Cat#074301
Protein Extraction Kit	Abbkine	Cat#KTP3007
Biotin Northern Blot Kit	Beyotime	Cat#R0219
TranscriptAid T7 Kit	Thermo Fisher	Cat#K0441
RNA 3' End Biotinylation Kit	Thermo Fisher	Cat#20160

Reagent or Resource	Source	Identifier
Seahorse XF Cell Mito Stress Test Kit	Seahorse Bioscience	Cat#103015-100
Seahorse XF Glycolysis Stress Test Kit	Seahorse Bioscience	Cat#103020-100
Enhanced ATP Assay Kit	Beyotime	Cat#S0027
BCA protein assay Kit	Cowin Biotech	Cat#CW0014
Mouse Insulin ELISA Kit	Abclonal	Cat#RK09268
Mouse Adiponectin ELISA Kit	Abclonal	Cat#RK02574
Mouse FFA ELISA Kit	MEIMIAN	Cat#MM-0326
Lactic Acid Assay Kit	Solarbio	Cat#BC2235
Complex Ⅴ Activity Assay Kit	Solarbio	Cat#BC1445
Biotin Northern Blot Kit	Beyotime	Cat#R0219
Evo M-MLV RT Reaction Mix	Accurate	Cat#AG11728
SYBR Green Supermix	Accurate	Cat#AG11701
Chemicals, peptides, and recombinant proteins		
Collagenase II	Sigma-Aldrich	Cat#1148090
Insulin	Sigma-Aldrich	Cat#I2643
Dexamethasone	Sigma-Aldrich	Cat#D4902
IBMX	Sigma-Aldrich	Cat#I5879
BASIC DMEM, High Glucose	Gibco	Cat#C11995500BT
BODIPY 493/503	Invitrogen	Cat#D3922
DAPI Fluoromount-G	Southern Biotech	Cat#0100-20
MitoTracker Red (1:1000)	Invitrogen	Cat#M7512
TRIzol	TAKARA	Cat# 15596026
Actinomycin D	MCE	Cat#HY-17559
RNase R	Epicentre	Cat#RNR07250
Oligo(dT) primers	Accurate	Cat#11612
Lipofectamin RNAiMAX	Invitrogen	Cat#13778150
Lipofectamine 2000	Invitrogen	Cat#11668019
RNA Ladder	Servicebio	Cat#G3372
100 bp DNA Ladder	Vazyme	Cat#MD104
Multicolor Prestained Protein Ladder	EpiZyme	Cat#WJ103
RNA Loading Buffer	Beyotime	Cat#R0215
Protein Sample Loading Buffer	EpiZyme	Cat#LT101
Blue Native PAGE Loading Buffer (2X)	Beyotime	Cat#P0761
High MW non-denaturing protein Marker	Coolaber	Cat#DM2004
Nylon membrane	Merck millipore	Cat#INYC00010
Streptavidin magnetic beads	MCE	Cat#HY-K0208
Protein A/G beads	MCE	Cat#HY-K0202
Proteinase K	Invitrogen	Cat# AM2548

Reagent or Resource	Source	Identifier
RNase inhibitor	Promega	Cat# N2511
Acetonitrile	Sigma-Aldrich	Cat# 271004
Dithiothreitol	Sigma-Aldrich	Cat# 43815
Iodoacetamide	Sigma-Aldrich	Cat#A3221
Ammonium bicarbonate	Sigma-Aldrich	Cat# A6141
Seahorse XF Base Medium	Seahorse Bioscience	Cat#103193
CCK-8 reagent	DOJINDO	Cat#CK04
4–13% blue native precast PAGE gel	Beyotime	Cat#P0546
Blue Native PAGE Cathode Buffer I (5X)	Beyotime	Cat#P0765
Blue Native PAGE Cathode Buffer II (5X)	Beyotime	Cat#P0767
Blue Native PAGE Anode Buffer (5X)	Beyotime	Cat#P0769
Coomassie R-250	Beyotime	Cat#P0017
Rapamycin	MCE	Cat#HY-10219
Palmitate	KunChuang	Cat#KC002
Recombinant mouse ATP5A	Abmart	Cat#RG508769
Recombinant mouse ATP5B	Abmart	Cat#EHM2350
Software and Algorithms		
GraphPad Prism	GraphPad Software, Inc	https://www.graphpad.com/scientific-software/prism/
Leica software package	Leica	N/A
ImageJ software	NIH	https://imagej.nih.gov/ij/
SPSS 23.0	International Business Machines Corporation	http://www.spss.com.cn/3
Primer Premier	PREMIER Biosoft International	http://www.premierbiosoft.com/primerdesign/
Coding potential calculator	Kang et al., 2017	https://cpc.gao-lab.org/
miRANDA	John et al., 2004	http://www.microrna.org/microrna/home.do
TargetScan	Agarwal et al., 2015	http://www.targetscan.org/vert_72/

Supplementary Table S4. Key characteristics of the human adipose tissue donors

Number	sex	age (years)	height (m)	weight (kd)	BMI
lean-1	female	44	1.55	55	23
lean-2	male	35	1.72	68	23
lean-3	female	35	1.60	59	23
lean-4	male	48	1.72	69	23
lean-5	female	59	1.55	57	24
lean-6	male	51	1.65	65	24
lean-7	female	42	1.66	66	24
lean-8	male	54	1.66	67	24
lean-9	male	38	1.75	75	24
Obese-1	female	68	1.57	70	28
Obese-2	male	54	1.72	85	29
Obese-3	female	52	1.60	75	29
Obese-4	male	66	1.70	70	24
Obese-5	female	53	1.58	79	32
Obese-6	male	39	1.86	109	32
Obese-7	male	50	1.71	95	32
Obese-8	female	51	1.55	82	34
Obese-9	female	65	1.56	86	35
