# Supplementary material for: Mitochondria-localized circRNA BRAKE restrains adipogenesis under nutrient overload by disrupting ATP synthase assembly in mice
Source: Nat Commun. 2026 Oct 13;17:11577. doi: 10.1038/s41467-026-78378-z (PMC13752303; doi:10.1038/s41467-026-78378-z)
Supplement: Supplementary file 2 [file 41467_2026_78378_MOESM2_ESM.pdf]

nature portfolio

Peer Review File

Mitochondria-localized circRNA BRAKE restrains adipogenesis under nutrient overload by disrupting ATP synthase assembly

Corresponding Author: Dr Yanrui Huang

This file contains all reviewer reports in order by version, followed by all author rebuttals in order by version.

Version 0:

Reviewer comments:

Reviewer #1

(Remarks to the Author)
The authors identify a mitochondria-enriched circRNA, circBRAKE, that is elevated in obese mouse and human adipose tissue and negatively correlates with mitochondrial ATP production and adipogenic capacity. Adipocyte-specific circBRAKE knockdown reduces adiposity and improves metabolic phenotypes in mice, and mechanistically circBRAKE is proposed to bind ATP5A to impair Complex V function and downstream mTORC1 signaling, thereby restraining adipogenesis. Overall, the identification of a mitochondria-associated circRNA is intriguing and potentially novel. The adipocyte-specific knockdown (AKD) mouse model is also a strength, given the limited in vivo evidence for circRNA function in metabolic tissues. However, there are some major concerns that need to be addressed.
1) Logical Mismatch in the Adipoq-Cre Model: The authors propose that circBRAKE restrains adipogenesis. However, the authors stated that “circRNA BRAKE knockdown in our model occurs only after adipocyte differentiation”. This creates a logical mismatch: phenotypes arising from post-differentiation deletion cannot be straightforwardly attributed to a primary effect on adipogenic differentiation of progenitors.
2) Disconnect between in vitro mechanism and in vivo phenotypes. The manuscirpt indicates that the core mechanistic model—enhanced adipogenesis driven by a circBRAKE–ATP5A–mitochondria–mTORC1 axis—is supported primarily by in vitro experiments in APC and 3T3-L1 cells. This raises another central concern whether these findings are of physiological relevance. Key mechanistic readouts (mitochondrial ATP production/bioenergetics, Complex V function, mTORC1 activation, and adipogenic marker programs) but are not shown to be altered in adipose tissue from AKD mice under the relevant conditions. As a result, it remains unclear whether the cellular phenotypes largely reflect culture-dependent behavior. To strengthen causality, the authors should directly test the proposed mechanism in adipose tissue in vivo—for example by assessing mitochondrial bioenergetic outputs/Complex V function and canonical mTORC1 activity readouts in WAT from AKD versus control mice, alongside adipogenesis-related endpoints.
3) Normalization/controls are insufficiently described, which complicates interpretation. 23.1) “Mitochondrial enrichment” of circBRAKE Fig. 1E–F. Given you normalize mito-fraction qPCR to mtCOX2, it will helpful to confirm if mtCOX2 contents in mitochondria is stabble between control and ob/ob mice. Also examine if the enrichment persist in HFD model, which is more relavlent to obesity. 3.2) Fig. 4M, 6A. Because OXPHOS blots are normalized to ACTB, please include mitochondrial number controls such as mtDNA to distinguish mitochondria number difference from increased OXPHOS per mitochondrion. 3.3). ATP is normalized to total protein(Figure 5K; 6B, D, F); please also report/justify whether conclusions hold after adjusting for mitochondrial abundance (e.g., mtDNA copy number or TOM20/VDAC1), particularly for obese adipose tissue where tissue mass/protein content may not reflect mitochondrial input.
4) Fraction purity controls (mitochondrial isolation).Wherever mitochondrial isolation/fractionation is central to the conclusion (e.g., “mitochondria-enriched” circBRAKE or mitochondrial protein/RNA changes), please provide fraction purity controls for the exact samples shown.
5) Differentiation dependence of mitochondrial localization of circBRAKE. To support the claim that circBRAKE accumulates in mitochondria in adipogenic cells, it is helpful to assess whether mitochondrial partitioning changes with differentiation.
6) Model system alignment and terminology: “APCs” vs 3T3-L1. Although 3T3-L1 cells remain a useful model for adipogenic signaling, their fibroblast originand clonal selection impose important limitations. Consequently, there is growing emphasis in the field on primary SVF-derived adipose progenitor cells to assess adipogenesis and metabolic regulation. Thus, some important observations such as BRAKE–ATP5A interaction (Figure 5A-F), Gain-of-function for circBRAKE (Figure 5G-K, Fig S5),) should be confirmed in SVF-derived APC.

7) BRAKE–ATP5A interaction. The specificity controls for the BRAKE–ATP5A interaction should be strengthened. Given that circRNA overexpression can promote non-physiological interactions, it would also be important to show that endogenous circBRAKE is sufficient to influence ATP5A–ATP5B association/Complex V assembly—for example by comparing control vs AKD SVF-derived APCs/adipocytes (or performing rescue with binding-deficient BRAKE), using the same co-IP readout.

Minor Comments

1) Food intake (metabolic cage). The text states food intake is unchanged, but the plots appear to differ at least during the dark/active phase. Please clarify whether the conclusion is based on 24-h cumulative intake or phase-specific intake, and state which metric and statistical test support the statement.

2) Tissue specificity of improved insulin sensitivity.The claim that improved glucose metabolism is "primarily driven by enhanced eWAT insulin sensitivity" needs support. Please provide insulin signaling readouts in liver and skeletal muscle (e.g., insulin-stimulated AKT phosphorylation), or soften the conclusion.

3) ATP5A–ATP5B interaction.An ATP5A–ATP5B co-IP (Figure 5F) reports Complex V subunit association and does not directly demonstrate circBRAKE–ATP5A binding. Please revise wording accordingly and cite RNA pulldown/RIP as the binding evidence.

Reviewer #2

(Remarks to the Author)

This study identified a circRNA formed by Birc6 exons 63–64, termed as circRNA BRAKE that played critical roles in regulating adipogenesis. The authors provided several lines of evidence to support that circRNA BRAKE interacted with ATP5A to suppress ATP synthesis in mitochondria, and therefore inhibited adipogenesis.

I have several concerns here.

1, In the introduction, the authors need to distinguish mitochondria-encoded circRNAs from mitochondria-localized circRNAs that encoded by the nuclear genome. In the discussion, the authors need to speculate the mechanism about how BRAKE is imported into mitochondria. Also, other circRNAs that are reported to interact with ATP5A/B (e.g., PMID: 39973625) should also be discussed.

2, Figure 1. The BRAKE copy numbers per cell and per mitochondrion (as quantified in relative to mitochondrial DNA copy number) in eWAT and 3T3-L1 cells should be evaluated. The can be done with qRT-PCR with a standard curve. 1A, the X-axis should labeled as "-4 -2 0 2 4".

3, Figure 2. The protein levels of Birc6 should be examined in the conditional knockout mice (both in the control and in the AKD, and also under normal/high fat diet). BIRC6 is an important anti-apoptotic protein, and the authors need to speculate the potential relationship of BRAKE and BIRC6 mRNA (supposedly they are subjected to the same transcriptional regulation, although may compete in the biogenesis?)

4, Figure 3. For 3I, please show images with all samples. 3J, the adipocyte diameter analysis was from 3 cells? More cells should be quantified.

5, Figure 4 and Figure 5. The authors demonstrate that BRAKE affects the mRNA levels of specific GO pathways in fig. 4, and then display BRAKE interact with ATP5A protein in fig. 5. The authors need to at least provide some solid references to support that the ATP5A–mTORC1 pathway eventually regulates the gene expression of specific GO pathways in fig. 4. If there are not enough supporting information, the authors need to provide some experimental data.

6, Figure 6. In the figure legends of 6C, 6D, and 6F, and also in the main text, not sure what "mitochondrial expression of circRNA BRAKE" means? Should it be "mitochondrial levels of circRNA BRAKE"?

Reviewer #3

(Remarks to the Author)

The manuscript investigates the functional role of a mitochondria-localized circular RNA, termed circRNA BRAKE, in the regulation of adipogenesis and metabolic homeostasis under conditions of nutrient overload. Using a comprehensive experimental approach that integrates genetically modified mouse models, in vitro adipocyte systems, transcriptomic profiling, mitochondrial bioenergetic analyses, and RNA–protein interaction assays, the authors demonstrate that circRNA BRAKE directly interacts with the ATP synthase subunit ATP5A. This interaction disrupts Complex V assembly, reduces ATP production, and suppresses adipogenesis through inhibition of mTORC1 signaling. Overall, the study addresses an important and timely question regarding the role of mitochondrial circRNAs in metabolic regulation.

General comment

While the data are extensive and largely convincing, several methodological and interpretative issues require clarification to strengthen the rigor and reproducibility of the study.

Major Comments

1.Studies showed a clear heterogeneity of white adipose tissues in both humans and mice, with significant differences regarding energy metabolism and mitochondrial regulation (PMID: 26964831, 34935088, 29507687). Considering that eWAT showed a lower APC deposition and less response in mitochondrial metabolism during obesity compared to BAT or subcutaneous WAT, why was the eWAT chosen in the study to be investigated? This matter should be clear in the manuscript.

2.The circRNA BRAKE knockdown occurs exclusively in mature adipocytes due to the use of the Adiponectin-Cre system. Nevertheless, several conclusions extend to adipogenic processes occurring at earlier stages in vivo. Although in vitro experiments partially address this limitation, the manuscript would benefit from a clearer acknowledgment of this constraint and a more cautious interpretation of in vivo adipogenesis-related findings.

3.In the figure 4 L, the authors described “Seahorse XF24 analysis confirmed enhanced maximal oxygen consumption rate (OCR) in AKD APCs”, however, there is no statistical symbol in the figure. What is the number of samples used in this experiment?

4.In the figure 4M, the absence of circRNA BRAKE in AKD increased the protein content of the components of the mitochondrial complexes encoded by nuclear and mitochondrial DNA. How does circRNA BRAKE promote these effects?

5.The data presented do not allow us to conclude that circRNA BRAKE alters ATPase assembly. Although ATPase activity has been monitored, ATPase integrity requires other techniques. For example, the Blue Native page (BN-page) assay would allow the authors to state that circRNA BRAKE affects ATPase assembly.

6.The link between ATP production and mTORC1 signaling needs to be explored.

7.It was attested in the results (line 122, figure 1I) that immunofluorescence analysis was used to identify circRNA BRAKE accumulation in eWAT, but the method used is not in the methodology of the study. The immunofluorescence protocol should be included in the methodology.

Minor Comments
1. Terminology related to circRNA BRAKE (e.g., circBirc6 versus circRNA BRAKE) should be standardized throughout the manuscript after the initial definition.

2. The authors could consider replacing the term Complex V with ATPase throughout the text.

3. Minor typographical and formatting issues are present (e.g., missing spaces, concatenated words, and inconsistent unit formatting) and should be corrected.

4. Some writing errors were found, for example: mROC1 (mTORC1), line 97 and "funciton", line 99.

5. The Discussion section could be strengthened by a more direct comparison with previously described mitochondria-localized circRNAs, highlighting similarities and differences in mechanism.

6. In some sections, particularly the Introduction and Discussion, the tone could be slightly moderated to avoid overly definitive statements and better reflect the mechanistic and exploratory nature of the study.

7. Figure S1. Student’ s t-test used in this figure is not appropriate.

8. Figure S3. The description in the caption for figures S3C and F is reversed. Statistical symbols are missing in Figure S3F. Furthermore, the number of samples used in the mitochondrial respiration experiment is too low.

Version 1:

Reviewer comments:

Reviewer #1

(Remarks to the Author)
The authors have addressed my concerns. I have no further comment.

Reviewer #2

(Remarks to the Author)
All my concerns have been successfully addressed.

Reviewer #3

(Remarks to the Author)
The authors have satisfactorily addressed most of my major concerns and have substantially strengthened the manuscript

by performing several additional experiments (including BN-PAGE, rapamycin and oligomycin treatments, expanded Seahorse analyses, and methodological controls).

I particularly appreciate that the authors explicitly acknowledged the limitations of the Adiponectin-Cre model and appropriately moderated the interpretation of the in vivo adipogenesis data. Likewise, the oligomycin experiments provide important mechanistic support for the proposed relationship between ATP production and mTORC1 activation.

One point that remains only partially resolved concerns the mechanism underlying the increased abundance of OXPHOS proteins following circRNA BRAKE knockdown. The new rapamycin experiments convincingly demonstrate that these changes are at least partly mTORC1-dependent. However, they do not fully explain how proteins encoded by the mitochondrial genome are coordinately increased, given that mTORC1 primarily regulates cytosolic protein synthesis. Although this mechanistic aspect remains incompletely understood, I do not believe that it detracts from the main conclusions of the study.

Overall, the revised manuscript is substantially improved, the conclusions are adequately supported by the available data, and I have no further major concerns. I believe the manuscript is suitable for publication after minor editorial revisions.

Open Access This Peer Review File is licensed under a Creative Commons Attribution 4.0 International License, which permits use, sharing, adaptation, distribution and reproduction in any medium or format, as long as you give appropriate credit to the original author(s) and the source, provide a link to the Creative Commons license, and indicate if changes were made.
In cases where reviewers are anonymous, credit should be given to 'Anonymous Referee' and the source.
The images or other third party material in this Peer Review File are included in the article’s Creative Commons license, unless indicated otherwise in a credit line to the material. If material is not included in the article’s Creative Commons license and your intended use is not permitted by statutory regulation or exceeds the permitted use, you will need to obtain permission directly from the copyright holder.
To view a copy of this license, visit https://creativecommons.org/licenses/by/4.0/

POINT-TO-POINT RESPONSE TO REVIEWERS' COMMENTS

RESPONSE TO REVIEWER 1

General comment: The authors identify a mitochondria-enriched circRNA, circBRAKE, that is elevated in obese mouse and human adipose tissue and negatively correlates with mitochondrial ATP production and adipogenic capacity. Adipocyte-specific circBRAKE knockdown reduces adiposity and improves metabolic phenotypes in mice, and mechanistically circBRAKE is proposed to bind ATP5A to impair Complex V function and downstream mTORC1 signaling, thereby restraining adipogenesis. Overall, the identification of a mitochondria-associated circRNA is intriguing and potentially novel. The adipocyte-specific knockdown (AKD) mouse model is also a strength, given the limited in vivo evidence for circRNA function in metabolic tissues. However, there are some major concerns that need to be addressed.

Major comment #1: Logical Mismatch in the Adipoq-Cre Model: The authors propose that circBRAKE restrains adipogenesis. However, the authors stated that "circRNA BRAKE knockdown in our model occurs only after adipocyte differentiation". This creates a logical mismatch: phenotypes arising from post-differentiation deletion cannot be straight forwardly attributed to a primary effect on adipogenic differentiation of progenitors.

We thank the reviewer for raising this important concern. We agree that Adiponectin-Cre–mediated circRNA BRAKE knockdown occurs predominantly after adipocyte differentiation and therefore does not, per se, establish a primary role for circRNA BRAKE in the early differentiation of adipocyte progenitors in vivo. To address this issue, we have revised the manuscript to distinguish the conclusions supported by the in vivo model from those derived from primary cell experiments. Upon ex vivo differentiation, APCs isolated from AKD eWAT showed reduced circRNA BRAKE levels and enhanced adipogenic capacity, while complementary loss-of-function, gain-of-function, and ATP5A-binding-deficient mutant experiments in primary eWAT SVF-derived APCs directly demonstrated a role of circRNA BRAKE in regulating adipogenic differentiation (Figure 4 and 5). In addition, AKD eWAT showed increased ATP production, ATP synthase activity, and mTORC1 signaling, indicating that the key mitochondrial bioenergetic and mTORC1-related alterations are also present in adipose tissue in vivo(Figure S5).

Nevertheless, we acknowledge that the Adiponectin-Cre model cannot directly define the stage-

specific role of circRNA BRAKE during early adipogenesis in vivo. We have therefore revised the manuscript to interpret the in vivo adipogenesis-related findings more cautiously. We also clarified in the Discussion that future studies using progenitor-targeting or inducible Cre models will be required to determine the stage-specific function of circRNA BRAKE during adipose tissue development and remodeling.

Major comment #2: Disconnect between in vitro mechanism and in vivo phenotypes. The manuscript indicates that the core mechanistic model—enhanced adipogenesis driven by a circBRAKE–ATP5A–mitochondria–mTORC1 axis—is supported primarily by in vitro experiments in APC and 3T3-L1 cells. This raises another central concern whether these findings are of physiological relevance. Key mechanistic readouts (mitochondrial ATP production/bioenergetics, Complex V function, mTORC1 activation, and adipogenic marker programs) but are not shown to be altered in adipose tissue from AKD mice under the relevant conditions. As a result, it remains unclear whether the cellular phenotypes largely reflect culture-dependent behavior. To strengthen causality, the authors should directly test the proposed mechanism in adipose tissue in vivo—for example by assessing mitochondrial bioenergetic outputs/Complex V function and canonical mTORC1 activity readouts in WAT from AKD versus control mice, alongside adipogenesis-related endpoints.

We fully agree with the reviewer that the physiological relevance of the circRNA BRAKE–ATP5A–mitochondria–mTORC1 axis should be directly evaluated in adipose tissue from AKD mice. To address this concern, we performed additional analyses in eWAT from control and AKD mice. Consistent with our cellular findings, AKD eWAT exhibited increased ATP production (Figure S5A), enhanced mitochondrial oxidative capacity (Figure S5B), and increased ATP synthase activity (Figure S5D). AKD eWAT also showed enhanced mTORC1 signaling, as indicated by elevated pS6K/S6K levels (Figure S5E), while circulating adiponectin levels were increased in AKD mice (Figure S5C). These tissue-level data recapitulate the key mitochondrial bioenergetic and mTORC1-related changes observed in primary APCs and support the physiological relevance of the proposed mechanism rather than a phenotype restricted to cultured cells.

Major comment #3: Normalization/controls are insufficiently described, which complicates interpretation. 23.1) "Mitochondrial enrichment" of circBRAKE Fig. 1E–F. Given you normalize mito-fraction qPCR to mtCOX2, it will helpful to confirm if mtCOX2 contents in mitochondria is stable between control and *ob/ob* mice. Also examine if the enrichment persist in HFD model, which is more relavent to obesity.

3.2) Fig. 4M, 6A. Because OXPHOS blots are normalized to ACTB, please include mitochondrial number controls such as mtDNA to distinguish mitochondria number difference from increased OXPHOS per mitochondrion. ATP is normalized to total protein(Figure 5K; 6B, D, F); please also report/justify whether conclusions hold after adjusting for mitochondrial abundance (e.g., mtDNA copy number or TOM20/VDAC1), particularly for obese adipose tissue where tissue mass/protein content may not reflect mitochondrial input. 4M、6A。

We apologize for not describing the normalization procedures and corresponding controls with sufficient clarity in the original manuscript, which may have complicated the interpretation of our results.

3.1) We agree that the stability of mtCOX2 should be confirmed because it was used as the reference for mitochondrial-fraction qRT-PCR normalization. We therefore examined mtCOX2 abundance in the mitochondrial and cytosolic fractions of eWAT from control and ob/ob mice. As shown in Figure S1E, mtCOX2 was strongly enriched in the mitochondrial fractions and depleted from the cytosolic fractions, whereas Actb mRNA showed the opposite distribution. Importantly, using Tom20 mRNA as the mitochondrial loading control, mtCOX2 levels in the mitochondrial fractions were comparable between control and ob/ob mice, supporting its use as a reference for normalization of mitochondrial RNA abundance (see panel below).

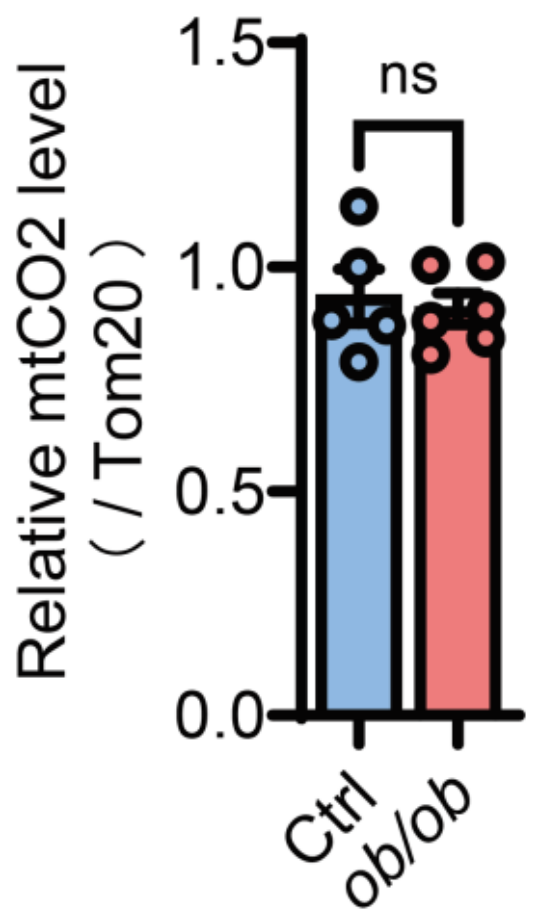

We also extended the analysis to a diet-induced obesity model. Absolute qRT-PCR analysis of purified eWAT mitochondrial fractions showed that mitochondrial circRNA BRAKE abundance, expressed as copies per cell, was significantly increased in HFD-fed mice compared with NCD-fed controls (Figure S1C-D). These results indicate that the obesity-associated mitochondrial accumulation of circRNA BRAKE is not restricted to the genetic ob/ob model but is also observed in diet-induced obesity.

3.2）To determine whether the observed changes in OXPHOS protein levels reflected altered mitochondrial abundance or altered OXPHOS abundance per mitochondrion, we performed new immunoblotting experiments for Figures 4M, 6A, S3D, S5B and S8D using TOM20 as the mitochondrial loading control. The differences in OXPHOS protein levels remained significant after normalization to TOM20, indicating that they were not solely attributable to altered mitochondrial abundance. We also performed new ATP measurements for Figures 4K, 6B, 6D, S3B, S3E, S5A, S6D, S6M and S7D and normalized ATP levels to mtDNA copy number. The direction and statistical significance of the results remained unchanged, supporting the conclusion that the observed ATP differences reflect altered mitochondrial bioenergetic function rather than differences in mitochondrial abundance alone.

Major comment #4: Fraction purity controls (mitochondrial isolation). Wherever mitochondrial isolation/fractionation is central to the conclusion (e.g., “mitochondria-enriched” circBRAKE or mitochondrial protein/RNA changes), please provide fraction purity controls for the exact samples shown.

In response to the reviewer's suggestion, we added fraction-purity controls for the corresponding mitochondrial and cytosolic preparations used in the fractionation-dependent analyses from control and ob//ob mice. qRT-PCR showed enrichment of mtCOX2 and depletion of Actb in mitochondrial fractions, while immunoblotting confirmed enrichment of TOM20 and depletion of GAPDH across the examined metabolic tissues (Figures S1E–F). These data support the purity of the mitochondrial fractions used for circRNA BRAKE and mitochondrial protein/RNA analyses.

Major comment #5: Differentiation dependence of mitochondrial localization of circBRAKE. To support the claim that circBRAKE accumulates in mitochondria in adipogenic cells, it is helpful to assess whether mitochondrial partitioning changes with differentiation.

To determine whether mitochondrial accumulation of circRNA BRAKE changes during adipogenic differentiation, we performed mitochondrial fractionation followed by qRT-PCR in eWAT-derived APCs from ob/ob and control mice at differentiation days 0, 3, and 6. CircRNA BRAKE levels in mitochondrial fractions increased over the course of differentiation and were significantly higher in ob/ob-derived cells at days 3 and 6, whereas no significant difference was observed at day 0 (Figure S3J). These data support differentiation-associated accumulation of circRNA BRAKE in mitochondria of adipogenic cells.

Major comment #6: Model system alignment and terminology: "APCs" vs 3T3-L1. Although 3T3-L1 cells remain a useful model for adipogenic signaling, their fibroblast originand clonal selection impose important limitations. Consequently, there is growing emphasis in the field on primary SVF-derived adipose progenitor cells to assess adipogenesis and metabolic regulation. Thus, some important observations such as BRAKE–ATP5A interaction (Figure 5A-F), Gain-of-function for circBRAKE (Figure 5G-K, Fig S5),) should be confirmed in SVF-derived APC.

We agree with the reviewer that primary SVF-derived adipose progenitor cells are more physiologically relevant than 3T3-L1 cells for investigating adipogenic differentiation and metabolic regulation.

We apologize that the experimental models were not specified with sufficient clarity in the original manuscript. We would like to clarify that the core mechanistic experiments presented in Figure 5

were not performed in 3T3-L1 cells. Specifically, mitochondrial protein extracts from ob/ob eWAT were used for RNA pull-down, mass spectrometry, and immunoblotting validation in Figures 5A–C. Figures 5D and 5J involved cell-free assays using purified recombinant proteins and synthetic RNA fragments corresponding to circRNA BRAKE to establish direct binding to ATP5A and identify nucleotides 67–134 as the ATP5A-binding region.

The remaining cell-based experiments in Figure 5 were conducted in primary SVF-derived APCs. ATP5A-RIP, ATP5A–ATP5B co-immunoprecipitation, ATP synthase activity, and BN-PAGE analyses were performed in primary APCs derived from control mice (Figures 5E–H and 5K–N). The effects of the ATP5A-binding-deficient circRNA BRAKE mutant on ATP production and adipogenic differentiation were evaluated in primary APCs derived from AKD mice (Figures 5O–P and 5R–S). We have revised the Results and figure legends to clearly specify the experimental material used in each panel.

Nevertheless, to further align the experimental systems and address the reviewer's concern, we repeated the experiments that had previously been performed in 3T3-L1 cells using primary SVF-derived APCs from control mice, including siRNA-mediated circRNA BRAKE knockdown and lentivirus-mediated circRNA BRAKE overexpression. The knockdown and overexpression efficiencies in primary APCs are shown in Figures S6B and S6K, respectively. ***Accordingly, the previous 3T3-L1-based data in Figure S6 have been replaced with results obtained from primary mouse APCs. Moreover, all newly added mechanistic experiments presented in the revised Figure 5 were performed in primary mouse APCs.***

Major comment #7: BRAKE–ATP5A interaction. The specificity controls for the BRAKE–ATP5A interaction should be strengthened. Given that circRNA overexpression can promote non-physiological interactions, it would also be important to show that endogenous circBRAKE is sufficient to influence ATP5A–ATP5B association/Complex V assembly—for example by comparing control vs AKD SVF-derived APCs/adipocytes (or performing rescue with binding-deficient BRAKE), using the same co-IP readout.

We agree that both endogenous loss-of-function evidence and additional rescue experiments using binding-deficient BRAKE are needed to establish the physiological relevance of the circRNA BRAKE–ATP5A mechanism.

To determine whether endogenous circRNA BRAKE regulates ATP5A–ATP5B association, we performed ATP5A co-immunoprecipitation in differentiated primary eWAT SVF-derived APCs from control and AKD mice. Endogenous circRNA BRAKE depletion in AKD APCs increased ATP5A–ATP5B association compared with control APCs (see panel below), supporting the conclusion that endogenous circRNA BRAKE restrains ATP synthase subunit association.

We also performed rescue experiments using binding-deficient BRAKE in primary APCs derived from control mice. Wild-type circRNA BRAKE reduced ATP5A–ATP5B association and ATP synthase activity, whereas the binding-deficient mutant largely abolished these effects (Figures 5L–M). Together, these findings demonstrate that circRNA BRAKE regulates ATP5A–ATP5B association and ATP synthase assembly/function through its ATP5A-binding region.

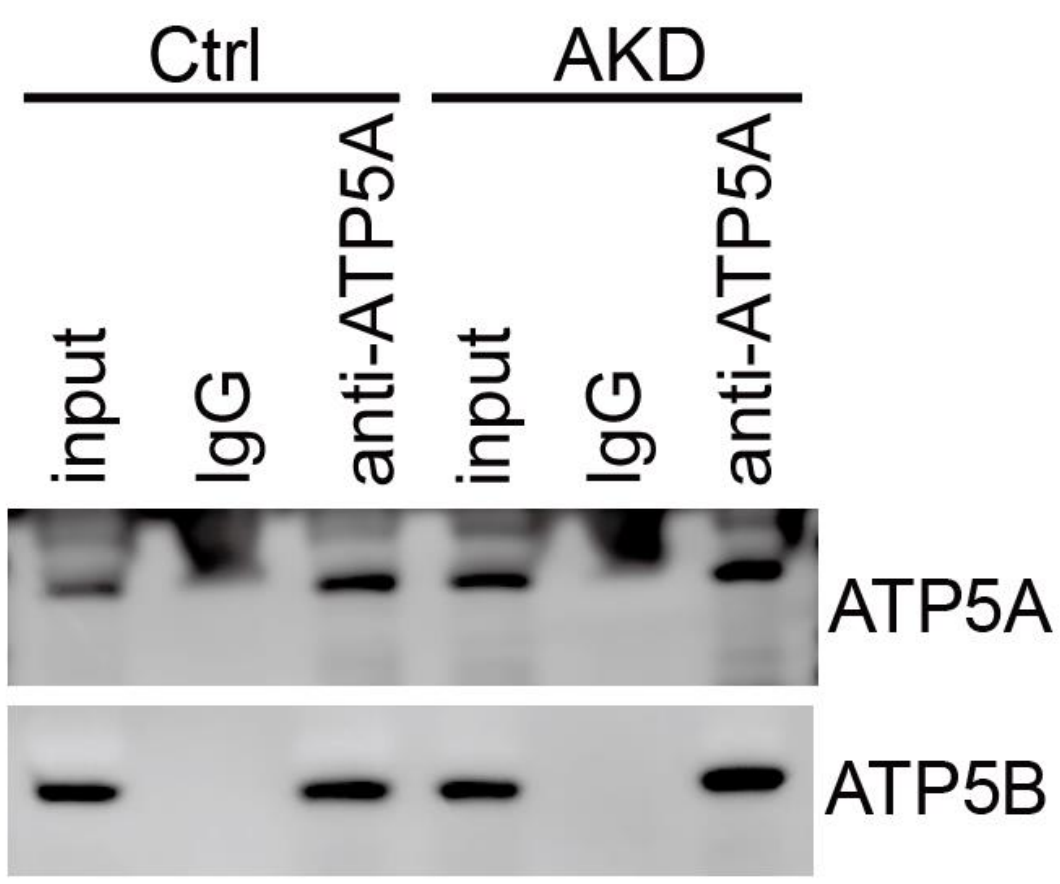

Minor comment #1: Food intake (metabolic cage). The text states food intake is unchanged, but the plots appear to differ at least during the dark/active phase. Please clarify whether the conclusion is based on 24-h cumulative intake or phase-specific intake, and state which metric and statistical test support the statement.

We agree that the original statement that food intake was "unchanged" was not sufficiently precise. We have revised the text as follows: "Food intake was not reduced in AKD mice and was even increased during the dark phase under chow diet, whereas HFD-fed AKD mice showed comparable food intake to controls". This conclusion is based on phase-specific mean food intake during the light and dark phases, rather than only 24-h cumulative intake. Using two-way ANOVA followed by Šídák's multiple comparisons test, we found that chow-fed AKD mice showed no

significant difference in food intake during the light phase but exhibited increased food intake during the dark phase, whereas HFD-fed AKD mice showed no significant difference in food intake during either phase.

Minor comment #2: Tissue specificity of improved insulin sensitivity.The claim that improved glucose metabolism is "primarily driven by enhanced eWAT insulin sensitivity" needs support. Please provide insulin signaling readouts in liver and skeletal muscle (e.g., insulin-stimulated AKT phosphorylation), or soften the conclusion.

To better assess tissue-specific insulin signaling, we examined insulin-stimulated AKT phosphorylation in eWAT, liver, and skeletal muscle. Insulin-stimulated AKT phosphorylation was increased not only in eWAT but also in the liver and skeletal muscle of AKD mice (Figure S4B). These findings indicate that AKD improves insulin responsiveness across multiple metabolic tissues rather than exclusively in eWAT.

Accordingly, we have revised the manuscript to remove the statement that the improvement in glucose metabolism was primarily driven by enhanced eWAT insulin sensitivity. We now interpret the findings as evidence of improved systemic insulin sensitivity across multiple metabolic tissues, which may be secondary to improved adipose tissue function and lipid homeostasis.

Minor comment #3: ATP5A–ATP5B interaction. An ATP5A–ATP5B co-IP (Figure 5F) reports Complex V subunit association and does not directly demonstrate circBRAKE–ATP5A binding. Please revise wording accordingly and cite RNA pulldown/RIP as the binding evidence.

We agree that the ATP5A–ATP5B co-IP assay reflects the interaction between ATP synthase subunits and should not be used as direct evidence for circRNA BRAKE–ATP5A binding. In the revised manuscript, the cell-free RNA pull-down assay using purified recombinant ATP5A is cited as evidence of direct binding between circRNA BRAKE and ATP5A (Figure 5D), while the ATP5A-RIP assay supports their association in primary APCs (Figure 5E). In contrast, the ATP5A–ATP5B co-immunoprecipitation assay is described as evidence that circRNA BRAKE modulates the association between ATP synthase subunits (Figure 5F).

RESPONSE TO REVIEWER 2

Major comment #1: In the introduction, the authors need to distinguish mitochondria-encoded circRNAs from mitochondria-localized circRNAs that encoded by the nuclear genome. In the discussion, the authors need to speculate the mechanism about how BRAKE is imported into mitochondria. Also, other circRNAs that are reported to interact with ATP5A/B (e.g., PMID: 39973625) should also be discussed.

We thank the reviewer for these important suggestions.
In response to the reviewer's first point, we agree that the original Introduction did not clearly distinguish mitochondrial genome-encoded circRNAs from nuclear genome-derived circRNAs that subsequently localize to mitochondria. To address this concern, we have revised the Introduction to explicitly describe these two categories of mitochondria-associated circRNAs and to clarify that circSamd4, circPUM1, and circRNA BRAKE discussed in our study belong to the latter category.
Regarding the reviewer's second point, we have added a discussion of the potential mechanisms underlying the mitochondrial import of nuclear genome-derived circRNA BRAKE. The revised text reads as follows:
"How nuclear genome-derived circRNA BRAKE is imported into mitochondria remains an important unresolved question. Previous studies have implicated PNPASE/PNPT1 in the mitochondrial import of selected nuclear-encoded RNAs, partly through the recognition of specific RNA stem-loop structures 36. Moreover, mecciRNAs have been shown to interact with nuclear-encoded proteins, including RPA32, RPA70, and members of the hnRNPA family, and to facilitate their mitochondrial import16. Given that circRNA BRAKE directly binds ATP5A, one possibility is that circRNA BRAKE may associate with newly synthesized ATP5A before its mitochondrial translocation and subsequently be co-imported with ATP5A or retained within mitochondria through its association with ATP5A-containing complexes. This possibility warrants further investigation."
In response to the reviewer's third point, we have expanded the Discussion to include previous reports showing that circRNAs can interact with ATP synthase subunits and other mPTP-related proteins. The revised text reads as follows:

"Our findings are also consistent with emerging evidence that mitochondria-associated circRNAs can interact with ATP synthase subunits. For instance, the mitochondrial genome-encoded circRNA SCAR was reported to bind ATP5B and regulate mPTP-associated mitochondrial ROS production. Recent work further demonstrated that multiple mecciRNAs interact with mPTP-related proteins, including ATP5B 17,53. Compared with these mitochondria-encoded circRNAs, circRNA BRAKE represents a nuclear genome-derived mitochondria-localized circRNA that directly targets ATP5A, disrupts the ATP5A–ATP5B interaction, and regulates ATP synthase assembly in adipocytes. Thus, our study extends the functional spectrum of nuclear genome-derived mitochondria-localized circRNAs in regulating mitochondrial protein interactions, bioenergetic function, and systemic metabolism."

Major comment #2: Figure 1. The BRAKE copy numbers per cell and per mitochondrion (as quantified in relative to mitochondrial DNA copy number) in eWAT and 3T3-L1 cells should be evaluated. The can be done with qRT-PCR with a standard curve. 1A, the X-axis should labeled as "-4 -2 0 2 4".

Per the reviewer's suggestion, we performed absolute qRT-PCR and quantified circRNA BRAKE copy number per cell and per mitochondrion. These new data have been added to Figure S1A-S1D. We also revised the X-axis labeling in Fig. 1A to "−4, −2, 0, 2, 4" as suggested.

Major comment #3: Figure 2. The protein levels of Birc6 should be examined in the conditional knockout mice (both in the control and in the AKD, and also under normal/high fat diet). BIRC6 is an important anti-apoptotic protein, and the authors need to speculate the potential relationship of BRAKE and BIRC6 mRNA (supposedly they are subjected to the same transcriptional regulation, although may compete in the biogenesis?)

Per the reviewer's suggestion, we examined BIRC6 protein levels in eWAT from control and AKD mice under both chow and high-fat diet conditions (Figure 2H). Consistent with the unchanged linear Birc6 mRNA levels, BIRC6 protein abundance in eWAT was not significantly altered, indicating that deletion of the Alu2–4 elements does not affect host-gene protein expression.
We have also revised the Discussion to address the potential relationship between circRNA

BRAKE and the linear Birc6 transcript. Because both transcripts originate from the same genomic locus, they may be subject to shared upstream transcriptional regulation. However, their relative abundance is likely determined by distinct, and potentially competing, post-transcriptional processes, namely canonical splicing for linear Birc6 mRNA and back-splicing for circRNA BRAKE. Our data show that deletion of the Alu2–4 elements markedly reduces circRNA BRAKE while leaving linear Birc6 mRNA and BIRC6 protein expression unchanged. This finding supports an important role of the Alu2–4 elements in circRNA BRAKE back-splicing and biogenesis, while indicating that the circular and linear transcripts are ultimately regulated through distinct post-transcriptional mechanisms. The corresponding Discussion sections have been revised accordingly.

Major comment #4: Figure 3. For 3I, please show images with all samples. 3J, the adipocyte diameter analysis was from 3 cells? More cells should be quantified.

Figure 3I presents representative gross images of eWAT from control and AKD mice. Per the reviewer's suggestion, to provide a more comprehensive overview of the tissue phenotypes, we have added representative gross images of iWAT, BAT, and liver from control and AKD mice to Figure S4D.

We also clarified that the adipocyte diameter analysis in Figure 3J was not based on three individual cells. For each mouse, all clearly delineated adipocytes within at least three random, non-overlapping fields were measured, corresponding to approximately 20–40 adipocytes per field and 80–160 adipocytes per mouse, depending on adipocyte size. The mean adipocyte diameter was then calculated for each mouse, and each data point in Figure 3J represents one individual mouse. We have increased the number of analyzed mice to n = 6 per group and revised the figure legend and Methods section accordingly.

Major comment #5: Figure 4 and Figure 5. The authors demonstrate that BRAKE affects the mRNA levels of specific GO pathways in fig. 4, and then display BRAKE interact with ATP5A protein in fig. 5. The authors need to at least provide some solid references to support that the ATP5A–mTORC1 pathway eventually regulates the gene expression of specific GO pathways in

fig. 4. If there are not enough supporting information, the authors need to provide some experimental data.

We agree that the connection between the transcriptional programs identified in Figure 4 and the ATP5A–mTORC1 mechanism in Figure 5 should be further supported. To address this issue, we performed additional rapamycin rescue experiments in circRNA BRAKE-knockdown APCs. Rapamycin attenuated the circRNA BRAKE knockdown-induced upregulation of adipogenesis- and lipid synthesis-related genes, reduced PPARγ protein expression and FFA release, and attenuated the increase in OXPHOS protein abundance (Figures S8A–D). Consistently, rapamycin also blocked the enhancement of adipogenic differentiation induced by circRNA BRAKE knockdown (Figure 5S). These new data support that mTORC1 activation contributes to the increased OXPHOS protein abundance and enhanced adipogenic differentiation induced by circRNA BRAKE knockdown. We have added these results to the revised manuscript.

Major comment #6: Figure 6. In the figure legends of 6C, 6D, and 6F, and also in the main text, not sure what "mitochondrial expression of circRNA BRAKE" means? Should it be "mitochondrial levels of circRNA BRAKE"?

We thank the reviewer for pointing this out. We agree that the phrase "mitochondrial expression of circRNA BRAKE" may be misleading because circRNA BRAKE is encoded by the nuclear genome rather than expressed from the mitochondrial genome. We have therefore revised the figure legends for Figures 6C,6D and 6F, as well as the corresponding descriptions in the main text, to use more precise wording "mitochondrial levels of circRNA BRAKE".

RESPONSE TO REVIEWER 3

General comment: The manuscript investigates the functional role of a mitochondria-localized circular RNA, termed circRNA BRAKE, in the regulation of adipogenesis and metabolic homeostasis under conditions of nutrient overload. Using a comprehensive experimental approach that integrates genetically modified mouse models, in vitro adipocyte systems, transcriptomic profiling, mitochondrial bioenergetic analyses, and RNA–protein interaction assays, the authors demonstrate that circRNA BRAKE directly interacts with the ATP synthase subunit ATP5A. This interaction disrupts Complex V assembly, reduces ATP production, and suppresses adipogenesis through inhibition of mTORC1 signaling. Overall, the study addresses an important and timely question regarding the role of mitochondrial circRNAs in metabolic regulation. While the data are extensive and largely convincing, several methodological and interpretative issues require clarification to strengthen the rigor and reproducibility of the study.

Major comment #1: Studies showed a clear heterogeneity of white adipose tissues in both humans and mice, with significant differences regarding energy metabolism and mitochondrial regulation (PMID: 26964831, 34935088, 29507687). Considering that eWAT showed a lower APC deposition and less response in mitochondrial metabolism during obesity compared to BAT or subcutaneous WAT, why was the eWAT chosen in the study to be investigated? This matter should be clear in the manuscript.

We thank the reviewer for raising this important point. We agree that WAT exhibits marked depot-specific heterogeneity in adipogenic capacity, energy metabolism, and mitochondrial regulation. We focused on eWAT because it is a major visceral-like adipose depot in mice that is closely associated with obesity-related metabolic dysfunction. Under nutrient overload, eWAT is particularly susceptible to pathological stress-related remodeling, whereas BAT and subcutaneous iWAT are generally associated with thermogenic or metabolically protective adaptations [1-3]*. Thus, eWAT provides a more relevant depot for investigating maladaptive adipose responses in obesity.*

Importantly, despite its lower mitochondrial abundance than BAT, mitochondrial homeostasis in eWAT remains critical for systemic metabolism. Consistent with this concept, our previous study showed that adipose-specific Trx2 deficiency disrupted mitochondrial homeostasis in eWAT and promoted hepatic insulin resistance and hyperglycemia[4].

Furthermore, our depot-comparison analysis showed that circRNA BRAKE abundance was highest in eWAT compared with iWAT and BAT (Figure 2G), with significant upregulation observed exclusively in the mitochondria of eWAT from ob/ob mice (Figures 1E and 1G), providing additional direct support for investigating the function of circRNA BRAKE in eWAT.

Therefore, eWAT provides a biologically relevant tissue for investigating how mitochondria-localized circRNA BRAKE regulation of ATP synthase contributes to obesity-associated adipose dysfunction and systemic metabolic abnormalities. We have clarified this rationale in the revised manuscript.

Major comment #2: The circRNA BRAKE knockdown occurs exclusively in mature adipocytes due to the use of the Adiponectin-Cre system. Nevertheless, several conclusions extend to adipogenic processes occurring at earlier stages in vivo. Although in vitro experiments partially address this limitation, the manuscript would benefit from a clearer acknowledgment of this constraint and a more cautious interpretation of in vivo adipogenesis-related findings.

We thank the reviewer for this thoughtful comment. We agree that Adiponectin-Cre–mediated circRNA BRAKE knockdown occurs predominantly in mature adipocytes and therefore has limited ability to directly define the role of circRNA BRAKE during early adipogenesis in vivo. We have revised the manuscript to interpret the in vivo adipogenesis-related findings more cautiously. We also added a statement in the Discussion acknowledging this limitation, while noting that our primary APCs experiments support a cell-autonomous role of circRNA BRAKE in regulating adipogenic differentiation. We further clarified that future studies using progenitor-targeting or inducible Cre models will be needed to determine the stage-specific function of circRNA BRAKE during adipose tissue development and remodeling.

Major comment #3: In the figure 4 L, the authors described “Seahorse XF24 analysis confirmed

enhanced maximal oxygen consumption rate (OCR) in AKD APCs", however, there is no statistical symbol in the figure. What is the number of samples used in this experiment?

In the original Seahorse XF24 experiment, the sample size was n = 3 biological replicates per group, with each replicate derived from eWAT APCs isolated from one independent mouse. We agree that this sample size was limited and therefore repeated and expanded the experiment to n = 6 independent mice per group. Each data point in the revised Figure 4L represents the mean value of differentiated adipocytes derived from one individual mouse, rather than a technical replicate. Statistical significance symbols have now been added to Figure 4L, and the corresponding figure legend and Methods section have been revised to clearly state the sample size and biological replicate information.

Major comment #4: In the figure 4M, the absence of circRNA BRAKE in AKD increased the protein content of the components of the mitochondrial complexes encoded by nuclear and mitochondrial DNA. How does circRNA BRAKE promote these effects?

To answer the reviewer's question, we performed additional experiments in differentiated APCs from control and AKD mice with or without rapamycin treatment. Consistent with our original observation, AKD increased the abundance of multiple OXPHOS proteins. Importantly, rapamycin treatment attenuated the AKD-induced increase in OXPHOS protein levels (Figure S8D), suggesting that these changes are at least partly dependent on mTORC1 activity. Together with our data showing that circRNA BRAKE knockdown enhances ATP production and activates mTORC1 signaling, these results support the interpretation that the increased OXPHOS protein abundance in Figure 4M reflects enhanced mTORC1-associated mitochondrial functional maturation during adipocyte differentiation, rather than direct regulation of each OXPHOS complex by circRNA BRAKE.

Major comment #5: The data presented do not allow us to conclude that circRNA BRAKE alters ATPase assembly. Although ATPase activity has been monitored, ATPase integrity requires other techniques. For example, the Blue Native page (BN-page) assay would allow the authors to state that circRNA BRAKE affects ATPase assembly.

We agree that ATP synthase activity alone is insufficient to conclude directly that circRNA BRAKE alters ATP synthase assembly. To address this concern, we performed Blue Native PAGE (BN-PAGE) to assess the assembly and integrity of native OXPHOS complexes, including ATP synthase, in cells overexpressing wild-type circRNA BRAKE (Figure 5H) or the ATP5A-binding-deficient circRNA BRAKE mutant (Figure 5N).

BN-PAGE showed that wild-type circRNA BRAKE reduced the abundance of intact ATP synthase complexes, whereas deletion of the ATP5A-binding region largely abolished this effect. These results provide direct biochemical evidence supporting the conclusion that circRNA BRAKE affects ATP synthase assembly and integrity in an ATP5A-binding-dependent manner. We have added these new data to Figure 5 and revised the Results and Methods sections accordingly.

Major comment #6: The link between ATP production and mTORC1 signaling needs to be explored.

To directly explore the relationship between ATP production and mTORC1 signaling, we performed additional experiments using oligomycin, a pharmacological inhibitor of mitochondrial ATP synthase, in differentiated APCs. CircRNA BRAKE knockdown increased ATP production and elevated pS6K/S6K levels, whereas oligomycin treatment attenuated both ATP production and the increase in pS6K/S6K (Figure S7J). These results support that enhanced ATP production contributes to mTORC1 activation downstream of circRNA BRAKE knockdown.

Major comment #7: It was attested in the results (line 122, figure 1I) that immunofluorescence analysis was used to identify circRNA BRAKE accumulation in eWAT, but the method used is not in the methodology of the study. The immunofluorescence protocol should be included in the methodology.

The experiment shown in Figure 1I was performed using circRNA BRAKE fluorescence in situ hybridization (FISH) combined with TOM20 immunofluorescence co-staining. Although the FISH procedure was described in the original Methods section, we agree that the immunofluorescence component was not described with sufficient clarity. We have therefore revised the Methods

subsection title to "Fluorescence in situ hybridization and mitochondrial immunofluorescence co-staining" and expanded the protocol. The revised text reads as follows:

"Fresh-frozen eWAT sections and 3T3-L1 cells were processed for circRNA BRAKE fluorescence in situ hybridization (FISH) combined with mitochondrial immunofluorescence staining. Briefly, samples were fixed in 4% paraformaldehyde, permeabilized with 0.05% Triton X-100, and dehydrated through an ascending ethanol series. After prehybridization in 2× SSC at 55°C, samples were hybridized overnight with digoxigenin-labeled oligonucleotide probes (Synbio Technologies) targeting the back-splice junction of circRNA BRAKE. After stringent washing, FISH signals were detected using an FITC-conjugated anti-digoxigenin antibody (1:200, Cat. No. 11207741910, Roche), and nuclei were counterstained with DAPI (Cat. No. 0100, SouthernBiotech). For mitochondrial immunofluorescence co-staining, samples were blocked with 0.5% BSA in PBST and then incubated with an anti-TOM20 antibody (1:500, Cat. No. 11802-1-AP, Proteintech), followed by a Cy3-conjugated goat anti-rabbit IgG secondary antibody (1:200, Cat. No. GB21303, Servicebio). Images were acquired using a Leica TCS SP8 STED 3X confocal microscope, and three-dimensional reconstruction was performed using Leica software. The sequences of the FISH probes are provided in Table S2."

Minor comment #1: Terminology related to circRNA BRAKE (e.g., circBirc6 versus circRNA BRAKE) should be standardized throughout the manuscript after the initial definition.

Per the reviewer's suggestion, we have standardized the terminology throughout the manuscript. After the initial definition of circBirc6 as circRNA BRAKE, we consistently use "circRNA BRAKE" to refer to the circular RNA. When referring to the corresponding host linear transcript, we define it as "linear Birc6 transcript corresponding to circRNA BRAKE" and use "linear BRAKE" only thereafter for readability. "BIRC6 protein" is retained specifically for the host-gene-encoded protein.

Minor comment #2: The authors could consider replacing the term Complex V with ATPase throughout the text.

Per the reviewer's suggestion, we have revised the terminology throughout the manuscript and replaced "Complex V" with "ATP synthase (ATPase)" where appropriate to improve consistency and clarity.

Minor comment #3: Minor typographical and formatting issues are present (e.g., missing spaces, concatenated words, and inconsistent unit formatting) and should be corrected.

We thank the reviewer for this comment. We have carefully checked the manuscript and corrected typographical, spacing, and formatting errors throughout the revised version.

Minor comment #4: Some writing errors were found, for example: mROC1 (mTORC1), line 97 and "funciton", line 99.

We have carefully proofread the manuscript and corrected the indicated typographical errors, including "mROC1" to "mTORC1" and "funciton" to "function". We also corrected additional spelling, spacing, and formatting errors throughout the manuscript.

Minor comment #5: The Discussion section could be strengthened by a more direct comparison

with previously described mitochondria-localized circRNAs, highlighting similarities and differences in mechanism.

Per the reviewer's suggestion, we have revised the Discussion to more directly compare circRNA BRAKE with previously described mitochondria-localized circRNAs. Please find the revised text as follows: "Our findings are also consistent with emerging evidence that mitochondria-associated circRNAs can interact with ATP synthase subunits. For instance, the mitochondrial genome-encoded circRNA SCAR was reported to bind ATP5B and regulate mPTP-associated mitochondrial ROS production. Recent work further demonstrated that multiple mecciRNAs interact with mPTP-related proteins, including ATP5B 17,53. Compared with these mitochondria-encoded circRNAs, circRNA BRAKE represents a nuclear genome-derived mitochondria-localized circRNA that directly targets ATP5A, disrupts the ATP5A–ATP5B interaction, and regulates ATP synthase assembly in adipocytes. Thus, our study extends the functional spectrum of nuclear genome-derived mitochondria-localized circRNAs in regulating mitochondrial protein interactions, bioenergetic function, and systemic metabolism."

Minor comment #6: In some sections, particularly the Introduction and Discussion, the tone could be slightly moderated to avoid overly definitive statements and better reflect the mechanistic and exploratory nature of the study.

Per the reviewer's suggestion, we have carefully revised the Introduction and Discussion to moderate the tone of several statements and to better reflect the mechanistic and exploratory nature of our study.

Minor comment #7: Figure S1. Student's t-test used in this figure is not appropriate.

We agree that Student's t-test was not appropriate for Figure S1 because each marker includes multiple groups defined by genotype and subcellular fraction. Since mtCOX2 and β-Actin serve as distinct markers for mitochondrial and cytosolic fractions, respectively, we did not perform direct statistical comparisons between these two markers. Instead, we reanalyzed each marker separately using two-way ANOVA followed by Šídák's multiple comparisons test to compare the relevant genotype- and fraction-based groups. The statistical annotations and figure legend have been

updated accordingly.

Minor comment #8: Figure S3. The description in the caption for figures S3C and F is reversed. Statistical symbols are missing in Figure S3F. Furthermore, the number of samples used in the mitochondrial respiration experiment is too low.

Per the reviewer's suggestion, we have corrected the reversed descriptions in the captions for Figures S3C and S3F. We also added statistical analyses and significance symbols for the Seahorse experiments, including basal and maximal respiration quantification in both Figure 4L and Figure S3F. In addition, we agree that the original sample size for the mitochondrial respiration experiments was relatively limited. We therefore expanded the analyses using differentiated adipocytes derived from eWAT APCs isolated from six independent mice per group. Each dot represents the mean value of differentiated adipocytes derived from one individual mouse, rather than technical replicates from the same mouse. These revisions and new analyses have been incorporated into the updated figures and figure legends.

References

1. Wang, T., Sharma, A. K. & Wolfrum, C. Novel insights into adipose tissue heterogeneity. *Rev. Endocr. Metab. Disord.* **23**, 5-12 (2022).
2. Kwok, K. H. M., Lam, K. S. L. & Xu, A. Heterogeneity of white adipose tissue: molecular basis and clinical implications. *Experimental & Molecular Medicine*. **48**, e215 (2016).
3. Wijayatunga, N. N. et al. An integrative transcriptomic approach to identify depot differences in genes and microRNAs in adipose tissues from high fat fed mice. *Oncotarget*. **9**, 9246-9261 (2018).
4. He, F. et al. Mitophagy-mediated adipose inflammation contributes to type 2 diabetes with hepatic insulin resistance. *The Journal of Experimental Medicine*. **218**, e20201416 (2021).

RESPONSE TO REVIEWER 3 ver.2

General comment: The authors have satisfactorily addressed most of my major concerns and have substantially strengthened the manuscript by performing several additional experiments (including BN-PAGE, rapamycin and oligomycin treatments, expanded Seahorse analyses, and methodological controls). I particularly appreciate that the authors explicitly acknowledged the limitations of the Adiponectin-Cre model and appropriately moderated the interpretation of the in vivo adipogenesis data. Likewise, the oligomycin experiments provide important mechanistic support for the proposed relationship between ATP production and mTORC1 activation. Overall, the revised manuscript is substantially improved, the conclusions are adequately supported by the available data, and I have no further major concerns. I believe the manuscript is suitable for publication after minor editorial revisions.

Minor comment #1: One point that remains only partially resolved concerns the mechanism underlying the increased abundance of OXPHOS proteins following circRNA BRAKE knockdown. The new rapamycin experiments convincingly demonstrate that these changes are at least partly mTORC1-dependent. However, they do not fully explain how proteins encoded by the mitochondrial genome are coordinately increased, given that mTORC1 primarily regulates cytosolic protein synthesis. Although this mechanistic aspect remains incompletely understood, I do not believe that it detracts from the main conclusions of the study.

We agree that, although rapamycin attenuated the increase in OXPHOS protein abundance after circRNA BRAKE knockdown, this does not fully explain how mitochondria-encoded subunits are coordinately increased. We have therefore revised the Discussion to clarify that mTORC1 may promote the abundance of nuclear-encoded OXPHOS proteins through 4E-BP-dependent translational regulation of mitochondrial proteins, whereas the increase in mitochondria-encoded MTCO1 cannot be explained by direct mTORC1-mediated cytosolic translation. We now interpret these changes as reflecting broader mitochondrial functional maturation and nuclear–mitochondrial coordination, potentially involving mitochondrial translation, protein stability, or assembly-dependent stabilization of respiratory complexes. We have also

acknowledged that the precise mechanism remains to be further investigated.

References

1. Wang, T., Sharma, A. K. & Wolfrum, C. Novel insights into adipose tissue heterogeneity. *Rev. Endocr. Metab. Disord.* **23**, 5-12 (2022).
2. Kwok, K. H. M., Lam, K. S. L. & Xu, A. Heterogeneity of white adipose tissue: molecular basis and clinical implications. *Experimental & Molecular Medicine*. **48**, e215 (2016).
3. Wijayatunga, N. N. et al. An integrative transcriptomic approach to identify depot differences in genes and microRNAs in adipose tissues from high fat fed mice. *Oncotarget*. **9**, 9246-9261 (2018).
4. He, F. et al. Mitophagy-mediated adipose inflammation contributes to type 2 diabetes with hepatic insulin resistance. *The Journal of Experimental Medicine*. **218**, e20201416 (2021).
5. Morita, M. et al. mTORC1 controls mitochondrial activity and biogenesis through 4E-BP-dependent translational regulation. Cell Metab. 18, 698-711 (2013).
6. Cunningham, J. T. et al. mTOR controls mitochondrial oxidative function through a YY1-PGC-1alpha transcriptional complex. Nature. 450, 736-740 (2007).
